# A Material Study of Persian-Period Silver Coins and Hacksilber from Samaria

**DOI:** 10.3390/ma18071678

**Published:** 2025-04-07

**Authors:** Dana Ashkenazi, Maayan Cohen, Haim Gitler, Mati Johananoff, Oren Tal

**Affiliations:** 1 Tel Aviv University, Ramat Aviv 6997801, Israel; 2 Department of Archaeology and Ancient Near Eastern Cultures, Tel Aviv University, Ramat Aviv 6997801, Israel; maayancohen8@gmail.com (M.C.); johananoff@gmail.com (M.J.); orental@tauex.tau.ac.il (O.T.); 3 Leon Recanati Institute for Maritime Studies, University of Haifa, Haifa 3498838, Israel; 4 The Israel Museum, Derech Rupin 11, Jerusalem 9171002, Israel; gitler@imj.org.il

**Keywords:** archaeometallurgy, hacksilber, microstructure, SEM-EDS analysis, silver coins, Persian (Achaemenid) period, Samaria, southern Levant

## Abstract

An assembly of fourth-century BCE Samarian silver coins and late-fifth-century BCE Samarian cut silver sheets, Sidonian and Philistian coins from a hacksilber hoard allegedly found in the region of Samaria belonging to the David and Jemima Jeselsohn collection, were characterized by metallurgical analyses. The aims of the research were to identify the items’ composition and manufacturing processes. We affirmed that the Samarian coins were made of silver–copper alloy produced by a controlled process. The microstructural and elemental analyses revealed that the sheets were produced from various materials, including pure silver, silver–copper, and silver–copper–gold alloys, whereas the Sidonian and Philistian coins were made of silver–copper alloy. Continuity in style and production techniques was observed. This information provides a better understanding of the material culture and technological skills in the Persian-period province of Samaria.

## 1. Introduction

The earliest known silver (Ag) artifacts date back to the fourth millennium BCE. By the middle of the third millennium BCE, silver was already a valuable trade commodity. It is presumed that the earliest silver objects were smelted directly from silver ores and therefore did not contain lead (Pb) [1]. Historically, silver was commonly derived from lead sulfide (PbS) silver-rich galena minerals containing approximately 1–2 weight percent (wt%) Ag through the cupellation process, known for yielding silver metal of over 95 wt% purity [2,3].

Cupellation is a metallurgical process of refining ores, that was used to separate silver from lead and other metals through oxidizing and removing impurities at high temperatures. The cupellation process is very effective in producing high-purity silver; however, the refined metal often contains minor-to-trace amounts of the elements Pb, Au, Bi, and Cu, as well as traces of Sb, As, Zn, and Ni [4,5]. In order to increase efficiency, the cupellation process involved three separate hearths. In the first furnace (smelting hearth), which was used for smelting lead–silver ores, such as galena, wood fuel remelted impure lead (bullion) containing silver at high temperature, oxidizing to litharge (PbO) inside a bellows-powered tuyères [1,2]. Additional bullion was added until molten silver-enriched lead formed which was moved to a second furnace (cupellation hearth) and oxidized again in order to separate silver from lead through oxidation. Litharge was removed by dipping iron bars into the crucible to form coated litharge cones. This process was repeated until silver globules remained. Finally, in the third furnace (litharge collection hearth), the globules were melted and refined into sliver ingots, while residual lead oxide was absorbed in the cupel’s porous wall [1,2]. The refined silver was then cast into ingots and often used to produce coins [2].

Non-argentiferous lead, a relatively pure lead with little silver, was sometimes used in cupellation. This lead has an affinity for sulfur rather than for oxygen and produces silver alloys with low gold content when derived from gold-bearing sources [6]. An incomplete cupellation process results in silver alloys with small wt% of Pb [7], which, after long burial, leads to brittleness. Thus, well-preserved ancient silver artifacts often lack lead [2], suggesting a successful cupellation with fully refined silver [1].

Silver and its alloys were frequently used by early civilizations to produce coins and manufacture artifacts such as ornaments and jewelry [8]. For that purpose, sophisticated technological skills were needed, such as casting, a cycle of cold working (hammering) and heating (annealing), granulation, and joining techniques [9]. Silver was often alloyed with copper (Cu) to improve the mechanical properties and as a melting-point depressant. Since copper diffuses easily in silver, it is commonly applied as a joining material (filler) of silver parts, such as jewelry [8,10,11]. The presence of more than 2.5–3 wt% Cu in ancient silver items is considered evidence that the copper was intentionally added to the silver metal [12,13,14].

Despite the utility of scanning electron microscopy (SEM) as an important non-destructive surface-imaging technique with energy dispersive spectroscopy (EDS) for surface elemental analysis, it poses challenges due to factors that affect metal surface composition, such as long-term corrosion processes, silver enrichment on the external surface, presence of oxide layers and tarnish, and remnants of cleaning agents [2,15,16,17]. Therefore, the external surface of ancient silver coins often contains compounds such as silver oxide (Ag_2_O), silver sulfide (Ag_2_S), and/or silver chloride (AgCl) [2]. Consequently, the EDS elemental analysis results of the external surface of a silver alloy object might differ significantly from its bulk composition [18]. For example, copper near the external surface of ancient silver items is vulnerable to corrosion in aggressive environments after prolonged burial periods, leading to an externally enriched silver surface [19]. In addition, even when bulk material composition of ancient silver objects is detected by destructive testing methods, there may be uncertainties about elemental analysis results due to heterogeneity of the examined samples. Therefore, it is recommended that each object is measured in several areas to reveal its elemental composition [2].

Despite the challenges due to environmental factors, surface analysis can be used to characterize silver alloy items without additional polishing of the external surface when the remaining oxide and corrosion cover is thin enough [14,15,16,20]. For example, in a study focusing on SEM-EDS non-destructive testing (NDT) analysis of high-purity Yehud silver coins of the late-Persian and early-Hellenistic period, dated between the second half of the fourth century and the first half of the third century BCE, Cohen et al. [2] found that the copper content on the surface of well-preserved bright areas (according to BSE mode) correlated well with the bulk copper content following grinding, indicating that the SEM-EDS analysis of the coin surfaces mirrored the coin’s overall composition. Yet, according to Cohen at al., compositional data obtained from the surface of coins reflected the bulk composition only when that coin was made of relatively pure silver (greater than 92 wt% Ag) and was well preserved [2,21]. SEM-EDS analysis of the external surface and the ground bulk of the jewelry from the Samaria and Nablus Hoards also lead to the conclusion that the surface composition of well-preserved shiny metal represents their bulk composition [15,16]. In addition, X-ray fluorescence (XRF) and inductively coupled plasma (ICP) with atomic emission spectrometry (AES) analyses of southern Levantine/Palestinian Persian-period silver coins also support this conclusion [13]. Yet, if the examined object is covered with a thick oxide cover and massive corrosion products, the SEM-EDS analysis of the surface does not provide a representative characterization of the bulk alloy composition [20,22]. Therefore, if possible, it is recommended to use destructive methods, such as SEM-EDS examination of a standard metallographic cross-section and/or ICP-AES analysis, to examine silver objects [15,16].

Throughout the two centuries of Achaemenid rule in the Near East (538–332 BCE), the region experienced profound economic, political, and cultural transformations. A notable development during this period was the introduction of locally minted coinages. The Achaemenid era was marked by the coexistence of coins issued by different minting authorities, each fulfilling specific economic functions. The objective of this current study is to leverage NDT analysis to offer insights into a late-fifth-century BCE hacksilber hoard allegedly found in the region of Samaria and fourth-century BCE Samarian silver coins, while advancing our understanding of ancient techniques employed in producing minute silver coins during the Persian period in the southern Levant and specifically in the province of Samaria.

### 1.1. The Production Process of Persian-Period Silver Coins from the Southern Levant

Throughout antiquity, silver alloys were used by official authorities to produce coins; hence, the study of silver coins can assist in gaining insights into the economic, political, and social aspects of ancient civilizations and link political and economic events of a specific period [19,23,24,25,26].

Copper was the main alloying element used by ancient authorities for the production of silver coins, with various ratios of silver and copper. Elevated copper content in silver coins over about ~2.5–3 wt% Cu indicates that these coins were intentionally alloyed with copper to lower the melting point and enhance mechanical properties of the alloy [10,11,15,16]. Periods of economic instability resulted in the debasement of coinage, namely silver content decreased while copper content rose [1]. Analyzing the chemical composition and homogeneity of coins exposes their alloy, offering insights into whether coins were crafted with specific metallurgical compositions or processes [2]. Matching a coin’s chemical composition with authenticated data can potentially pinpoint its production origin [2,27].

Ancient silver coins were usually produced from cast flans, where an individually heated flan was struck with a high-tin bronze alloy die [28]. When there was a shortage of skillful die engravers, worn or damaged dies were employed [2]. The reverse die (punch), situated on the hammer side, typically deteriorated earlier than the lower obverse die (anvil) [26,29,30,31].

**Figure 1:**
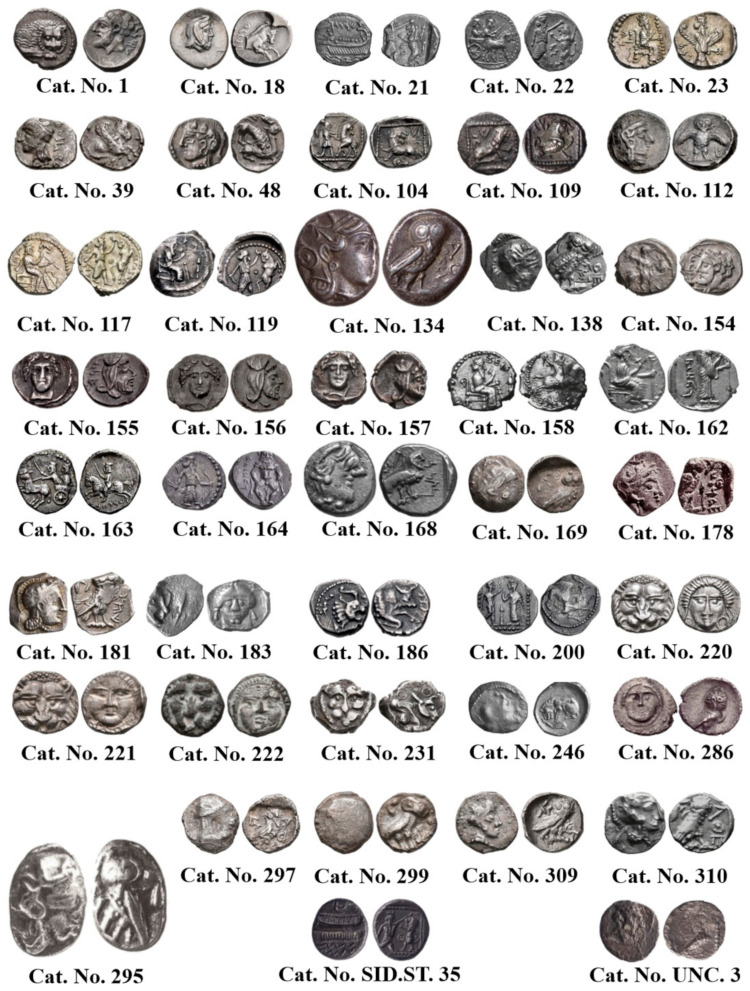
Fourth-century BCE Samarian silver coins analyzed in this paper.

In the process of compiling an updated corpus of Samarian coin types, numerous Samarian coins held in public collections (specifically those from The Israel Museum, Jerusalem [hereafter IMJ]) underwent metallurgical analysis using NDT methods, as outlined in the protocol below (Figure 1 and Table 1). The 35 groups of coin types are arranged according to Cat. Nos. reflecting the new typological division of Samarian coin types which will appear in a forthcoming corpus of this coinage [32].

### 1.2. Late Persian-Period Silver Hoards from Samaria

Fourth-century BCE silver jewelry and coins from the Samaria and the Nablus Hoards (hereafter SH and NH, respectively) have previously been studied by metallurgical NDT. Both hoards appeared in the antiquity market in 1968 and are characteristic of the partially monetized economy of the southern Levant during the period [15,16,33,34]. The SH was found in a pottery container, comprising 334 coins and 78 pieces of jewelry, and is partly in the possession of the Israel Museum collection (the pottery container, 34 coins and all the jewelry). The NH, which contained some 750 coins and jewelry, has been dispersed between various public and private collections. A significant part of this hoard is currently on loan at the Israel Museum. The SH silver jewelry included a face-shaped pendant; two rectangular pendants; a spiral ring; a smooth, round ring; a rounded bead; a jewelry fragment; a silver and vitreous material cylinder; a pendant with a glass bead; and a bead made of small granules. The NH silver jewelry included a bar with two rings; a spiral bead; four leaf-shaped pendants; a spatula-shaped pendant; a coned pendant; an omega-shaped pendant; a decorated ring; beads made of small granules; an electrum bar with nine granules; and two decorated earrings [15,16].

The burial date of the SH was around 352 BCE, whereas that of the NH is estimated to have occurred around 331 BCE or sometime later. The average copper concentration in the silver alloy of the jewelry from the SH and the NH (after excluding corrosion products and soil elements) was 5.8 ± 3.0 wt% Cu and 5.0 ± 3.2 wt% Cu, respectively, which strongly suggests that the silver bullion was intentionally alloyed with copper. This variability is almost certainly due to the use of a surface-only analytical technique [35]. Nevertheless, higher copper concentrations were found in brazing and contact melting joints of the items, with an average copper concentration of 17.7 ± 10.7 wt% Cu [15,16,33]. The manufacturing processes of the jewelry employed in the SH and NH shared technological similarities, with the use of methods such as casting, sawing, bending, twisting, hammering, plastically deforming the metal, and granulating, as well as joining methods. These shared practices suggest continuity in local jewelry production technologies and style during the Persian period [15,16,33].

**Figure 2:**
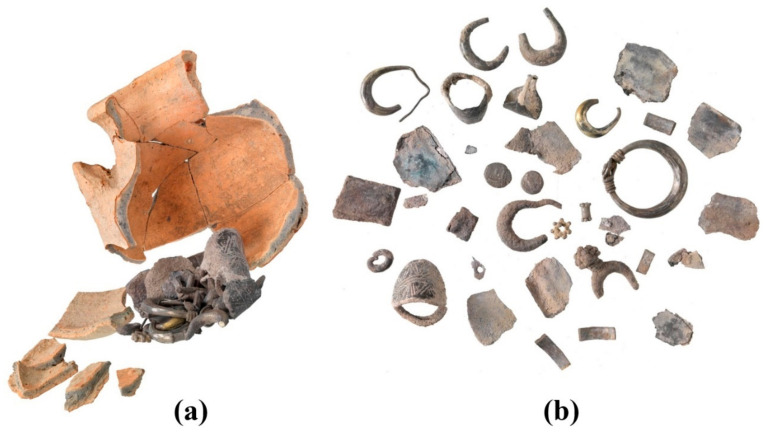
The late-fifth-century BCE Samaria region hacksilber hoard: (**a**) reconstruction of the hoard assembled within its original container and (**b**) assemblage of hoarded items, including silver alloy jewelry, hacksilber, and coins (Photos by Y. Gunzenreiner, Leu Numismatik A.G.).

**Figure 3:**
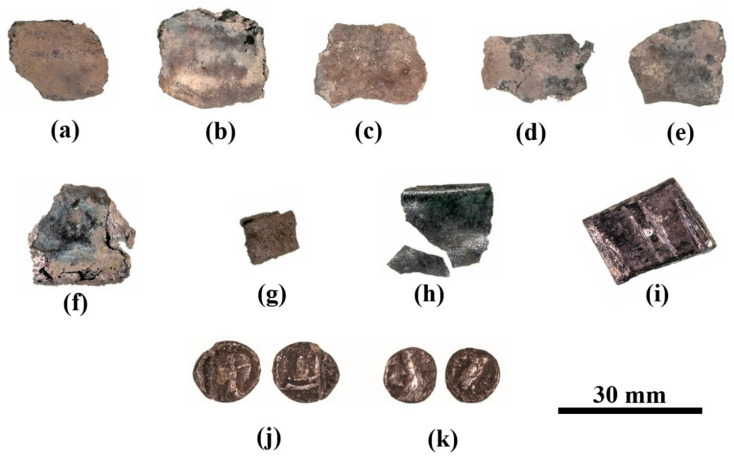
The late-fifth-century BCE Samaria region hacksilber hoard: (**a**–**h**) thin silver sheets (Samples 15–22); (**i**) block-shaped striated ingot(?) (Sample 23); (**j**) Sidonian coin (Sample 24); and (**k**) Philistian coin (Sample 25).

Hacksilber hoards, such as the one examined here (Figure 2), have been studied in the past decades [36,37,38] and some scholars consider these fragments of cut silver ingots as a flexible medium of exchange for goods and services that was used during antiquity in pre-monetary economic periods [39]. Nonetheless, other scholars consider these fragments as an alternative form of goods in a society of barter-based economy [40]. Hacksilber hoards in the Levant are known from the Middle Bronze Age [38,40], but were more common in the Iron Age [39,41]. Hacksilber hoards that were discovered together with broken jewelry and cut coins (essentially hacksilber) are dated primarily to the later sixth, fifth, and fourth centuries BCE. Hacksilber hoards, often stored inside ceramic containers or pots, have been discovered at archeological sites throughout the southern Levant and are also commonly found in the antiquities market [42,43,44]. A small silver hoard consisting of 25 silver alloy items, including jewelry, hacksilber, and a Sidonian coin and a Philistian coin (Figure 3 and Table 2) was found within a ceramic pyxis in Samaria in the 1970s (Figure 2) and was previously studied by typological and archaeo-metallurgical perspectives [42], while focusing on the analysis of the jewelry from the hoard. The late-fifth-century BCE Sidonian and Philistian coins (Figure 3 and Table 2) from the hoard provided an approximate burial date of the hoard. The jewelry belonging to the hacksilber hoard from the Samaria region includes an earring or perhaps a ring decorated with a flower like rod; five lunate earrings; one lunate earring decorated with granules; a bead made of granules; a spiral bead; three rings with a decorative oval bezel; and parts of a broken ring (Figure 1 and Table 2) [42].

## 2. Experimental Methods and Methodology

NDT methods were utilized to analyze Samarian silver coins and hacksilber pieces allegedly found in the area of Samaria (Figure 1 and Figure 3):

(a) The silver coins and silver alloy fragments underwent visual testing (VT) to uncover macroscopic features indicative of their preservation state, assess their condition, and better understand their manufacturing methods.
(b) Weight and dimensional measurements were carried out for all items. The objects’ weight was measured using a digital weighing device with a precision scale of 0.001 g, while the dimensions were recorded using a digital caliper. Items that underwent mechanical cleaning before SEM-EDS analysis had their weights documented before and after cleaning.
(c) SEM observation was performed for the silver coins and hacksilber pieces, combined with EDS analysis (ESEM, Quanta 200 FEG instrument, Thermo Fisher Scientific, Waltham, MA, USA) in high vacuum mode with a secondary electron (SE) detector. SE and back-scattered electron (BSE) modes were utilized. Surface composition was determined via EDS, employing a Si(Li) liquid-cooled Oxford X-ray detector. To ascertain the silver alloy composition, only the bright metal regions observed according to BSE mode were analyzed. Areas covered with corrosion products (dark areas according to BSE mode) were not included in the average composition calculations. For each group of coins, the average composition values and standard deviations were calculated by examining six different areas or more (for most coins) on the obverse and reverse sides of each item. In total, 808 SEM-EDS measurements were performed across all the Samarian silver coins from public collections, which were categorized into 35 groups based on their typology. In addition, 88 SEM-EDS measurements were performed for the silver jewelry, 33 measurements were performed for the silver sheets, and 13 measurements were performed for the silver Sidonian and Philistian coins from the hacksilber hoard, which were categorized based on their typology (Table 2).

SEM-EDS examination is useful for surface analysis, but NDT analysis of ancient silver items can be challenging due to factors affecting surface composition [2,15,16,17]. Surface composition may differ significantly from bulk composition [18]. Due to their good corrosion resistance, ancient silver coins are durable [8]. To assess if surface composition reflects the bulk, the surface was ground in small areas, and the Cu wt% concentration before and after grinding was compared using EDS analysis [2]. Therefore, in the current study, the surface of representative silver items was ground with a 320 silicon-carbide grit paper in different small areas to expose the bulk metal and the composition was compared to the initial surface composition. A layer of about 200 µm thickness of oxides and corrosion products such as silver chloride, silver sulfide, silver oxide, and copper oxide [45,46,47,48] was removed from the surface until a shiny silver metal was exposed. For example, six coins (three from Cat. No. 1: IMJ 34127, IMJ 34183, IMJ 34809 and three from Cat. No. 164: IMJ 34778, IMJ 34836, IMJ 34877) were ground and analyzed. The alloy (base metal) composition of each measurement was determined by excluding peaks corresponding to oxides, corrosion products, and soil elements such as O, Si, Cl, Al, Ca, S, K, P, Fe, and Mg directly from the EDS’s Oxford instrument program (INCA Energy EDS X-ray Microanalysis System). The standard deviation (S.D.) of the average alloy composition of each group of coins, jewelry, sheets, and striated ingot was calculated with an Excel spreadsheet by using the STDEV.P (standard deviation population) function that calculates the S.D. for an entire population.

## 3. Results

### 3.1. Characterization of the Materials of the Samarian Coinage

The detailed SEM-EDS analysis results of each coin (obverse and reverse measurements) are presented in the Electronic Supplementary Materials, Tables S1–S13. The SEM-EDS analysis results of the average alloy composition (wt% content) of each of the coins are presented in Table 3 after omitting the peaks of elements such as O, Si, Cl, Na S, K, P, Al, Fe, and Ca, which are related to oxides, corrosion products, and soil elements. The EDS analysis results of the average alloy composition (wt% content) of each group of coins (each Cat. No.) are presented in Table 4.

**Figure 4:**
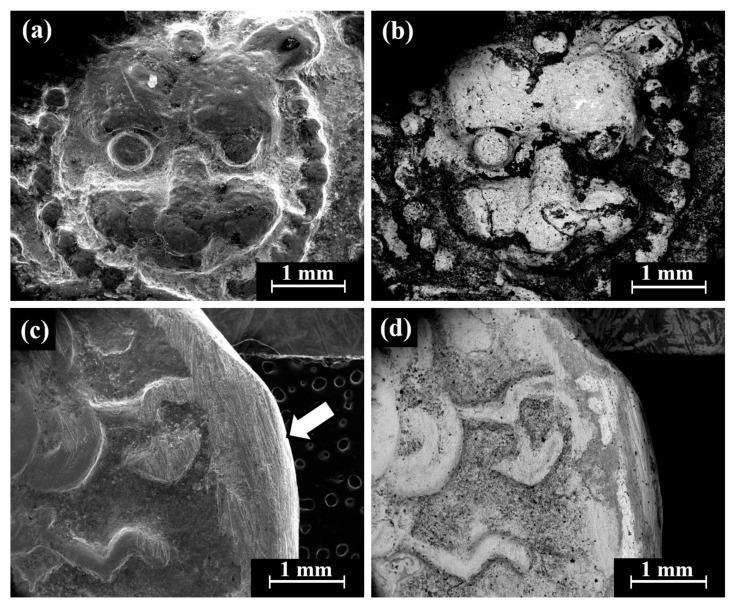
SEM images of Cat. No. 1: (**a**,**b**) obverse of coin IMJ 34809 (SE mode and BSE mode, respectively) and (**c**,**d**) reverse of the same coin (SE mode and BSE mode, respectively), where the white arrow in Figure 4c shows the area that was ground.

Cat. No. 1: SEM observation of coins IMJ 34127, IMJ 34183, and IMJ 34809 (Table 1 and Table 3, Figure 1 and Figure 4a–d) shows the peripheral area of the ground reverse of coin IMJ 34809 (Figure 4c,d, right side of image, exposed bulk metal). Only well-preserved bright areas (according to BSE mode), such as the lion’s forehead (Figure 4b), were included in the average silver alloy composition calculations.

SEM-EDS analysis of the obverse and reverse surfaces of the coins belonging to Cat. No. 1 revealed that they were composed of silver alloyed with copper (Table 3); however, other elements were also detected, including O, Si, Cl, Al, Ca, and S (Electronic Supplementary Materials, Table S1). Cat. No. 1 coins revealed an average composition value of 95.7 ± 3.6 wt% Ag and 4.3 ± 3.6 wt% Cu (24 obverse and reverse measurements were included in the average composition calculations after omitting the peaks of O, Si, Cl, Al, Ca, and S).

**Figure 5:**
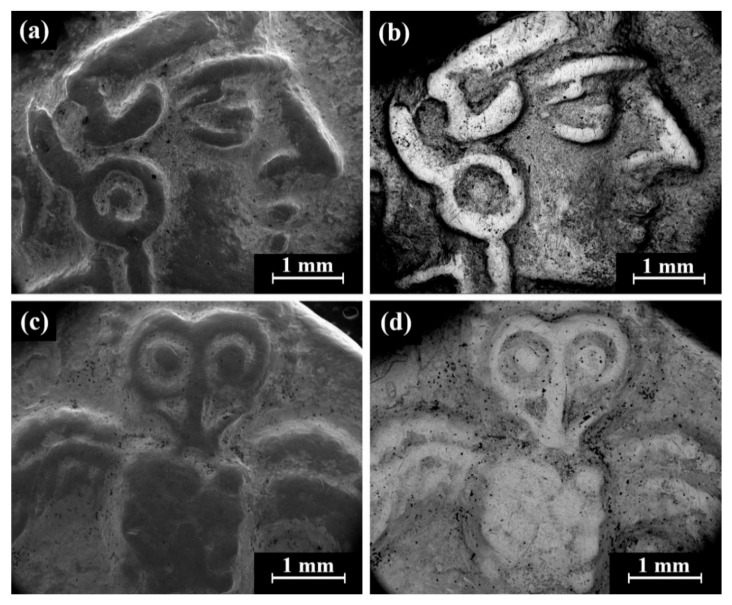
SEM images of the Cat. No. 112: (**a**,**b**) obverse of coin IMJ 34807 (SE mode and BSE mode, respectively) and (**c**,**d**) reverse of the same coin (SE mode and BSE mode, respectively).

**Figure 6:**
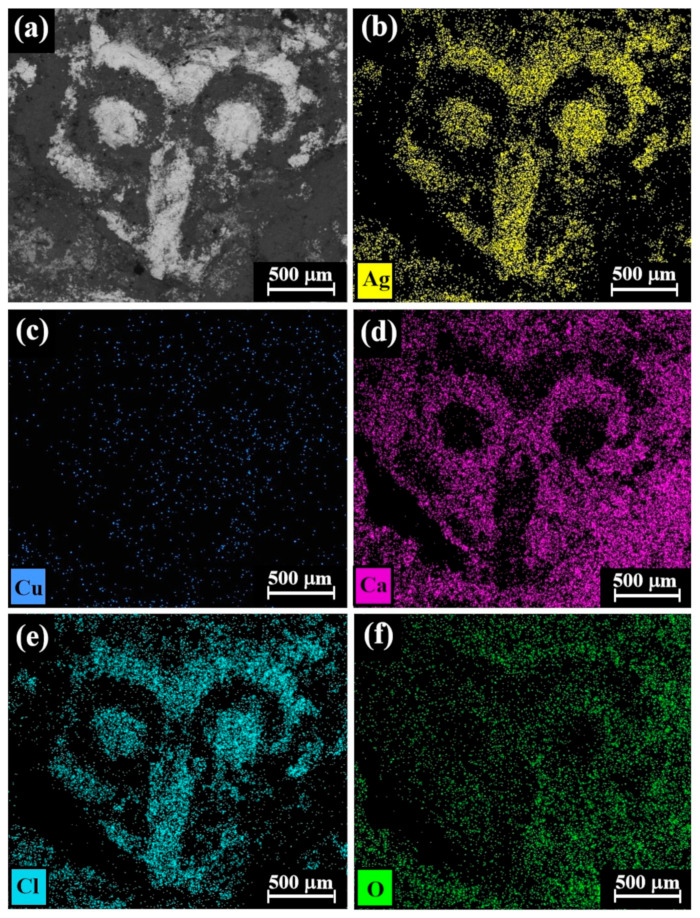
SEM-EDS elemental mapping of Cat. No. 112 (reverse of coin IMJ 34353), showing the presence of (**a**) bright areas of silver alloy and dark areas of corrosion products and soil elements (according to BSE mode) and the presence of the following elements (bright dots): (**b**) silver, (**c**) copper, (**d**) calcium, (**e**) chlorine, and (**f**) oxygen.

Cat. No. 112: SEM observation of coins IMJ 34807, IMJ 34808, IMJ 34353, and IMJ 34354 (Table 1 and Table 3, Figure 1, Figure 5 and Figure 6a) shows the well-preserved bright areas (according to BSE mode) that were included in the average silver alloy composition calculations of Cat. No. 112 (Figure 5b,d, coin IMJ 34807).

The SEM-EDS elemental mapping of the reverse side of coin IMJ 34353 (Figure 6, Cat. No. 112) revealed that the bright areas according to the BSE mode (Figure 6a) are rich in silver and chlorine (Figure 6b and Figure 6e, respectively), whereas the element copper (Figure 6c) is distributed rather homogeneously and the dark areas according to the BSE mode are rich in calcium and oxygen (Figure 6d and Figure 6f, respectively). Since the well-preserved areas of coin IMJ 34353 are rich in chlorine and silver and poor in calcium and oxygen, according to elemental mapping (Figure 6), it is possible that the presence of chlorine is related to the cleaning method of the coin, whereas the presence of calcium is related to the burial environment of the coin. Unfortunately, no documentation about the cleaning procedure of this coin is available.

SEM-EDS analysis results of the obverse and reverse surfaces of Cat. No. 112 issues revealed that they were composed of silver alloyed with copper; however, other elements were also detected, including O, Si, Cl, S, Al, P, and Ca (Electronic Supplementary Materials, Table S3). The measured average alloy composition value of this group was 98.9 ± 2.2 wt% Ag and 1.1 ± 2.2 wt% Cu (23 measurements were included in the calculations after omitting the peaks of O, Si, Cl, S, Al, P, and Ca).

Cat. No. 117: The nine coins of this group (NH 340, NH 343, NH 345, NH 346, NH 347, NH 348, NH 352, NH 355, NH 358; Table 1 and Table 3, and Figure 1) share die-links. NH 340 was struck from the same obverse die as NH 343, NH 346, and NH 345 and the same reverse die as NH 347 and NH 348; NH 343, NH 345, and NH 346 were struck from the same pair of dies; NH 355 and NH 358 were struck from the reverse die. Only areas of bright (according to BSE mode), well-preserved silver alloy were included in the average alloy composition calculations.

SEM-EDS analysis results of the nine Cat. No. 117 group of coins revealed that they were composed of silver alloyed with Cu; however, other elements were also detected, including O, Si, Cl, Na, S, Ca, and Al (Electronic Supplementary Materials, Table S4). The average alloy composition of the coins, after omitting the peaks of O, Si, Cl, Na, S, Ca, and Al, was 94.4 ± 3.7 wt% Ag and 5.6 ± 3.7 wt% Cu (74 measurements of Cat. No. 117 coins were included in the calculations).

Cat. No. 119: The ten coins of this group (NH 363, NH 364, NH 379, NH 381, NH 383, NH 385, NH 386, NH 387, NH 389, NH 391; Table 1 and Table 3, Figure 1) share die-links. NH 363 and NH 364 were struck from the same pair of dies; NH 379, NH 381, NH 383, NH 385, NH 386, NH 387, NH 389, and NH 391 were struck from another pair of dies. Only well-preserved bright areas (according to BSE mode) were examined by EDS analysis.

SEM-EDS analysis results of the ten Cat. No. 119 coins revealed that they were composed of silver alloyed with copper; however, other elements were also detected (Electronic Supplementary Materials, Table S5). The measured average alloy composition, after omitting the peaks of O, Si, Cl, S, and Ca, was 97.6 ± 1.2 wt% Ag and 2.4 ± 1.2 wt% Cu (77 measurements of Cat. No. 119 coins were included in the calculations).

**Figure 7:**
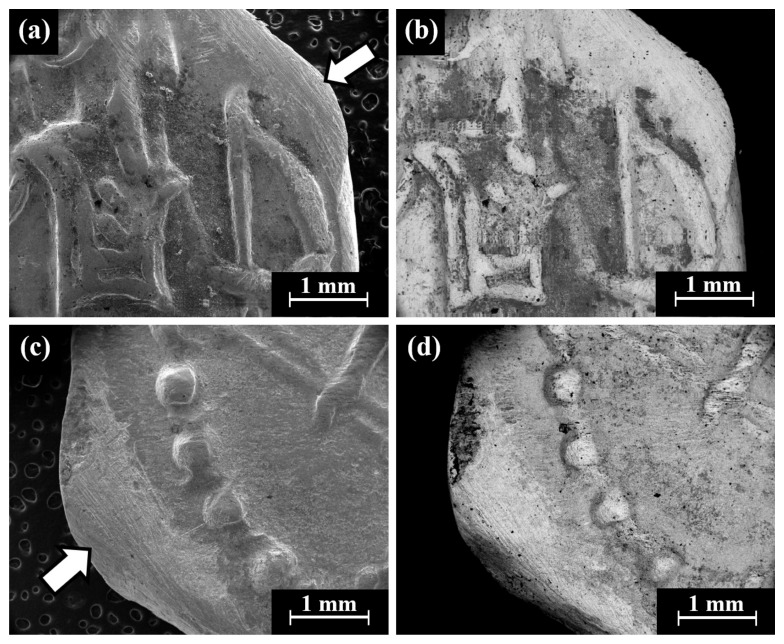
SEM images of the group pf coins of Cat. No. 164 (obverse): (**a**,**b**) coin IMJ 34778 (SE mode and BSE mode, respectively) and (**c**,**d**) coin IMJ 34877 obverse (SE mode and BSE mode, respectively), where the white arrow in Figure 4c shows the area that was ground.

Cat. No. 164: SEM observation of coins IMJ 34127, IMJ 34183, and IMJ 34809 (Table 1 and Table 3, Figure 1 and Figure 7a–d) shows the upper right side of the obverse (Figure 7a,b) and the lower left side of the reverse (Figure 7c,d). These areas were ground with abrasive paper in order to examine the composition of the bulk metal. Only well-preserved, bright areas (according to BSE mode) were included in the average silver alloy composition calculations (Figure 7b,d).

SEM-EDS analysis results of obverse and reverse surfaces of the three issues of Cat. No. 164 revealed that they were composed of silver alloyed with copper; however, other elements were also detected (Electronic Supplementary Materials, Table S6). The alloy of these coins after omitting the peaks of O, Si, Cl, and S revealed an average alloy composition value of 94.0 ± 2.7 wt% Ag and 6.0 ± 2.7 wt% Cu (18 measurements of the coins were included in the calculations).

Cat. No. 200: Coins of this group (NH 468, NH 469, NH 471, and NH 472; Table 1 and Table 3, Figure 1) share die links. NH 468 and NH 469 were struck from the same reverse; NH 471 and NH 472 were struck from the same pair of dies and from the same obverse as NH 468.

SEM-EDS analysis of the Cat. No. 200’s group revealed that the coins were composed of silver alloyed with Cu; however, other elements were also detected (Electronic Supplementary Materials, Table S8). The alloy of the coins belonging to this group, after omitting the peaks of O, Si, Cl, S, and Ca, revealed that the average alloy composition value of 97.1 ± 1.5 wt% Ag and 2.9 ± 1.5 wt% Cu (33 measurements of the coins were included in the calculations).

Cat. No. 295: This group of coins (NH 499, NH 501, NH 502; Table 1 and Table 3, Figure 1) share die-links. NH 499, NH 501, and NH 502 were struck from the same reverse (the obverse side was too worn in these specimens to determine whether they are die-linked). SEM-EDS analysis results of the coins belonging to Cat. No. 295 (Electronic Supplementary Materials, Table S11) revealed an average alloy composition value of 97.0 ± 1.6 wt% Ag and 3.0 ± 1.6 wt% Cu (20 measurements were included in the calculations after omitting the peaks of O, Si, Cl, S, and Ca).

Cat. No. 310: NH 560 and NH 565 were struck from the same pair of dies. SEM-EDS analysis of these two specimens (Table 1 and Table 3, Figure 1) revealed an average composition value of 97.3 ± 1.3 wt% Ag and 2.7 ± 1.3 wt% Cu (12 measurements were included in the average alloy composition calculations after omitting the peaks of O, Si, Cl, S, and Ca, Electronic Supplementary Materials, Table S12).

**Figure 8:**
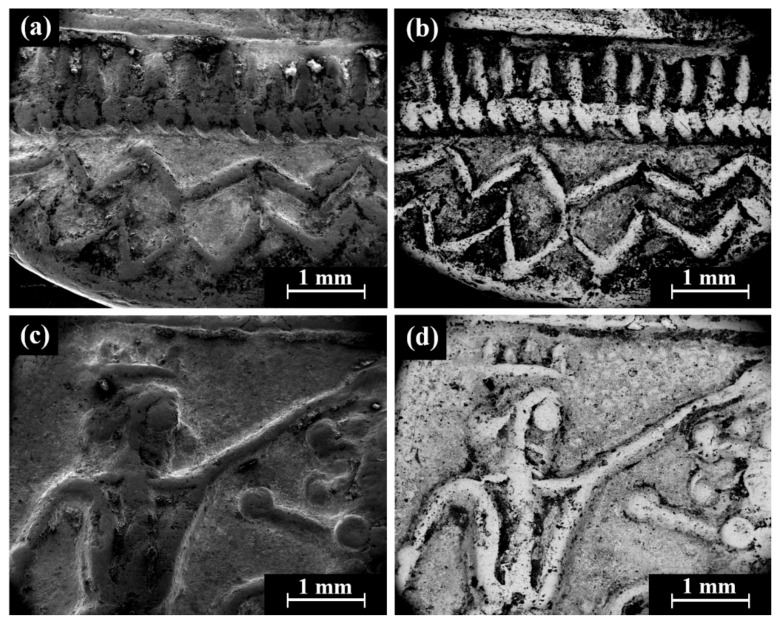
SEM images of the isolated coin IMJ 34487 (Cat. No. SID.ST. 35): (**a**,**b**) obverse side (SE mode and BSE mode, respectively) and (**c**,**d**) reverse side (SE mode and BSE mode, respectively).

Isolated coins (single examined issues, Table 1 and Table 3): SEM observation of the eight isolated coin types: Cat. No. 156/IMJ 34341, Cat. No. 168/NH 429 (NTWN), Cat. No. 169/NH 453 (ŠLWM), Cat. No. 178/IMJ 34197, Cat. No. 220/IMJ 34421, Cat. No. 297/NH 522, Cat. No. SID.ST. 35/IMJ 34487, and UNC. 3/IMJ 34503 (MQ IC-5 variant, early Hellenistic coin) showed various images on the obverse and reverse of the coins. For example, SEM observation of coin IMJ 34487 (Figure 8, Cat. No. SID.ST. 35) revealed an image of a war galley on the obverse (Figure 8a,b) and a figure of the Persian king or hero, fighting a lion on the reverse (Figure 8c,d).

SEM-EDS analysis results of obverse and reverse surfaces of the isolated Samarian silver alloy coins revealed they were composed of silver alloyed with copper; however, other elements were also detected, including O, Si, Cl, S, Na, Ca, Al, K, Mg, Fe, Pb, Sn, and Au (Electronic Supplementary Materials, Table S13). The presence of low gold content may indicate that non-argentiferous lead was employed to refine silver through cupellation [6].

Plated coins: SEM-EDS analysis results of five plated coins from four different groups of coins. (1) Cat. No. 18 coins IMJ 34344 and IMJ 34345, (2) Cat. No. 186/IMJ 34412, (3) Cat. No. 286/IMJ 34498, and (4) Cat. No. 222/IMJ 34424 revealed heterogeneity of the surface composition (as shown by the large standard deviation values) and high copper concentrations. For example, the SEM-EDS analysis results of the two plated coins belonging to Cat. No. 18 revealed an average composition of 44.5 ± 39.6 wt% Ag and 55.5 ± 39.6 wt% Cu (where 24 SEM-EDS measurements were included in the calculations). The results indicate that the plated coins were made of copper that was covered with thin silver foil.

### 3.2. Broken Pieces of Silver Ingots, Jewelry, Pieces of Scrap Silver (Hacksilber), and the Sidonian and Philistian Coins from the Hacksilber Hoard

VT inspection of the broken pieces of jewelry, silver ingots, and pieces of scrap silver from the hacksilber hoard [42] revealed that they were well preserved (Figure 2 and Figure 3, Table 2, and Electronic Supplementary Materials, Figure S1). The detailed SEM-EDS analysis results of each of the studied items are presented in the Electronic Supplementary Materials, Tables S14–S16. SEM images of these silver alloy items from the hoard revealed well-preserved bright areas according to BSE mode (Electronic Supplementary Materials, Figures S2–S4).

Since this research focuses mainly on the silver coins and hacksilber, most of the jewelry results are presented in the Electronic Supplementary Materials. However, a summary of the main findings related to the jewelry from the hacksilber hoard is provided below.

SEM-EDS analysis of the earring or ring (Sample 1, Electronic Supplementary Materials, Figures S1a and S2, and Table S14) revealed that it was made of a ternary silver–copper–gold alloy, with average alloy values of 95.1 ± 0.8 wt% Ag, 1.8 ± 0.6 wt% Cu, and 3.1 ± 0.2 wt% Au.

SEM-EDS analysis of the lunate earrings (Samples 2–7, Electronic Supplementary Materials, Figure S1b–g, Table S14) revealed that they were made of several alloys. For example, the average alloy composition of the lunate earrings (Samples 2–6) was 92.2 ± 6.9 wt% Ag, 4.2 ± 6.2 wt% Cu, and 3.6 ± 4.9 wt% Au, whereas analysis of the decorated lunate earring (Sample 7, Figure S1g) revealed that it was made of a ternary silver–copper–gold alloy, with average alloy composition of 76.0 wt% Ag, 1.4 wt% Cu, and 22.6 wt% Au. One of the lunate earrings (Sample 3, Figure S1d) revealed a yellowish metallic shiny appearance. SEM-EDS analysis results of this earring revealed that it was made of a ternary silver–copper–gold alloy with a composition of between 83.6 and 85.4 wt% Ag, 10.7–13.2 wt% Cu, and 3.2–3.9 wt% Au (Electronic Supplementary Materials, Table S14).

SEM-EDS analysis results of this granulated bead (Sample 8, Electronic Supplementary Materials, Figures S1h and S3, Table S14) revealed that it was made of pure silver (100 wt% Ag). SEM-EDS analysis of the cylindrical bead granules (Sample 9, Electronic Supplementary Materials, Figures S1i and S4, Table S14) revealed an average alloy composition of 99.7 ± 0.6 wt% Ag and 0.3 ± 0.6 wt% Cu (Table 5). Although the granules and the brazing material were made of high-purity silver, slightly higher concentrations of copper were measured within the joints than in the surrounding parts, which made it possible to join the granules together without melting them.

SEM-EDS analysis of the circular/spiral bead (Sample 10, Electronic Supplementary Materials, Figure S1j, Table S14) revealed an average alloy composition of 90.8 ± 5.8 wt% Ag, 8.0 ± 5.8 wt% Cu, and 1.2 ± 1.3 wt% Au. SEM images of a rings with a decorated oval front (Samples 11 and 12) show the fracture surface at the broken back of each ring (Electronic Supplementary Materials, Figure S5 and Figure S6, respectively). SEM-EDS analysis of the three decorated rings (Samples 11–13, Supplementary Materials, Figures S5 and S6) showed that they were produced from ternary silver–copper–gold alloy, with an average alloy composition of 92.0 ± 2.3 wt% Ag, 3.5 ± 1.2 wt% Cu, and 4.5 ± 2.6 wt% Au.

SEM-EDS analysis of the broken plain ring fragments (Sample 14, Supplementary Materials, Figure S1n, Table S14) revealed a ternary silver–copper–gold alloy with an average alloy composition of 95.9 ± 2.6 wt% Ag, 1.2 ± 1.7 wt% Cu, and 2.9 ± 2.1 wt% Au.

VT inspection of the eight silver sheets (samples 15–22, Figure 3a–h) revealed that they were covered with dark oxide, corrosion products, and soil elements. SEM-EDS analysis of the flat silver sheets revealed that their surface was composed of Ag, Cu, and Au, yet other elements were also detected, including O, C, Si, Cl, Al, S, and Ca (Electronic Supplementary Materials, Table S15). EDS analysis of Samples 15–22, after omitting the peaks of oxides, corrosion products, and soil elements, revealed that the sheets were produced of various alloys, including pure silver, binary silver–copper, and ternary silver–copper–gold alloys (Electronic Supplementary Materials, Table S15), with the average composition of all samples being 95.2 ± 5.5 wt% Ag, 3.2 ± 5.8 wt% Cu, and 1.6 ± 1.7 wt% Au.

**Figure 9:**
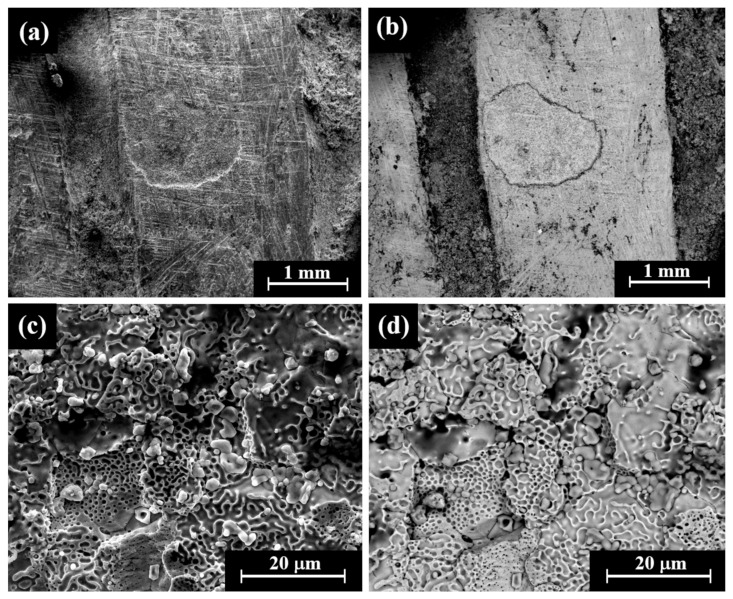
SEM images of a striated ingot from the hacksilber hoard (Sample 23): (**a**,**b**) general view of the surface (SE mode and BSE mode, respectively) and (**c**,**d**) higher magnification of the surface (SE mode and BSE mode, respectively), where the bright areas according to BSE mode were examined by SEM-EDS analysis.

VT of a perhaps striated(?) ingot (Sample 23, Figure 3i) revealed that although the item was covered with dark oxide, it was a well-preserved piece. The item was examined by SEM SE and BSE modes (Figure 9). A dendritic microstructure was observed (Figure 9c,d), which indicated that this ingot was an as-cast object. The development of porosity (Figure 9c,d) may show a selective dissolution (dealloying) of copper in the alloy. The corrosion of binary Ag-Cu alloys, depending on exposure conditions, often leads to the dealloying when exposed to certain environments due to the electrochemical differences between silver and copper and their reaction with the surrounding environment. The selective dissolution of copper leads to the formation of an Ag-rich sponge microstructure, which may either remain as a stable protective layer (surface passivation) or breakdown, depending on the environmental conditions [49]. EDS analysis revealed that this piece was produced of a ternary silver–copper–gold alloy, yet presence of the elements O, Si, and Cl was also detected (Electronic Supplementary Materials, Table S15). The average alloy composition of the striated ingot after omitting the peaks of oxides, corrosion products, and soil elements was 97.5 ± 1.0 wt% Ag, 0.4 ± 0.6 wt% Cu, and 2.1 ± 0.4 wt% Au.

**Figure 10:**
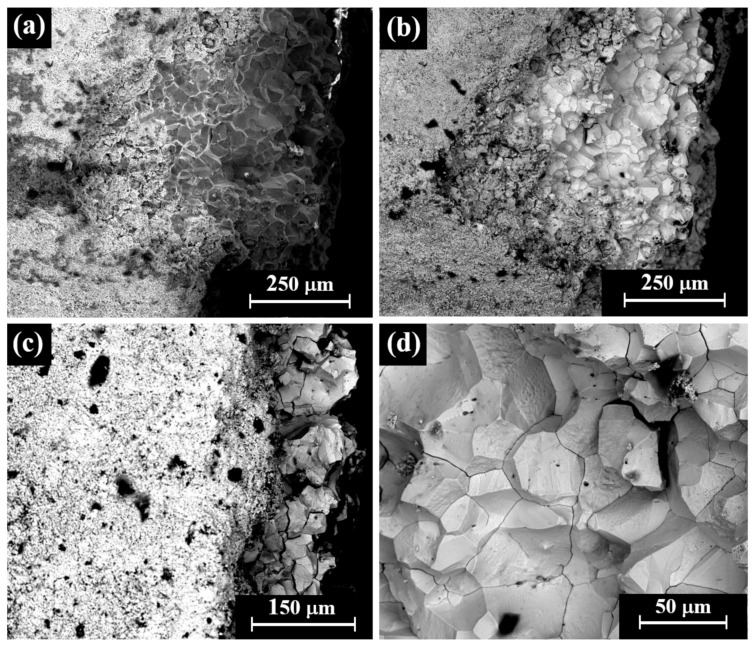
SEM images of a Sidonian coin from the hacksilber hoard (Sample 24, reverse): (**a**,**b**) general view of the coin showing the fracture surface (area 1, SE mode and BSE mode, respectively), (**c**) general view of the coin showing the fracture surface (area 2, BSE mode), and (**d**) higher magnification of the fracture surface (BSE mode).

VT of the two issues, Sample 24 and Sample 25 (Sidonian coin and Philistian coin, Figure 3j, Figure 3k, respectively), revealed that these coins were covered with dark oxide and some corrosion products. SEM observation revealed that the surfaces of the Sidonian and Philistian coins were covered with dark areas (according to BSE mode), yet some bright areas were also observed on the coins’ surfaces (Figure 10). SEM observation of the Sidonian coin (Sample 24) exposed a mixed intergranular and transgranular fracture surface (Figure 10).

EDS analysis of the two coins from the hacksilber hoard revealed that they were made of Ag-Cu alloy; however, other elements were also detected including Si, O, Cl, Na, Al, Ca, and S (Electronic Supplementary Materials, Table S16). The average composition of the alloy of the two was 99.7 ± 0.5 wt% Ag and 0.3 ± 0.5 wt% Cu (after omitting the peaks of Si, O, Cl, Na, Al, Ca, and S). For a comparison, the copper content detected in the Yehud coinage is normally about 3 wt% [2].

The average silver alloy composition of all the 25 items from the late-fifth-century BCE hacksilber hoard, including the various pieces of jewelry, cut silver sheets (hacksilber), one striated ingot, and two coins, are presented in Table 5, showing the variety of alloys that were used to produce the items.

## 4. Discussion

In the current study, 35 groups of Samarian fourth-century BCE silver coins from public collections (mainly from the IMJ), as well as hacksilber, jewelry, and coins from hoards from the Samaria region were studied by NDT analyses. The items were studied from typological and archeometallurgical perspectives. The aims of the research were to characterize the composition of the Persian-period Samarian silver coins, and to compare the different groups of coins.

The methodology for studying the Samarian silver coins and silver sheets included four steps: (1) VT to assess preservation and gain a deeper understanding of the manufacturing methods; (2) SEM observations in SE and BSE modes to evaluate surface preservation; (3) SEM-EDS analysis to compare surface composition with the bulk, using locally ground surfaces for comparison; and (4) EDS analysis of bright areas in BSE mode to calculate the average alloy composition, excluding peaks of oxides, corrosion products, and soil elements.

The SEM-EDS analysis of the surfaces of the Samaria coins from public collections revealed that they were primarily made of silver with small wt% of Cu, along with traces of O, Si, Cl, S, Na, Ca, Al, K, P, Mg, Fe, Zn, Sn, Pb, and Au. The presence of Cl, Na, O, and Si may suggest that the coins were subjected to soil salinity during their burial period, or were exposed to a saline environment during their cleaning process. Unfortunately, there is no documentation related to the specific environment in which the coins were discovered nor information concerning the cleaning methods of the coins.

The high silver values on the surface of the Samarian silver coins could be due to a silver surface enrichment process, which results from the segregation phenomenon of the silver and copper in the alloy. Over time, the corroded copper compounds are removed from the surface due to harsh environmental conditions. This process leaves a surface that is enriched in silver, as silver does not corrode as easily as copper [21]. However, since no significant differences were found between the composition on the surface of the bright areas (according to BSE mode) and the composition after grinding, no evidence was found of segregation and silver surface enrichment.

Ancient silver, often produced from galena ore through the cupellation method, was a high-purity silver metal [3,50]. The absence of lead in most of the examined areas of the coins probably indicates an effective cupellation refining process. Yet, in some cases, silver items were produced from ores other than galena and hence the silver produced could have a very low impurity content of lead and gold below the limits of detection. The detection of chlorine and sulfur aligns with silver’s reactivity to chloride and sulfide ions, forming silver chloride (AgCl) and sulfide (Ag_2_S) compounds [15,51]. Elements such as O, Cl, Si, S, P, Al, Ca, Fe, and Mg are likely associated with the presence of corrosion products and soil remains [15,16,25].

For more representative SEM-EDS analysis of the coins’ bulk material, calibrating results against a few standard metallographic cross-sections is recommended. This approach provides better insights into the homogeneity of composition and microstructure, including the extant of metal working and annealing. Since destructive metallographic testing was not feasible in this study, localized grinding of different areas on a few coins was performed to expose metallic surfaces. Additionally, the fracture surface of a Sidonian coin from the hacksilber hoard was examined. It revealed a mixed intergranular and transgranular fracture surface (Figure 10), where cracks propagated through and along the material grains, likely due to prolonged corrosion processes [49,52,53,54]. SEM fractography of Sample 11 (ring) from the hacksilber hoard (Electronic Supplementary Materials, Figure S5) showed an intergranular fracture with cracks propagating along the grain boundaries. Similarly, SEM fractography of Sample 12 (ring) from the hacksilber hoard (Electronic Supplementary Materials, Figure S6) revealed a mixed fracture of a brittle transgranular and intergranular embrittlement, where the cracks propagated through the material grains and along the grains, respectively, probably resulting from long-time corrosion processes [48,49,52,53,54].

Copper was a preferred alloying element in the manufacture of ancient Palestinian/southern Levantine coins and jewelry [13]. High-purity silver coins suggest that the minting process of these items was not significantly influenced by economic constraints [20]. Certain results (e.g., Cat. No. 178) displayed higher concentrations of copper in the alloy, suggesting a deliberate addition of copper, but this cannot be used for designation of these coins to a specific mint.

Despite the excellent environmental resistance of silver, ancient silver objects buried for a long period, tend to be covered with different oxides and corrosion products, such as grayish silver chloride (AgCl), black silver sulfide (Ag_2_S), dark-brown to black Silver(I) oxide (Ag_2_O), black silver(II) oxide (AgO), yellowish or light-brown silver carbonate (Ag_2_CO_3_), green malachite [Cu_2_CO_3_(OH)_2_] copper carbonate, blue azurite [Cu_3_(CO_3_)_2_(OH)_2_] copper carbonate, black or dark-brown copper sulfides (Cu_2_S, CuS), green copper sulfates [Brochantite, Cu_4_SO_4_(OH)_6_], red copper oxide (Cu_2_O), and black copper oxide (CuO) [45,46,47,48]. Therefore, only well-preserved shiny areas (bright areas according to SEM BSE mode) were examined by SEM-EDS analysis in order to calculate the average alloy composition. In addition, the surface of representative silver items was ground until a shiny silver metal was exposed in order to remove oxides and corrosion products. Based on the current results, it is concluded that when the coins are made of relatively pure silver and are well preserved, their surface reflects their bulk composition quite well.

To ensure reliable SEM-EDS analysis results that accurately reflect the bulk material of the examined silver objects, only bright regions of well-preserved silver metal, as identified in BSE mode, were selected for calculating average composition value. The average alloy composition of the different groups of coins from Samaria according to SEM-EDS analysis, after eliminating the peaks of oxides and soil elements (Table 3 and Table 4), was between 100 wt% Ag (Cat. Nos. 156 and 220) and Ag with 10.7 wt% Cu (Cat. No. 178), where each group had its own average composition. SEM-EDS analysis of the die-linked coins belonging to Cat. No. 117 (NH 343, NH 345, NH 346, NH 347, and NH 348) revealed an average alloy composition of 94.1 ± 4.5 wt% Ag and 5.9 ± 4.5 wt% Cu. The other issues belonging to Cat. No. 117 (NH 340, NH 352, NH 355, NH 358) revealed an average alloy composition of 94.7 ± 2.4 wt% Ag and 5.3 ± 2.4 wt% Cu, where the average composition of all nine issues belonging to Cat. No. 117 was found to be 94.4 ± 3.7 wt% Ag and 5.6 ± 3.7 wt% Cu (74 EDS measurements were included in the calculations). Based on average alloy composition and standard deviation values of all the issues of Cat. No. 117, all nine coins were produced by the same controlled specific composition of silver–copper alloy. The average copper concentration detected in all the issues of Cat. No. 117 was 5.6 wt% Cu; therefore, it is proposed that the silver blank used to mint these issues was deliberately alloyed with copper, probably to improve the mechanical properties and to reduce the melting point of the alloy [10,11,15,16]. Based on these results, it is almost certain that all issues of Cat. No. 117 were produced from similar raw materials and manufactured in the same workshop.

The alloy of the die-linked coins belonging to Cat. No. 119 (NH 379, NH 381, NH 383, NH 385, NH 386, NH 387, NH 389, NH 391) revealed an average alloy composition value of 97.5 ± 1.3 wt% Ag and 2.5 ± 1.3 wt% Cu. Two additional coins from Cat. No. 119 (NH 363 and NH 364) revealed an average composition of 98.1 ± 0.7 wt% Ag and 1.9 ± 0.7 wt% Cu (derived from 16 EDS measurements of their obverse and reverse sides; however, two measurements from the reverse of coin NH 364, areas 4 and 5, were excluded from the calculations due to the presence of dark corrosion products). The average alloy composition of all the issues of Cat. No. 119 was found to be 97.6 ±1.2 wt% Ag and 2.4 ± 1.2 wt% Cu. Therefore, it is almost certain that all the coins of Cat. No. 119 were produced from the same batch of silver bullion and were produced in the same workshop.

The two issues of Cat. No. 200 were found to be die-linked. These two coins revealed an average alloy composition of 97.1 ± 1.5 wt% Ag and 2.9 ± 1.5 wt% Cu. Therefore, the two issues of Cat. No. 200 were produced by a controlled manufacturing processes and it is almost certain that these die-linked coins were produced from the same controlled raw material and originated in the same workshop.

All three coins of Cat. No. 295 were found to be die-linked. These die-linked issues of Cat. No. 295 revealed an average composition of 97.1 ± 1.5 wt% Ag and 2.9 ± 1.5 wt% Cu (three measurements of coin 502’s reverse, areas 3, 5, and 6 and one measurement of coin 502’s obverse, area 5, were not included in the calculations of the average composition since these areas were corroded). Based on the average alloy composition, all issues of Cat. No. 295 were produced by a controlled manufacturing process. Moreover, it is almost certain that these issues were produced from the same batch of silver bullion and originated in the same workshop.

Based on the current research results, silver metallurgy was a well-established practice in the southern Levant during the Persian (Achaemenid) period. The coins that belong to Cat. No. 119, Cat. No. 200, and Cat. No. 295 have similar average alloy compositions produced of high-purity silver with an average copper content of between 2.4 and 2.9 wt% Cu. Therefore, it is possible that the coins belonging to these three groups were produced from the same batch of silver bullion and originated in the same workshop and were issued by the same mint/workshop. The current results suggest that during the Persian period there were a limited number of workshops in the Samaria region that produced silver coins in a controlled and authorized manner.

The average composition of the plated samples varied between 3.8 ± 2.6 wt% Ag and 96.2 ± 2.6 wt% Cu (for Cat. No. 222) and 51.8 ± 22.8 wt% Ag and 48.2 ± 22.8 wt% Cu (for Cat. No. 186, Supplementary Materials, Table S7), which indicated that the thin silver coating of the plated coins was eroded through time, as also observed by VT examination.

Based on the average silver alloy composition values of the Samarian coins (Table 4), it can be determined that these coins were produced by a controlled composition of silver–copper alloy. Considering the finely detailed iconographic motifs that appear on the obverse and reverse surfaces of the coins, the preparation of the dies required remarkable technological and artistic skills. The manufacturing process of the Samarian silver coins is the same as that of the Yehud coins [2], where both were produced of binary silver–copper alloys with a small wt% of copper. Yet, the distribution of the composition is greater, and the copper concentration reaches slightly higher values in the Samarian coins. In the Yehud coins, around 10% of the items had an exceptional composition with a higher concentration of copper presented in the silver alloy [2], whereas in the Samarian coins five of the issues (Cat. No. 18, Cat. No. 186, Cat. No. 286, and Cat. No. 222) were created of copper alloy blanks that were wrapped with thin silver foil and then each copper blank was plated with silver during the stamping procedure of the coin.

The Samarian coin composition (Table 4) is similar to the examined jewelry from the SH and NH (the average copper concentration in the base metal of the jewelry from the SH and the NH was 5.8 ± 3.0 wt% Cu and 5.0 ± 3.2 wt% Cu, respectively), where both types of artifacts were made of silver alloy containing a small percentage of copper. However, in the case of the jewelry from the SH and NH, higher concentrations of copper were detected in the joints, with an average concentration of 17.0 ± 10.0 wt% Cu in both hoards [15,16,33].

SEM-EDS analysis results of the silver jewelry, coins, and sheets belonging to the hacksilber hoard revealed they were made of varied alloys (Table 5). The large earring/ring (Sample No. 1) was made of a ternary silver–copper–gold alloy; the other lunate earrings (Sample Nos. 2–7) were made of a binary silver-gold, binary silver–copper, and a ternary silver–copper–gold alloys; and the granulated beads (Samples Nos. 8–9) were made of almost pure silver with only a very small percentage of copper. The circular/spiral bead (Sample No. 10), three rings with oval bezels (Sample Nos. 11–13), and the plain broken ring (Sample No. 14) were all made of a ternary silver–copper–gold alloy [42]. The nine silver/ingot fragments (Samples 15–22) were made of diverse alloys, including pure silver, binary silver-gold, and ternary silver–copper–gold alloys, whereas the striated ingot (Sample 23) was made of a ternary silver–copper–gold alloy. The Sidonian and Philistian coins from the hacksilber hoard examined here (Sample Nos. 24–25) were made of silver alloy containing a small percentage of copper, with a relatively similar composition to the collection of the Samarian silver coins and to the silver coins from the SH and NH [15,16].

The EDS analysis revealed that the jewelry and silver sheets from the hacksilber hoard were produced of pure silver, binary silver–copper alloy, binary silver-gold alloy, and ternary silver–copper–gold alloy (Table 5 and Electronic Supplementary Materials, Tables S14–S16). The Sidonian and Philistian coins were found to be made of binary silver alloy containing a small percentage of copper (Table 5 and Electronic Supplementary Materials, Table S16). The manufacturing processes of the hacksilber hoard jewelry included casting, sawing, hammering, twisting, shaping by plastic deformation, granulating, as well as joining methods. The manufacturing processes of the jewelry from the SH and NH [15,16] were found to be similar to the methods used to produce the jewelry in the hacksilber hoard. A noted continuity in the regional artistic styles and production technologies was observed in the silver coins and the silver jewelry. This information provides a better understanding of the local material culture and technological skills in the southern Levant and specifically in the province of Samaria through the Persian period.

The SEM-EDS results revealed that in the case of items with a high wt% of silver and only low wt% of copper, the copper wt% concentration in the bulk of the coins after grinding the surface was similar to the well-preserved external surfaces of the items. Yet, for items with high wt% of copper the surface was significantly different from the bulk (base metal) composition, for example, in the case of the plated coins; therefore, the methodology developed is not suitable for silver items with an average copper composition of more than 10 wt% Cu.

The VT and SEM observations indicated that the Samarian silver coins from public collections as well as the Sidonian and Philistian silver coins were produced using cast flans. Some of the Samarian coins in Figure 1 have angular cut marks. This indicates that they were cast into a mold with concavities, either in the form of a strip or in the form of a tree, which would then separate into isolated flans. Assuming that it was desired to smooth the surface, remove fire stain, and to produce bright and shiny silver coins, the flans were most probably polished before they were struck. The flans were then heated and later placed between obverse and reverse dies (made of high tin bronze alloy) that were struck together with a hammer or a press, imprinting the details on both sides of the silver flans [2,28].

The artistic style of the jewelry of all three hoards allegedly found in the area of Samaria [15,16,42], which involved elaborate workmanship (the current hacksilber hoard as well as the SH, and NH), was similar. In addition, the manufacturing processes of the silver jewelry from all three hoards were similar. For example, the earring with granules from the current hoard (Sample No. 7, Electronic Supplementary Materials, Figure S1g) looks like the decorated earring from the NH (earing B) [15]. The twisted coil/wire element decoration appears in the large earring/ring (Sample No. 1, Electronic Supplementary Materials Figure S1a) from the current hacksilber hoard and the decorated earring from the NH (earring A). The granulated beads (Sample Nos. 8–9, Electronic Supplementary Materials Figures S1h,i, S3 and S4) are similar in style to the granulated beads from the NH and the SH hoards [15].

A notable continuity in artistic styles and production technologies was observed in the coins and jewelry, indicating a shared regional material culture and technological expertise in the southern Levant, specifically in the Persian-period province of Samaria. Our study therefore provides further information on the advanced technological skills and artistic level in the province of Samaria and in the southern Levantine region during the Persian period.

## 5. Conclusions

This study provided a comprehensive characterization of 35 groups of fourth-century BCE Samarian silver coin types from public collections, along with silver cut/ingot (hacksilber) and two coins from the late-fifth-century BCE hacksilber hoard from the region of Samaria. Using non-destructive techniques, the items were analyzed from typological and archeometallurgical perspectives to determine their composition and manufacturing processes. The primary objectives were to characterize the composition of the objects, compare the different groups of coins, and analyze the hacksilber hoard’s silver alloy. The methodology included visual test (VT) examination, SEM observation, and SEM-EDS analysis, with a focus on well-preserved metallic silver areas for accurate bulk material representation.

The SEM-EDS analysis revealed that the Samarian coins were primarily made of a binary silver–copper alloy, with a controlled composition of silver containing a small percentage of copper. The intricate iconographic motifs and inscriptions on the obverse and reverse of the coins indicated that their production required advanced technological and artistic skills. The SEM-EDS results confirmed that for high-silver content items with low copper percentages, the bulk and surface compositions were similar. However, items with higher copper content showed significant surface-to-bulk composition differences, indicating the methodology’s limitations for silver items with high copper content.

The elemental analysis of the silver sheets and jewelry from the hoard demonstrated the use of various materials, including pure silver, binary silver–copper alloy, and ternary silver–copper–gold alloy. The Sidonian and Philistian coins from the hoard were also made of a binary silver–copper alloy.

The manufacturing processes of the jewelry from the hacksilber hoard [42] included techniques like those used in the SH and NH [15,16]. A notable continuity in artistic styles and production technologies was observed in the coins and jewelry, indicating a shared regional material culture and technological expertise in the southern Levant, specifically in the Persian-period province of Samaria.

In conclusion, this study provides valuable insights into the technological skills and artistic levels in the province of Samaria and the southern Levant during the Persian period. The detailed analysis of the silver coins and hacksilber items highlights the advanced metallurgical and artistic capabilities of the region, contributing to our understanding of ancient material culture and production technologies.

## Figures and Tables

**Table 1:** Obverse and reverse descriptions of the analyzed Samaria coins.

Coin Type According to the New Typological Division of Samarian Coins	Obverse Description	Reverse Description
Cat. No. 1	Forepart of crouching lion to right (r.)	Bearded male head (Heracles?) to left (l.); in r. field, ŠMRYN–שמרינ (in Aramaic)
Cat. No. 18	Satrap(?) head wearing a Persian headdress to r.	Horse forepart to r.; in l. field, ŠMRYN–שמרינ (in Aramaic)
Cat. No. 21	War-galley to l.; below, two lines of waves; above, ŠMRYN–שמרינ (in Aramaic)	Persian king/hero facing a lion; between them, MZ–מז (in Aramaic)
Cat. No. 22	Charioteer or satrap in horse-drawn chariot to r., reins and a sword; behind, a figure wearing a crown and raising his hand	Persian king/hero facing a lion; between them, MZ–מז (in Aramaic)
Cat. No. 23	Persian king(?) seated on throne to r. holding a flower and a scepter; in l. field, ŠN–שנ (in Aramaic)	Ahura Mazda to r. holding a flower and a ring(?) in the other; in l. field, MZ–מז (in Aramaic)
Cat. No. 39	Male head wearing a headdress to l.; in r. field, ΦAΡΝΒAΖC (in mixed Aramaic and Greek script)	Winged horse (Pegasus or hippocamp); forepart to r.; below, NRMŠ–נרמש (in Aramaic, retrograde)
Cat. No. 48	Helmeted head of Athena to l.	Lion attacking stag to r.; in l. field, ŠMRYN–שמרינ (in Aramaic)
Cat. No. 104	Horse handler to l.; in l. field, ŠL–של (in Aramaic)	Lion leaping to l.; below, the lion, ram’s head to r.; in upper r. field, LŠ–לש (in Aramaic)
Cat. No. 109	Lion seated to r.; in upper l. field, ŠL –של (in Aramaic)	Lion seated to r.; in upper l. field, ŠL–של (in Aramaic)
Cat. No. 112	Helmeted head of Athena to r.	Owl standing facing with spread wings; Š–ש, L–ל (in Aramaic) on both sides of the wings
Cat. No. 117	Satrap(?) wearing a Persian headdress; seated on diphros to r., checking an arrow; bow resting below; in upper r. field, BT–בת (in Aramaic); in l. field, numeral 14 (in Aramaic)	Persian king/hero facing a bull; pellet(?) between them; in r. field, ΒA/ΓAΒAP/ΤAC (in Greek)
Cat. No. 119	Persian king(?) wearing a crown, seated on throne to r., smelling a flower and holding a scepter; incense burner in front; in upper r. field, BT–בת (in Aramaic)	Persian king/hero standing on l., facing a bull; pellet(?) between them
Cat. No. 134	Helmeted head of Athena to r.	Owl standing; in upper l. field olive spray and crescent; in r. field, AΘE; ‘BD’L–עבדאל (in Aramaic)
Cat. No. 138	Helmeted head of Athena to r.	Owl standing; in r. field, AΘE; in upper l. field, olive spray; ‘BD’L–עבדאל (in Aramaic)
Cat. No. 154	Hero(?) holding a spear standing next to a bull or a lion	Male head (Heracles) to l.; in l. field, Y(?)RB‘M–ירבעמ (in Aramaic)
Cat. No. 155	Female head (Arethusa?) facing	Bearded satrap(?) head to r.; in l. field, WNY–וני or D/RNY–ד/רני (in Aramaic)
Cat. No. 156 (isolated)	Female head (Arethusa?) facing	Bearded satrap(?) head to r.
Cat. No. 157	Female head (Arethusa?) facing	Bearded satrap(?) head to r.
Cat. No. 158	Persian king or Zeus seated on throne; in upper r. field, IEYΣ (in Greek); incense burner (thymiaterion) in front	Satrap(?) rider on horseback to r.; below, YHW‘NH–יהוענה (in Aramaic)
Cat. No. 162	Persian king(?) wearing a crown, seated on throne to r., smelling a flower and holding a scepter	Persian king(?) wearing a crown standing to r., raising hand and holding a scepter; in l. field, BRWḤBL–ברוחבל (in Aramaic)
Cat. No. 163	Charioteer or satrap in horse drawn chariot to l., holding reins and a sword; behind the charioteer, a figure wearing a Persian headdress and raising his hand	Satrap(?)riding horse to l., holding reins of the horse and a sword; below, BRWḤBL–ברוחבל (in Aramaic)
Cat. No. 164	Persian king/hero as archer standing to r.; holding arrows and a bow; quiver on his back	Bes standing facing; in l. field, BRWḤBL–ברוחבל (in Aramaic)
Cat. No. 168 (isolated)	Helmeted head of Athena to r.(?)	Human (bearded male) headed bird (Ba?) to r.; in r. field NTWN–נתונ (in Aramaic)
Cat. No. 169 (isolated)	Helmeted head of Athena to r.	Owl standing; in r. field AΘE; in l. field, ŠLWM–שלומ (in Aramaic)
Cat. No. 178 (isolated)	Helmeted head of Athena to r.	Owl standing; in r. field AΘE; an ear of wheat; YDW‘–ידוע (in Aramaic)
Cat. No. 181	Helmeted head of Athena to r.	Owl standing; in upper l. field, olive spray and crescent; in r. field, ŠHRW–שהרו (in Aramaic)
Cat. No. 183	Helmeted head of Athena to r.	Female head facing; in upper r. field, Y–י (in Aramaic or paleo-Hebrew)
Cat. No. 186	Horned lion or lynx head to r.	Bull forepart to r.
Cat. No. 200	Two male figures standing facing each other (adoration scene); between them, an incense burner	Griffin attacking stag to r.
Cat. No. 220 (isolated)	Lion head facing	Female head (Arethusa?) facing
Cat. No. 221	Lion head facing	Female head facing
Cat. No. 231	Lion head facing	Bull forepart to r.; in upper field–עע (in Aramaic) or two circles
Cat. No. 246	Winged mythical animal (horned lion or griffin) seated to r.;	Crouched lion to l.; eating the hindleg/thigh bone of its prey
Cat. No. 286	Gorgoneion head facing	Owl standing
Cat. No. 295	Helmeted head of Athena to r.	Owl standing
Cat. No. 297 (isolated)	Helmeted head of Athena to r.	Owl standing; in upper l. field, olive spray and crescent; in r. field, AΘE
Cat. No. 299	Dome shaped	Owl standing
Cat. No. 309	Helmeted head of Athena to r.	Owl standing
Cat. No. 310	Helmeted head of Athena to r.	Owl standing
Cat. No. SID.ST. 35 (isolated)	War galley to l.; above B–ב (in Aramaic)	Persian king/hero facing a lion; between them, a circle or possibly Phoenician ‘–ע; dotted square border
UNC. 3 (isolated)	Female head wearing a headdress or flat crown to r.	Eagle standing to r., over thunderbolt; in r. field, club

**Table 2:** Description and dimensions of the items from the hoard. Items which were cleaned before SEM-EDS analysis were weighed before and after cleaning.

Item	Description	Dimensions and Weight
Ornamented ring (or earring), Sample 1	Cylindrical cast with twisted coil/wire element	External diameter: 30 mm, internal diameter 22 mm; 11.26 g
Ornamented earring, Sample 2	Lunate earring	25 mm length, 18 mm width; 5.66 g (before cleaning), 5.31 g (after cleaning)
Ornamented earring, Sample 3	Broken lunate earring	16 mm length, 15 mm width; 3.05 g (before and after cleaning)
Ornamented earring, Sample 4	Broken lunate earring	15 mm length, 20 mm width; 3.19 g (before cleaning), 3.18 g (after cleaning)
Ornamented earring, Sample 5	Broken lunate earring	27 mm length, 20 mm width; 6.25 g (before cleaning), 6.24 g (after cleaning)
Ornamented earring, Sample 6	Broken lunate earring	20 mm length, 17 mm width; 5.99 g (before cleaning), 5.97 g (after cleaning)
Granulated earring, Sample 7	Broken lunate earring with attached clusters of granules	27 mm length, 15 mm width; 4.26 g (before cleaning), 4.24 g (after cleaning)
Bead, Sample 8	Two rings of seven granules each, originally adorned with seven granules on each ring	Diameter: 8 mm; 0.62 g (before cleaning), 0.60 g (after cleaning)
Bead, Sample 9	Cylindrical with edges adorned with six granules each	Diameter: 4 mm, l.6 mm; 0.34 g (before cleaning), 0.33 g (after cleaning)
Bead, Sample 10	Circular/spiral	Diameter of 10 mm; 1.09 g
Ring, Sample 11	Broken ring with wide oval-shaped bezel with geometric design	Diameter (ring): 20 mm, bezel length 25 mm, width 19 mm; 6.57 g (before cleaning) 5.73 g (after cleaning)
Ring, Sample 12	Broken ring with oval-shaped bezel with geometric design	Diameter: 11 mm, bezel 14 mm width; 11 mm length; 2.37 g (before cleaning) 2.26 g (after cleaning)
Ring, Sample 13	Broken ring with oval-shaped bezel with geometric design	Bezel: 13 mm width, 14 mm length; 2.50 g (before cleaning), 2.18 g (after cleaning)
Ring, Sample 14a–d	Broken ring (4 fragments)	Diameter of 22 mm, 5 mm width; 2.82 g (combined)
Flat fragments, Sample 15	Rectangular-shaped sheet	21 mm length, 16 mm width, 0.2–0.4 mm thickness; 0.64 g
Flat fragments, Sample 16	Rectangular-shaped sheet	22 mm length, 19 mm width, 0.2–0.4 mm thickness; 0.83 g
Flat fragments, Sample 17	Rectangular-shaped sheet	23 mm length, 15 mm width, 0.2–0.4 mm thickness; 0.65 g
Flat fragments, Sample 18	Rectangular-shaped sheet	20 mm length, 13 mm width, 0.2–0.4 mm thickness; 0.50 g
Flat fragments, Sample 19	Rectangular-shaped sheet	20 mm length, 16 mm width, 0.2–0.4 mm thickness; 0.58 g
Flat fragments, Sample 20	Rectangular-shaped sheet	22 mm length, 20 mm width, 0.2–0.4 mm thickness; 0.78 g
Flat fragments, Sample 21	Rectangular-shaped folded sheet	9 mm length, 9 mm width, 0.2–0.4 mm thickness; 0.83 g
Flat fragments, Sample 22	Rectangular-shaped sheet	18 mm length, 16 mm width, 0.2–0.4 mm thickness; 0.59 g; (before cleaning), 0.51 g (after cleaning)
Block-shaped striated ingot(?), Sample 23	Differentiate between “proper” hacksilber, which is cut from ingots, and “secondary” hacksilber made from broken precious metal “finished” items.	20 mm length, 16 mm width, 2.5 mm thickness; 6.75 g (before cleaning), 6.45 g (after cleaning)
Sidonian coin, Sample 24	Sidonian 1/16 šql, ca. 425–402 BCE	Diameter: 11 mm; 0.67 g
Philistian coin, Sample 25	Philistian/Southern Levantine Athenian-styled *m*‘*h*, late fifth century BCE	Diameter: 0.9 mm; 0.60 g

**Table 3:** The average alloy composition (wt% content) of each coin from public collections according to SEM-EDS analysis after omitting the peaks of oxides, corrosion products, and soil elements. Plated coins were not included in the average alloy composition calculations.

Sample	Group	No. of EDS Measurements	Average Silver Content in the Alloy (wt% Ag)	Average Copper Content in the Alloy (wt% Cu)
IMJ 34127	Cat. No. 1	6	92.0 ± 1.1	8.0 ± 1.1
IMJ 34183	Cat. No. 1	11	98.4 ± 2.1	1.6 ± 2.1
IMJ 34809	Cat. No. 1	7	94.5 ± 3.3	5.5 ± 3.3
IMJ 34184	Cat. No. 18	5	100	–
IMJ 34343	Cat. No. 18	12	97.7 ± 1.8	2.3 ± 1.8
IMJ 34803	Cat. No. 18	6	100	–
IMJ 34807	Cat. No. 112	6	99.5 ± 0.7	0.5 ± 0.7
IMJ 34808	Cat. No. 112	6	100	–
IMJ 34353	Cat. No. 112	5	97.2 ± 4.1	2.8 ± 4.1
IMJ 34354	Cat. No. 112	6	98.5 ± 0.6	1.5 ± 0.6
NH 340	Cat. No. 117	8	97.2 ± 1.1	2.8 ± 1.1
NH 343	Cat. No. 117	10	89.4 ± 6.1	10.6 ± 6.1
NH 345	Cat. No. 117	7	94.1 ± 0.8	5.9 ± 0.8
NH 346	Cat. No. 117	7	94.6 ± 1.2	5.4 ± 1.2
NH 347	Cat. No. 117	6	97.0 ± 2.1	3.0 ± 2.1
NH 348	Cat. No. 117	11	97.0 ± 1.9	3.0 ± 1.9
NH 352	Cat. No. 117	9	94.0 ± 2.2	5.0 ± 2.2
NH 355	Cat. No. 117	9	93.2 ± 1.9	6.8 ± 1.9
NH 358	Cat. No. 117	7	93.8 ± 1.9	6.2 ± 1.9
NH 363	Cat. No. 119	6	98.0 ± 0.4	2.0 ± 0.4
NH 364	Cat. No. 119	10	98.2 ± 0.8	1.8 ± 0.8
NH 379	Cat. No. 119	7	97.8 ± 0.3	2.0 ± 0.3
NH 381	Cat. No. 119	7	97.7 ± 0.9	2.3 ± 0.9
NH 383	Cat. No. 119	9	98.3 ± 0.6	1.7 ± 0.3
NH 385	Cat. No. 119	10	96.0 ± 1.7	4.0 ± 1.7
NH 386	Cat. No. 119	6	98.4 ± 0.3	1.6 ± 0.3
NH 387	Cat. No. 119	7	97.1 ± 0.8	2.9 ± 0.8
NH 389	Cat. No. 119	7	97.7 ± 0.5	2.3 ± 0.5
NH 391	Cat. No. 119	8	96.9 ± 1.1	3.1 ± 1.1
IMJ 34341	Cat. No. 156	12	100	–
IMJ 34778	Cat. No. 164	6	94.5 ± 2.7	5.5 ± 2.7
IMJ 34836	Cat. No. 164	6	95.3 ± 2.5	4.7 ± 2.5
IMJ 34877	Cat. No. 164	6	92.4 ± 2.2	7.6 ± 2.2
NH 429	Cat. No. 168	9	99.6 ± 0.7	0.4 ± 0.7
NH 453	Cat. No. 169	7	97.2 ± 1.5	2.8 ± 1.5
IMJ 34197	Cat. No. 178	4	89.3 ± 6.4	10.7 ± 6.4
IMJ 34831	Cat. No. 186	6	96.9 ± 0.7	3.1 ± 0.7
IMJ 34833	Cat. No. 186	6	95.3 ± 0.9	4.7 ± 0.9
IMJ 34410	Cat. No. 186	6	96.4 ± 1.4	3.6 ± 1.4
IMJ 34411	Cat. No. 186	6	98.0 ± 1.1	2.0 ± 1.1
IMJ 34413	Cat. No. 186	6	97.8 ± 0.7	2.2 ± 0.7
NH 468	Cat. No. 200	9	97.5 ± 0.5	2.5 ± 0.5
NH 469	Cat. No. 200	9	96.9 ± 0.9	3.1 ± 0.9
NH 471	Cat. No. 200	6	94.8 ± 1.4	5.2 ± 1.4
NH 472	Cat. No. 200	9	98.3 ± 0.6	1.7 ± 0.6
IMJ 34421	Cat. No. 220	6	100	–
IMJ 34425	Cat. No. 231	6	97.3 ± 1.1	2.7 ± 1.1
IMJ 34426	Cat. No. 231	3	96.4	3.6
IMJ 34496	Cat. No. 286	6	98.5 ± 0.5	1.5 ± 0.5
IMJ 34497	Cat. No. 286	6	98.1 ± 1.0	1.9 ± 1.0
NH 499	Cat. No. 295	9	97.8 ± 0.9	2.2 ± 0.9
NH 501	Cat. No. 295	11	96.4 ± 1.8	3.6 ± 1.8
NH 522	Cat. No. 297	6	96.9 ± 2.2	3.1 ± 2.2
NH 560	Cat. No. 310	6	98.1 ± 0.3	1.9 ± 0.3
NH 565	Cat. No. 310	6	96.4 ± 1.3	3.6 ± 1.3
IMJ 34487	SID.ST. 35	6	96.3 ± 2.4	3.7 ± 2.4
IMJ 34503	UNC. 3	6	99.9 ± 0.3	0.1 ± 0.3

**Table 4:** The average alloy composition (wt% content) of the different groups (Cat. Nos.) of coins from public collections according to SEM-EDS analysis after omitting the peaks of oxides, corrosion products, and soil elements. Plated coins were not included in the average alloy composition calculations.

Coin Type	No. of Coins	No. of EDS Measurements	Average Silver Content in the Alloy (wt% Ag)	Average Copper Content in the Alloy (wt% Cu)
Cat. No. 1	3	24	95.7 ± 3.6	4.3 ± 3.6
Cat. No. 18	3	23	98.8 ± 1.7	1.2 ± 1.7
Cat. No. 21	2	14	95.2 ± 3.5	4.8 ± 3.5
Cat. No. 22	2	7	97.8 ± 1.6	2.2 ± 1.6
Cat. No. 23	4	30	96.7 ± 1.2	3.3 ± 1.2
Cat. No. 39	2	12	93.8 ± 5.0	6.2 ± 5.0
Cat. No. 48	2	12	98.0 ± 5.3	2.0 ± 5.3
Cat. No. 104	3	18	99.8 ± 0.5	0.2 ± 0.5
Cat. No. 109	4	24	97.2 ± 2.1	2.8 ± 2.1
Cat. No. 112	4	23	98.9 ± 2.2	1.1 ± 2.2
Cat. No. 117	9	74	94.4 ± 3.7	5.6 ± 3.7
Cat. No. 119	10	77	97.6 ± 1.2	2.4 ± 1.2
Cat. No. 134	3	23	96.2 ± 2.8	3.8 ± 2.8
Cat. No. 138	2	12	97.5 ± 1.1	2.5 ± 1.1
Cat. No. 154	2	12	98.1 ± 0.7	1.9 ± 0.7
Cat. No. 155	3	18	99.0 ± 1.4	1.0 ± 1.4
Cat. No. 156 (isolated)	1	12	100	–
Cat. No. 157	2	12	98.0 ± 1.5	2.0 ± 1.5
Cat. No. 158	2	18	98.5 ± 0.5	1.5 ± 0.5
Cat. No. 162	2	16	95.0 ± 2.3	5.0 ± 2.3
Cat. No. 163	2	14	97.1 ± 1.3	2.9 ± 1.3
Cat. No. 164	3	18	94.0 ± 2.7	6.0 ± 2.7
Cat. No. 168 (isolated)	1	9	99.6 ± 0.7	0.4 ± 0.7
Cat. No. 169 (isolated)	1	7	97.2 ± 1.5	2.8 ± 1.5
Cat. No. 178 (isolated)	1	4	89.3	10.7
Cat. No. 181	2	12	92.1 ± 4.2	7.9 ± 4.2
Cat. No. 183	2	14	98.2 ± 0.7	1.8 ± 0.7
Cat. No. 186	6	30	96.9 ± 1.4	3.1 ± 1.4
Cat. No. 200	4	33	97.1 ± 1.5	2.9 ± 1.5
Cat. No. 221	2	12	98.0 ± 1.5	2.0 ± 1.5
Cat. No. 220 (isolated)	1	6	100	–
Cat. No. 231	2	9	97.2 ± 0.8	2.8 ± 0.8
Cat. No. 246	2	12	98.8 ± 1.6	1.2 ± 1.6
Cat. No. 286	2	12	98.3 ± 0.8	1.7 ± 0.8
Cat. No. 295	3	20	97.0 ± 1.6	3.0 ± 1.6
Cat. No. 297 (isolated)	1	6	96.9 ± 2.2	3.1 ± 2.2
Cat. No. 299	2	12	95.4 ± 3.4	4.6 ± 3.4
Cat. No. 309	4	32	97.2 ± 1.2	2.8 ± 1.2
Cat. No. 310	2	12	97.3 ± 1.3	2.7 ± 1.3
Cat. No. SID.ST. 35 (isolated)	1	6	96.3 ± 2.4	3.7 ± 2.4
UNC. 3 (isolated)	1	6	99.9 ± 0.3	0.1 ± 0.3

**Table 5:** The average alloy composition of the jewelry (Samples 1–14), sheets (15–22), striated ingot (Sample 23), Sidonian coin (Sample 24), and Philistian coin (Sample 25) from the late-fifth-century BCE hacksilber hoard according to SEM-EDS analysis after omitting the peaks of oxides, corrosion products, and soil elements.

Object	No. of EDS Measurements	Average Ag (wt%) Content in the Alloy	Average Cu (wt%) Content in the Alloy	Average Au (wt%) Content in the Alloy
Ornamented ring (or earring), Sample 1	4	95.1 ± 0.8	1.8 ± 0.6	3.1 ± 0.2
Ornamented earrings, Samples 2–6	14	92.2 ± 6.9	4.2 ± 6.2	3.6 ± 4.9
Decorated lunate earring, Sample 7	3	76.0	1.4	22.6
Bead made of granules, Sample 8	4	100	–	–
Bead, joints between granules, Sample 8	5	100	–	–
Cylindrical bead, granules, Sample 9	5	99.7 ± 0.6	0.3 ± 0.6	–
Cylindrical bead, joints between granules, Sample 9	8	99.6 ± 0.7	0.4 ± 0.7	–
Circular/spiral bead, Sample 10	4	90.8 ± 5.8	8.0 ± 5.8	1.2 ± 1.3
Oval-shaped decorated bezel rings, Samples 11–13	20	92.0 ± 2.3	3.5 ± 1.2	4.5 ± 2.6
Broken ring fragments, Sample 14	21	95.9 ± 2.6	1.2 ± 1.7	2.9 ± 2.1
Sheet, Sample 15	3	95.2	2.1	2.7
Sheet, Sample 16	4	94.3 ± 1.8	4.1 ± 2.6	1.6 ± 1.0
Sheet, Sample 17	3	97.0	0.9	2.1
Sheet, Sample 18	3	96.5	0.8	2.7
Sheet, Sample 19	3	100	–	–
Sheet, Sample 20	4	85.8 ± 8.7	14.2 ± 8.7	–
Sheet, Sample 21	3	95.3	6.2	0.5
Sheet, Sample 22	7	97.4 ± 0.5	2.3 ± 0.7	0.3 ± 0.7
Sheets, Samples 15–22	30	95.2 ± 5.5	3.2 ± 5.8	1.6 ± 1.7
Striated ingot, Sample 23	3	97.5	0.4	2.1
Sidonian coin, Sample 24	8	99.8 ± 0.5	0.2 ± 0.5	–
Philistian coin, Sample 25	5	99.7 ± 0.4	0.3 ± 0.4	–

## Data Availability

The original contributions presented in this study are included in the article and Supplementary Materials. Further inquiries can be directed to the corresponding author.

## Supplementary Materials

The following supporting information can be downloaded at: https://www.mdpi.com/article/10.3390/ma18071678/s1, Table S1: SEM-EDS analysis results of the group of coins of Cat. No. 1 (IMJ 34127, IMJ 34183, IMJ 34809), where SA represents the scanned area; Table S2: SEM-EDS analysis results of the Cat. No. 18 (IMJ 34184, IMJ 34343, IMJ 34344/plated coin, IMJ 34345/plated coin, and IMJ 34803), where SA represents the scanned area. The plated coins IMJ 34344 and IMJ 34345 were not included in the average alloy composition calculations of Cat. No. 18; Table S3: SEM-EDS analysis results of the Cat. No. 112’s coins (IMJ 34807, IMJ 34808, IMJ 34353, IMJ 34354), where SA represents the scanned area. One measurement (IMJ 34353, obverse, area 1) of dark oxide layer was not included in the average composition calculations; Table S4: SEM-EDS analysis results of the Cat. No. 117’s group of coins (NH 340, NH 343, NH 345, NH 346, NH 347, NH 348, NH 352, NH 355, NH 358), where SA represents the scanned area; Table S5: SEM-EDS analysis results of the Cat. No. 119 group of coins (NH 363, NH 364, NH 379, NH 381, NH 383, NH 385, NH 386, NH 387, NH 389, NH 391), where SA represents the scanned area. Two measurements of areas covered with dark corrosion products (coin NH 364, reverse, areas 4 and 5) were not included in the average alloy composition calculations of this group; Table S6: SEM-EDS analysis results of Cat. No. 164 group of coins (IMJ 34778; IMJ 34836; IMJ 34877), where SA represents the scanned area; Table S7: SEM-EDS analysis results of five Cat. No. 186 specimens, where SA represents the scanned area. This group included the following coins: IMJ 34831, IMJ 34833, IMJ 34410, IMJ 34411, and IMJ 34413. Specimen IMJ 34412 was not included in the average composition calculations of this group because it was a plated coin; Table S8: SEM-EDS analysis results of Cat. No. 200’s group of coins (NH 468, NH 469, NH 471, and NH 472), where SA represents the scanned area; Table S9: SEM-EDS analysis results of Cat. No. 231 group of coins (IMJ 34425, IMJ 34426), where SA represents the scanned area. Two measurements of areas covered with dark corrosion products (one area of coin IMJ 34425 and one area of coin IMJ 34426) were not included in the average alloy composition calculations of Cat. No. 231 due to the presence of dark corrosion products (according to BSE mode); Table S10: SEM-EDS analysis results of Cat. No. 286 group of coins (IMJ 34496, IMJ 34497, IMJ 34398), where SA represents the scanned area. Coin IMJ 34498 was not included in the Cat. No. 286 calculations of the average alloy composition because it was a plated issue (with presence of between 26.3 wt% Cu and 78.2 wt% Cu); Table S11: SEM-EDS analysis results of Cat. No. 295 group of coins (NH 499, NH 501, NH 502), where SA represents the scanned area. Coin NH 502 was not included in the calculations because of its unusual heterogeneous results (presence of 1.7–62.7 wt% Cu); Table S12: SEM-EDS analysis results of Cat. No. 310 group of coins (NH 560, NH 565), where SA represents the scanned area. One measurement of coin NH 560 obverse was not included in the average composition calculations of Cat. No. 310 since it contained corrosion products and therefore had an unusual composition; Table S13: SEM-EDS analysis results of the isolated specimens (NH 429/Cat. No. 168, NH 453/Cat. No. 169, NH 522/Cat. No. 297, IMJ 34197/Cat. No. 178, IMJ 34341/Cat. No. 156, IMJ 34421/Cat. No. 220, IMJ 34424/Cat. No. 222, IMJ 34487/Cat. No. SID.ST. 35, IMJ 34503 /UNC. 3/IMJ), where SA represents the scanned area [Note: coin IMJ 34424/Cat. No. 222 was not included in the average alloy composition since it was a copper coin plated with silver]; Figure S1: The silver jewelry, hacksilber, and coins from the hacksilber hoard: (a) earring/ring (Sample 1) decorated with a flower like rod, (b–f) lunate earrings (Samples 2–6), (g) lunate earring decorated with granules (Sample 7), (h) bead made of granules (Sample 8), (i) cylindrical bead decorated with granules (Sample 9), (j) spiral bead (Sample 10), (k–m) rings with decorated oval bezel (Samples 11–13), (n) four parts of a broken ring (Sample 14); Figure S2: SEM images of the ornamented earring (or ring), Sample 1: (a,b) the item’s cast bar surrounded by a rod shaped into a flower (SE mode and BSE mode, respectively) and (c,d) the rod that was wrapped around the item in order to decorate the ornamented earring or ring (SE mode and BSE mode, respectively). The well-preserved bright areas (according to BSE mode) were examined by SEM-EDS analysis; Table S14: SEM-EDS analysis results of silver jewelry, where SA represents the scanned area; Figure S3: SEM images of the two-layer granulated bead (Sample 8): (a,b) top view of the upper layer of granules (SE mode and BSE mode, respectively) and (c,d) isometric view of the two layers of granules (SE mode and BSE mode, respectively); Figure S4: SEM images of the cylindrical bead decorated with two granulated beads (Sample 9): (a,b) general view left and center parts (SE mode and BSE mode, respectively) of the items, (c) higher magnification of the cylindrical bead joint line, (d) higher magnification of the left granulated bead surface, (e) top view of the left granulated bead, and (f) higher magnification of the joint area between two granules (top view); Figure S5: SEM images of a ring with a decorated oval front (Sample 11) showing (a,b) the zigzag line and dot decorations at the front of the ring (SE mode and BSE mode, respectively) and (c,d) the fracture surface at the broken back of the ring (SE mode and BSE mode, respectively); Figure S6: SEM images of a ring with oval-shaped decorated bezel (Sample 12) showing (a,b) the fracture surface of the broken back of the ring (SE mode and BSE mode, respectively) and (c,d) higher magnification of the fracture surface (SE mode and BSE mode, respectively); Table S15: SEM-EDS analysis results of silver sheets, where SA represents the scanned area; Table S16: SEM-EDS analysis results of the two coins (Sidonian and Philistian), where SA represents the scanned area.
