# Supplementary material for: A Material Study of Persian-Period Silver Coins and Hacksilber from Samaria
Source: Materials (Basel). 2025 Apr 7;18(7):1678. doi: 10.3390/ma18071678 (PMC11990297; doi:10.3390/ma18071678)
Supplement: Supplementary file 1 [file materials-18-01678-s001.zip › materials-3463417-Supplementary Materials.pdf]

# Electronic Supplementary Material – Materials, MDPI

# A Material Study of Persian-Period Provincial Samarian Silver Coins and Hacksilber from Samaria

**Dana Ashkenazi [1,*], Maayan Cohen [2,3], Haim Gitler [4], Mati Johananoff [2], Oren Tal [2]**

[1] Tel Aviv University, Tel Aviv 6997801, Israel. ORCID: 0000-0001-5871-1903
[2] Department of Archaeology and Ancient Near Eastern Cultures, Tel Aviv University, Ramat Aviv 6997801, Israel
[3] Leon Recanati Institute for Maritime Studies, University of Haifa 3498838, Israel
[4] The Israel Museum, Derech Rupin 11, Jerusalem 9171002, Israel

*Corresponding author: danaa@tauex.tau.ac.il

## 1. Characterization of the Materials of the Samarian Coinage

In this Electronic Supplementary Material file, SEM-EDS analysis results of the Persian period silver coins (Cat. Nos. 1,18, 112, 117, 119, 164, 186, 200, 231, 286, 295, 310, and isolated specimens) from Samaria, held in public collections (mainly from the Israel Museum Jerusalem [IMJ]), are presented (Tables S1–S13), showing the wt% composition of the coins' surface, as well as the alloy composition after omitting the peaks of oxides, corrosion products and soil elements.

**Table S1.** SEM-EDS analysis results of the group of coins of Cat. No. 1 (IMJ 34127, IMJ 34183, IMJ 34809), where SA represents the scanned area.

| Sample | Composition (wt %) | | | | | | | |
|---|---|---|---|---|---|---|---|---|
| | Surface | | | | | | Silver alloy | |
| | Ag | Cu | O | Si | Cl | Others | Ag | Cu |
| IMJ 34127, obverse, ground bulk metal, SA 1: 300 μm × 300 μm | 78.0 | 8.6 | 11.3 | – | 2.1 | – | 90.1 | 9.9 |
| IMJ 34127, obverse, ground bulk metal, SA 2: 200 μm × 200 μm | 81.9 | 7.5 | 8.3 | – | 2.3 | – | 91.6 | 8.4 |
| IMJ 34127, obverse, ground bulk metal, SA 3: 1000 μm × 1000 μm | 81.7 | 7.4 | 8.3 | 0.5 | 2.1 | – | 91.7 | 8.3 |
| IMJ 34127, reverse, ground bulk metal, SA 1: 100 μm × 100 μm | 85.7 | 6.5 | 7.2 | 0.4 | 0.2 | – | 93.0 | 7.0 |
| IMJ 34127, reverse, ground bulk metal, SA 2: 100 μm × 100 μm | 82.8 | 7.2 | 9.2 | 0.4 | 0.4 | – | 92.0 | 8.0 |
| IMJ 34127, reverse, ground bulk metal, SA 3: 100 μm × 100 μm | 87.6 | 5.9 | 6.2 | 0.3 | – | – | 93.7 | 6.3 |
| IMJ 34183, obverse, SA 1: 10 μm × 10 μm | 97.5 | 2.5 | – | – | – | – | 97.5 | 2.5 |
| IMJ 34183, obverse, SA 2: 100 μm × 100 μm | 87.1 | 1.4 | 9.4 | 0.6 | – | 1.5 Al | 98.4 | 1.6 |
| IMJ 34183, obverse, SA 3: 150 μm × 150 μm | 91.5 | – | 6.6 | 0.5 | 0.6 | 0.8 Al | 100 | – |
| IMJ 34183, reverse, SA 1: 300 μm × 300 μm | 89.9 | – | 7.7 | 0.9 | 1.1 | 0.4 Al | 100 | – |
| IMJ 34183, reverse, SA 2: 300 μm × 300 μm | 85.5 | 0.2 | 8.1 | 0.7 | 5.0 | 0.5 Al | 99.8 | 0.2 |

| | | | | | | | | |
|---|---|---|---|---|---|---|---|---|
| IMJ 34183, obverse, ground bulk metal, SA 1: 100 μm × 100 μm | 84.6 | 6.8 | 8.0 | 0.6 | – | – | 92.6 | 7.4 |
| IMJ 34183, obverse, ground (bulk metal), SA 2: 50 μm × 50 μm | 84.2 | 2.9 | 10.7 | 0.8 | 0.4 | 0.5 S, 0.5 Ca | 96.7 | 3.3 |
| IMJ 34183, obverse, SA 3: 50 μm × 50 μm | 85.7 | – | 9.2 | 0.6 | 3.8 | 0.4 Al, 0.3 S | 100 | – |
| IMJ 34183, obverse, SA 4: 50 μm × 50 μm | 87.5 | – | 7.1 | 0.8 | 4.1 | 0.5 Al | 100 | – |
| IMJ 34183, reverse, ground (bulk metal), SA 1: 50 μm × 50 μm | 83.6 | 0.7 | 11.2 | 0.8 | 2.1 | 0.8 S, 0.8 Ca | 99.2 | 0.8 |
| IMJ 34183, reverse, ground (bulk metal), SA 2: 50 μm × 50 μm | 80.2 | 1.2 | 12.9 | 1.1 | – | 0.4 Al, 3.6 S, 0.6 Ca | 98.5 | 1.5 |
| IMJ 34809, obverse, ground (bulk metal), SA 1: 100 μm × 100 μm | 70.9 | 2.0 | 5.3 | 0.8 | 20.3 | 0.7 Al | 97.3 | 2.7 |
| IMJ 34809, obverse, ground bulk metal, SA 2: 100 μm × 100 μm | 66.9 | 4.2 | 8.0 | 1.1 | 19.0 | 0.7 Al | 94.1 | 5.9 |
| IMJ 34809, obverse, SA 3: 300 μm × 300 μm | 74.0 | 3.3 | 4.3 | – | 18.4 | – | 95.7 | 4.3 |
| IMJ 34809, obverse, SA 4: 200 μm × 200 μm | 71.1 | 2.2 | 5.2 | – | 21.5 | – | 97.0 | 3.0 |
| IMJ 34809, reverse, SA 1: 100 μm × 100 μm | 85.2 | 7.4 | 6.4 | – | 1.0 | – | 92.0 | 8.0 |
| IMJ 34809, reverse, SA 2: 100 μm × 100 μm | 80.6 | 11.2 | 6.8 | 0.4 | 1.0 | – | 87.9 | 12.1 |
| IMJ 34809, reverse, SA 3: 100 μm × 100 μm | 72.3 | 1.7 | 4.8 | 0.6 | 20.1 | 0.5 Al | 97.7 | 2.3 |

**Table S2.** SEM-EDS analysis results of the Cat. No. 18 (IMJ 34184, IMJ 34343, IMJ 34344/plated coin, IMJ 34345/plated coin, and IMJ 34803), where SA represents the scanned area. The plated coins were not included in the average alloy composition calculations of Cat. No. 18.

| Sample | Composition (wt %) | | | | | | | |
|---|---|---|---|---|---|---|---|---|
| | Surface | | | | | | Silver alloy | |
| | Ag | Cu | O | Si | Cl | Others | Ag | Cu |
| IMJ 34184, reverse, area 2, SA: 100 µm × 100 µm | 86.5 | – | 11.6 | 0.6 | 0.3 | 1.0 Ca | 100 | – |
| IMJ 34184, reverse, area 3, SA: 100 µm × 100 µm | 84.9 | – | 12.8 | 1.0 | 0.2 | 0.4 Al, 0.7 Ca | 100 | – |
| IMJ 34184, obverse, area 1, SA: 500 µm × 500 µm | 87.7 | – | 10.1 | 0.8 | 0.3 | 0.4 Al, 0.7 Ca | 100 | – |
| IMJ 34184, obverse, area 2, SA: 200 µm × 200 µm | 89.2 | – | 9.2 | 0.6 | 0.2 | 0.3 Al, 0.5 Ca | 100 | – |
| IMJ 34184, obverse, area 3, SA: 300 µm × 300 µm | 85.7 | – | 11.6 | 0.7 | 0.3 | 0.5 Al, 1.2 Ca | 100 | – |
| IMJ 34343, reverse, area 1, SA: 500 µm × 500 µm | 78.2 | 1.7 | 6.5 | 0.7 | 12.5 | 0.4 Al | 97.9 | 2.1 |
| IMJ 34343, reverse, area 2, SA: 500 µm × 500 µm | 83.9 | 1.5 | 3.9 | 0.3 | 10.4 | – | 98.2 | 1.8 |
| IMJ 34343, reverse, area 3, SA: 400 µm × 400 µm | 70.2 | 1.6 | 8.5 | 1.2 | 17.4 | 1.1 Al | 97.8 | 2.2 |
| IMJ 34343, reverse, area 4, SA: 500 µm × 500 µm | 85.7 | 1.8 | 4.2 | – | 8.3 | – | 97.9 | 2.1 |
| IMJ 34343, reverse, area 5, SA: 300 µm × 300 µm | 82.5 | 2.0 | 4.9 | 0.4 | 9.8 | 0.4 Al | 97.6 | 2.4 |
| IMJ 34343, reverse, area 6, SA: 500 µm × 500 µm | 69.7 | 1.4 | 9.2 | 1.3 | 17.7 | 0.7 Al | 98.0 | 2.0 |
| IMJ 34343, obverse, area 1, SA: 400 µm × 400 µm | 73.5 | 1.3 | 4.8 | 0.6 | 19.3 | 0.5 Al | 98.3 | 1.7 |
| IMJ 34343, obverse, area 2, SA: 100 µm × 100 µm | 83.7 | 7.1 | 8.3 | 0.2 | 0.7 | – | 92.2 | 7.8 |
| IMJ 34343, obverse, area 3, SA: 400 µm × 400 µm | 68.8 | 1.3 | 10.5 | 1.2 | 17.1 | 1.1 Al | 98.1 | 1.9 |
| IMJ 34343, obverse, area 4, SA: 500 µm × 500 µm | 74.8 | – | 4.8 | 0.4 | 19.7 | 0.3 Al | 100 | – |
| IMJ 34343, obverse, area 5, SA: 500 µm × 500 µm | 80.8 | 1.5 | 7.1 | 0.6 | 9.5 | 0.5 Al | 98.2 | 1.8 |
| IMJ 34343, obverse, area 6, SA: 100 µm × 100 µm | 71.6 | 1.4 | 9.5 | 1.3 | 15.7 | 0.5 Al | 98.1 | 1.9 |
| IMJ 34344 (plated coin), reverse, area 1, SA: 100 µm × 100 µm, light area | 37.6 | 19.4 | 24.5 | 5.0 | 12.5 | 0.4 Al, 0.6 Fe | 66.0 | 34.0 |
| IMJ 34344, reverse, area 2, SA: 200 µm × 200 µm, light area | 43.5 | 14.0 | 26.3 | 3.7 | 11.4 | 0.5 Al, 0.6 Ca | 75.7 | 24.3 |
| IMJ 34344, reverse, area 3, SA: 200 µm × 200 µm, light area | 43.3 | 11.8 | 30.1 | 3.6 | 9.2 | 0.7 Al, 1.3 Ca | 78.6 | 21.4 |
| IMJ 34344, reverse, area 4, SA: 100 µm × 100 µm, dark area | 1.7 | 13.6 | 47.9 | 17.9 | 0.4 | 4.0 Al, 10.6 Ca, 0.8 P, 0.9 K, 2.2 Fe | 11.1 | 88.9 |

| | | | | | | | | |
|---|---|---|---|---|---|---|---|---|
| IMJ 34344, reverse, area 5, SA: 100 µm × 100 µm, dark area | 1.3 | 13.1 | 47.8 | 14.4 | 0.4 | 3.4 Al, 14.9 Ca, 1.2 P, 0.8 K, 0.7 Mg, 2.0 Fe | 9.0 | 91.0 |
| IMJ 34344, reverse, area 6, SA: 100 µm × 100 µm, dark area | 2.4 | 10.6 | 48.5 | 19.7 | 0.4 | 4.4 Al, 8.5 Ca, 0.7 P, 1.2 K, 0.7 Mg, 2.9 Fe | 18.5 | 81.5 |
| IMJ 34344, obverse, area 1, SA: 100 µm × 100 µm, light area | 51.9 | 11.6 | 20.8 | 3.0 | 12.2 | 0.5 Al | 81.7 | 18.3 |
| IMJ 34344, obverse, area 2, SA: 400 µm × 400 µm, light area | 46.1 | 11.7 | 26.4 | 4.7 | 9.8 | 0.7 Al, 0.6 Ca | 79.7 | 20.3 |
| IMJ 34344, obverse, area 3, SA: 200 µm × 200 µm, light area | 27.7 | 22.0 | 30.9 | 10.3 | 6.9 | 0.7 Al, 1.5 Ca | 55.7 | 44.3 |
| IMJ 34344, obverse, area 4, SA: 500 µm × 500 µm, dark area | 1.3 | 12.9 | 44.2 | 21.5 | 0.4 | 5.0 Al, 9.2 Ca, 0.7 P, 1.4 K, 0.6 Mg, 2.8 Fe | 9.2 | 90.8 |
| IMJ 34344, obverse, area 5, SA: 100 µm × 100 µm, dark area | 1.2 | 12.5 | 45.8 | 24.3 | 0.4 | 5.2 Al, 4.2 Ca, 0.6 P, 1.4 K, 1.0 Mg, 3.4 Fe | 8.8 | 91.2 |
| IMJ 34344, obverse, area 6, SA: 100 µm × 100 µm, dark area | – | 29.3 | 33.1 | 19.4 | – | 4.5 Al, 7.6 Ca, 1.7 K, 4.4 Fe | – | 100 |
| IMJ 34345 (plated coin), reverse, area 1, SA: 400 µm × 400 µm, light area | 83.0 | 4.7 | 11.8 | – | 0.5 | – | 94.6 | 5.4 |
| IMJ 34345, reverse, area 2, SA: 200 µm × 200 µm, light area | 73.3 | 4.8 | 20.2 | 0.5 | 0.8 | 0.4 S | 93.8 | 6.2 |
| IMJ 34345, reverse, area 3, SA: 50 µm × 50 µm, light area | 58.9 | 22.2 | 13.5 | 0.4 | 0.4 | 4.6 S | 72.6 | 27.4 |
| IMJ 34345, reverse, area 4, SA: 500 µm × 500 µm, dark area | 3.5 | 72.7 | 16.7 | 2.9 | 0.9 | 1.5 Al, 0.4 Ca, 0.7 S, 0.7 Fe | 4.6 | 95.4 |
| IMJ 34345, reverse, area 5, SA: 500 µm × 500 µm, dark area | 1.7 | 76.4 | 17.2 | 1.6 | 1.4 | 1.0 Al, 0.3 Ca, 0.4 S | 2.2 | 97.8 |
| IMJ 34345, reverse, area 6, SA: 400 µm × 400 µm, dark area | 3.8 | 70.7 | 17.4 | 4.7 | 0.6 | 1.9 Al, 0.3 Ca, 0.6 S | 5.1 | 94.9 |
| IMJ 34345, obverse, area 1, SA: 50 µm × 50 µm, light area | 93.2 | 2.6 | 3.8 | – | 0.4 | – | 97.3 | 2.7 |
| IMJ 34345, obverse, area 2, SA: 500 µm × 500 µm, light area | 87.6 | 1.3 | 10.0 | 0.5 | 0.6 | – | 98.5 | 1.5 |
| IMJ 34345, obverse, area 3, SA: 200 µm × 200 µm, light area | 89.6 | 1.4 | 8.0 | 0.4 | 0.6 | – | 98.5 | 1.5 |
| IMJ 34345, obverse, area 4, SA: 400 µm × 400 µm, dark area | 1.8 | 81.1 | 13.6 | 1.5 | 0.7 | 0.8 Al, 0.5 S | 2.2 | 97.8 |
| IMJ 34345, obverse, area 5, SA: 1000 µm × 1000 µm, dark area | 1.0 | 81.6 | 13.7 | 1.3 | 0.9 | 1.1 Al, 0.4 S | 1.2 | 98.8 |
| IMJ 34345, obverse, area 6, SA: 1000 µm × 1000 µm, dark area | 2.5 | 77.0 | 14.7 | 2.4 | 0.6 | 1.2 Al, 0.6 S, 0.5 Ca, 0.5 Fe | 3.1 | 96.9 |
| IMJ 34803, reverse, area 1, SA: 200 µm × 200 µm | 71.6 | – | 6.3 | 0.4 | 20.9 | 0.8 Al | 100 | – |
| IMJ 34803, reverse, area 2, SA: 200 µm × 200 µm | 68.8 | – | 10.8 | 0.8 | 17.9 | 1.2 Al, 0.5 Ca | 100 | – |

| | | | | | | | | |
|---|---|---|---|---|---|---|---|---|
| IMJ 34803, reverse, area 3, SA: 100 μm × 100 μm | 70.7 | – | 8.6 | 0.8 | 18.5 | 1.0 Al, 0.4 Ca | 100 | – |
| IMJ 34803, obverse, area 1, SA: 100 μm × 100 μm | 70.8 | – | 8.0 | 0.3 | 19.8 | 0.5 Al, 0.6 Ca | 100 | – |
| IMJ 34803, obverse, area 2, SA: 100 μm × 100 μm | 69.8 | – | 8.8 | 0.5 | 19.7 | 0.6 Al, 0.6 Ca | 100 | – |
| IMJ 34803, obverse, area 3, SA: 100 μm × 100 μm | 67.8 | – | 12.5 | 1.3 | 16.4 | 1.2 Al, 0.8 Ca | 100 | – |

**Table S3.** SEM-EDS analysis results of the Cat. No. 112's coins (IMJ 34807, IMJ 34808, IMJ 34353, IMJ 34354), where SA represents the scanned area. One measurement (IMJ 34353, obverse, area 1) of dark oxide layer was not included in the average composition calculations.

| Sample | Composition (wt %) | | | | | | | |
|---|---|---|---|---|---|---|---|---|
| | Surface | | | | | | Silver alloy | |
| | Ag | Cu | O | Si | Cl | Others | Ag | Cu |
| IMJ 34807, obverse, SA 1: 100 µm × 100 µm | 95.3 | – | 4.2 | – | 0.3 | 0.2 S | 100 | – |
| IMJ 34807, obverse, SA 2: 200 µm × 200 µm | 93.7 | – | 5.5 | 0.4 | 0.2 | 0.2 S | 100 | – |
| IMJ 34807, obverse, SA 3: 50 µm × 50 µm | 94.6 | 1.4 | 3.9 | – | 0.1 | – | 98.5 | 1.5 |
| IMJ 34807, reverse, SA 1: 200 µm × 200 µm | 93.7 | 1.2 | 5.0 | – | 0.1 | – | 98.7 | 1.3 |
| IMJ 34807, reverse, SA 2: 500 µm × 500 µm | 91.2 | – | 7.4 | 0.2 | 0.4 | 0.8 S | 100 | – |
| IMJ 34807, reverse, SA 3: 200 µm × 200 µm | 93.8 | – | 5.6 | – | 0.1 | 0.5 S | 100 | – |
| IMJ 34808, obverse, SA 1: 100 µm × 100 µm | 75.3 | – | 6.9 | 0.7 | 10.9 | 1.0 Al, 5.2 S | 100 | – |
| IMJ 34808, obverse, SA 2: 100 µm × 100 µm | 77.2 | – | 7.2 | 0.6 | 7.2 | 0.8 Al, 7.0 S | 100 | – |
| IMJ 34808, obverse, SA 3: 100 µm × 100 µm | 69.0 | – | 10.3 | 0.6 | 17.9 | 1.0 Al, 1.2 Ca | 100 | – |
| IMJ 34808, reverse, SA 1: 100 µm × 100 µm | 70.3 | – | 9.8 | 0.4 | 16.3 | 1.0 Al, 0.6 P, 1.6 Ca | 100 | – |
| IMJ 34808, reverse, SA 2: 300 µm × 300 µm | 66.5 | – | 14.8 | 0.9 | 14.6 | 1.3 Al, 1.9 Ca | 100 | – |
| IMJ 34808, reverse, SA 3: 100 µm × 100 µm | 73.1 | – | 6.8 | 0.5 | 17.6 | 1.1 Al, 0.9 Ca | 100 | – |
| IMJ 34353, obverse, SA 1: 100 µm × 100 µm, dark area | 32.8 | 33.3 | 22.3 | 2.0 | 8.9 | 0.7 Al | 49.5 | 50.5 |
| IMJ 34353, obverse, SA 2: 100 µm × 100 µm | 66.8 | – | 11.1 | 0.7 | 18.8 | 0.7 Al, 1.9 Ca | 100 | – |
| IMJ 34353, obverse, SA 3: 50 µm × 50 µm | 58.7 | 7.2 | 13.9 | 1.7 | 16.5 | 1.0 Al, 1.0 Ca | 89.1 | 10.9 |
| IMJ 34353, reverse, SA 1: 100 µm × 100 µm | 67.4 | – | 11.4 | 0.9 | 18.4 | 0.7 Al, 1.2 Ca | 100 | – |
| IMJ 34353, reverse, SA 2: 100 µm × 100 µm | 65.3 | 1.5 | 12.2 | 1.4 | 17.5 | 0.8 Al, 1.3 Ca | 97.8 | 2.2 |
| IMJ 34353, reverse, SA 3: 100 µm × 100 µm | 73.1 | 0.6 | 4.7 | 0.5 | 20.5 | 0.6 Al | 99.2 | 0.8 |
| IMJ 34354, obverse, SA 1: 300 µm × 300 µm | 87.2 | 1.9 | 7.1 | 0.9 | 2.9 | – | 97.9 | 2.1 |
| IMJ 34354, obverse, SA 2: 200 µm × 200 µm | 90.1 | 1.9 | 4.9 | 0.8 | 2.3 | – | 97.9 | 2.1 |
| IMJ 34354, obverse, SA 3: 100 µm × 100 µm | 90.3 | 0.6 | 5.9 | 0.8 | 2.0 | 0.4 Al | 99.3 | 0.7 |
| IMJ 34354, reverse, SA 1: 100 µm × 100 µm | 91.1 | 1.7 | 4.5 | 0.9 | 1.5 | 0.3 Al | 98.2 | 1.8 |

| | | | | | | | | |
|---|---|---|---|---|---|---|---|---|
| IMJ 34354, reverse, SA 2: 300 μm × 300 μm | 88.6 | 0.8 | 7.5 | 1.1 | 2.0 | – | 99.1 | 0.9 |
| IMJ 34354, reverse, SA 3: 100 μm × 100 μm | 91.1 | 1.3 | 3.9 | 0.8 | 2.6 | 0.3 Al | 98.6 | 1.4 |

**Table S4.** SEM-EDS analysis results of the Cat. No. 117's group of coins (NH 340, NH 343, NH 345, NH 346, NH 347, NH 348, NH 352, NH 355, NH 358), where SA represents the scanned area.

| Sample | Composition (wt %) | | | | | | | |
|---|---|---|---|---|---|---|---|---|
| | Surface | | | | | | Silver alloy | |
| | Ag | Cu | O | Si | Cl | Others | Ag | Cu |
| NH 340, reverse, SA 1: 100 μm × 100 μm | 90.2 | 3.3 | 6.1 | – | 0.4 | – | 96.5 | 3.5 |
| NH 340, reverse, SA 2: 100 μm × 100 μm | 91.4 | 2.4 | 5.4 | – | 0.8 | – | 97.4 | 2.6 |
| NH 340, reverse, SA 3: 50 μm × 50 μm | 93.6 | 1.6 | 3.8 | – | 1.0 | – | 98.3 | 1.7 |
| NH 340, reverse, SA 4 (examined in 2015) | 74.6 | 4.0 | 21.4 | – | – | – | 96.5 | 3.5 |
| NH 340, obverse, SA 1: 100 μm × 100 μm | 87.9 | 2.3 | 8.5 | 0.3 | 0.9 | – | 97.5 | 2.5 |
| NH 340, obverse, SA 2: 100 μm × 100 μm | 89.2 | 1.7 | 7.3 | 0.5 | 1.3 | – | 98.1 | 1.9 |
| NH 340, obverse, SA 3: 100 μm × 100 μm | 91.6 | 1.7 | 5.8 | – | 0.9 | – | 98.2 | 1.8 |
| NH 340, obverse, SA 4 (examined in 2015) | 94.7 | 3.4 | 1.9 | – | – | – | 95.0 | 5.0 |
| NH 343, reverse, SA 1: 100 μm × 100 μm | 77.6 | 9.1 | 12.3 | – | 1.0 | – | 89.5 | 10.5 |
| NH 343, reverse, SA 2: 100 μm × 100 μm | 75.0 | 10.1 | 13.8 | – | 0.8 | 0.3 S | 88.1 | 11.9 |
| NH 343, reverse, SA 3: 100 μm × 100 μm | 81.6 | 5.0 | 11.6 | 0.3 | 1.5 | – | 94.2 | 5.8 |
| NH 343, reverse, SA 4: 100 μm × 100 μm | 78.6 | 7.9 | 11.9 | – | 1.6 | – | 90.9 | 9.1 |
| NH 343, reverse, SA 5 (examined in 2015) | 86.8 | 4.9 | 8.3 | – | – | – | 94.7 | 5.3 |
| NH 343, obverse, SA 1: 100 μm × 100 μm | 62.2 | 18.2 | 17.7 | – | 1.9 | – | 77.4 | 22.6 |
| NH 343, obverse SA 2: 100 μm × 100 μm | 72.5 | 15.1 | 11.7 | – | 0.7 | – | 82.8 | 17.2 |
| NH 343, obverse, SA 3: 100 μm × 100 μm | 79.2 | 2.7 | 15.0 | 0.3 | 1.6 | 0.8 Na, 0.4 S | 96.7 | 3.3 |
| NH 343, obverse, SA 4: 50 μm × 50 μm | 89.2 | 3.7 | 5.8 | – | 1.3 | – | 96.0 | 4.0 |
| NH 343, obverse, SA 5 (examined in 2015) | 63.5 | 12.6 | 22.7 | – | 1.2 | – | 83.5 | 16.5 |
| NH 345, reverse, SA 1: 400 μm × 400 μm | 79.3 | 5.7 | 13.4 | 0.3 | 1.0 | 0.3 Al | 93.3 | 6.7 |
| NH 345, reverse, SA 2: 500 μm × 500 μm | 80.5 | 5.2 | 14.0 | 0.3 | – | – | 93.9 | 6.1 |
| NH 345, reverse, SA 3: 300 μm × 300 μm | 79.0 | 4.4 | 15.1 | 0.4 | 0.3 | 0.4 Si, 0.4 Ca | 94.7 | 5.3 |
| NH 345, reverse, SA 4: 200 μm × 200 μm | 80.0 | 4.6 | 14.8 | 0.3 | 0.3 | – | 94.6 | 5.4 |

| | | | | | | | | |
|---|---|---|---|---|---|---|---|---|
| NH 345, obverse, SA 1: 500 µm × 500 µm | 78.2 | 5.7 | 15.1 | 0.3 | 0.7 | – | 93.2 | 6.8 |
| NH 345, obverse, SA 2: 200 µm × 200 µm | 79.4 | 5.4 | 13.8 | 0.5 | 0.9 | – | 93.6 | 6.4 |
| NH 345, obverse, SA 3: 300 µm × 300 µm | 81.3 | 3.9 | 13.4 | 0.4 | 1.0 | – | 95.4 | 4.6 |
| NH 346, reverse, SA 1: 500 µm × 500 µm | 84.0 | 5.0 | 10.1 | – | 0.6 | 0.3 S | 94.4 | 5.6 |
| NH 346, reverse, SA 2: 400 µm × 400 µm | 81.4 | 5.4 | 11.6 | 0.3 | 1.0 | 0.3 S | 93.8 | 6.2 |
| NH 346, reverse, SA 3: 200 µm × 200 µm | 82.7 | 5.2 | 10.7 | – | 0.6 | 0.4 S, 0.4 Al | 94.1 | 5.9 |
| NH 346, reverse, SA 4: 400 µm × 400 µm | 83.1 | 5.3 | 10.7 | – | 0.6 | 0.3 S | 94.0 | 6.0 |
| NH 346, obverse, SA 1: 400 µm × 400 µm | 84.9 | 4.7 | 10.1 | – | 0.3 | – | 94.8 | 5.2 |
| NH 346, obverse, SA 2: 500 µm × 500 µm | 81.5 | 5.7 | 11.3 | – | 1.1 | 0.4 S | 93.5 | 6.5 |
| NH 346, obverse, SA 3: 500 µm × 500 µm | 83.3 | 5.2 | 10.7 | – | 0.8 | – | 97.4 | 2.6 |
| NH 347, reverse, SA 1: 300 µm × 300 µm | 78.0 | 6.1 | 15.7 | – | 0.2 | – | 92.7 | 7.3 |
| NH 347, reverse, SA 2: 100 µm × 100 µm | 90.1 | 2.7 | 6.7 | – | 0.4 | – | 97.1 | 2.9 |
| NH 347, reverse, SA 3: 200 µm × 200 µm | 89.5 | 2.0 | 7.6 | – | 0.9 | – | 97.8 | 2.2 |
| NH 347, obverse, SA 1: 200 µm × 200 µm | 88.1 | 3.0 | 8.2 | – | 0.7 | – | 96.7 | 3.3 |
| NH 347, obverse, SA 2: 50 µm × 50 µm | 86.9 | 4.1 | 8.7 | – | 0.3 | | 99.6 | 0.4 |
| NH 347, obverse, SA 3: 200 µm × 200 µm | 90.8 | 1.9 | 6.5 | 0.3 | 0.5 | – | 97.9 | 2.1 |
| NH 348, reverse, SA 1: 200 µm × 200 µm | 88.9 | 1.6 | 7.5 | 0.3 | 1.7 | – | 98.2 | 1.8 |
| NH 348, reverse, SA 2: 200 µm × 200 µm | 90.1 | 1.4 | 6.3 | 0.2 | 2.0 | – | 98.5 | 1.5 |
| NH 348, reverse, SA 3: 200 µm × 200 µm | 89.5 | 1.5 | 7.4 | 0.3 | 1.3 | – | 98.4 | 1.6 |
| NH 348, reverse, SA 4 (examined in 2015) | 84.6 | 3.5 | 11.9 | – | – | – | 96.0 | 4.0 |
| NH 348, obverse, SA 1: 100 µm × 100 µm | 90.8 | 1.8 | 5.8 | – | 1.6 | – | 98.1 | 1.9 |
| NH 348, obverse, SA 2: 100 µm × 100 µm | 86.1 | 4.4 | 8.4 | – | 1.1 | – | 95.1 | 4.9 |
| NH 348, obverse, SA 3: 100 µm × 100 µm | 89.7 | 2.6 | 6.0 | 0.4 | 1.3 | – | 97.2 | 2.8 |
| NH 348, obverse, SA 4: 100 µm × 100 µm | 87.0 | 7.6 | 4.5 | – | 0.9 | – | 92.0 | 8.0 |
| NH 348, obverse, SA 5: 100 µm × 100 µm | 89.2 | 1.6 | 6.8 | 0.3 | 2.1 | – | 98.2 | 1.8 |

| | | | | | | | | |
|---|---|---|---|---|---|---|---|---|
| NH 348, obverse, SA 6: 100 µm × 100 µm | 86.9 | 2.2 | 8.3 | – | 2.6 | – | 97.5 | 2.5 |
| NH 348, reverse, SA 7 (examined in 2015) | 96.2 | 2.2 | – | – | 1.6 | – | 97.8 | 2.2 |
| NH 352, reverse, SA 1: 200 µm × 200 µm | 80.6 | 7.1 | 9.5 | – | 2.8 | – | 91.9 | 8.1 |
| NH 352, reverse, SA 2: 300 µm × 300 µm | 77.2 | 8.2 | 10.3 | – | 4.3 | – | 90.4 | 9.6 |
| NH 352, reverse, SA 3: 200 µm × 200 µm | 75.3 | 5.2 | 17.2 | 0.3 | 0.6 | 0.9 S, 0.5 Ca | 93.5 | 6.5 |
| NH 352, reverse, SA 4: 200 µm × 200 µm | 87.2 | 4.2 | 5.4 | – | 3.2 | – | 95.4 | 4.6 |
| NH 352, reverse, SA 5 (examined in 2015) | 87.4 | 2.7 | 6.5 | – | 3.4 | – | 97.0 | 3.0 |
| NH 352, obverse, SA 1: 200 µm × 200 µm | 87.4 | 3.0 | 7.4 | – | 2.2 | – | 96.7 | 3.3 |
| NH 352, obverse, SA 2: 100 µm × 100 µm | 85.3 | 3.3 | 9.0 | 0.4 | 1.7 | 0.3 S | 96.3 | 3.7 |
| NH 352, obverse, SA 3: 200 µm × 200 µm | 84.5 | 3.3 | 8.9 | 0.4 | 2.5 | 0.4 S | 96.2 | 3.8 |
| NH 352, reverse, SA 4 (examined in 2015) | 70.2 | 4.6 | 23.7 | 0.4 | 0.7 | 0.4 S | 93.8 | 6.2 |
| NH 355, reverse, SA 1: 150 µm × 150 µm | 75.8 | 8.3 | 15.0 | – | 0.4 | 0.5 S | 90.1 | 9.9 |
| NH 355, reverse, SA 2: 150 µm × 150 µm | 83.7 | 7.1 | 8.7 | – | 0.5 | – | 92.2 | 7.8 |
| NH 355, reverse, SA 3: 150 µm × 150 µm | 83.2 | 6.9 | 9.1 | – | 0.8 | – | 92.3 | 7.7 |
| NH 355, reverse, SA 4: 150 µm × 150 µm | 87.2 | 5.4 | 6.6 | – | 0.8 | – | 94.2 | 5.8 |
| NH 355, reverse, SA 5 (examined in 2015) | 92.7 | 4.3 | 3.0 | – | – | – | 95.6 | 4.4 |
| NH 355, reverse, SA 6 (examined in 2015) | 71.8 | 7.2 | 20.4 | – | – | 0.6 S | 90.9 | 9.1 |
| NH 355, obverse, SA 1: 100 µm × 100 µm | 86.6 | 5.1 | 7.4 | – | 0.9 | – | 94.4 | 5.6 |
| NH 355, obverse, SA 2: 100 µm × 100 µm | 90.8 | 3.7 | 5.1 | – | 0.4 | – | 96.1 | 3.9 |
| NH 355, obverse, SA 3: 100 µm × 100 µm | 85.2 | 6.5 | 7.2 | – | 1.1 | – | 92.9 | 7.1 |
| NH 358, reverse, SA 1: 200 µm × 200 µm | 86.1 | 4.1 | 9.5 | – | 0.3 | – | 95.5 | 4.5 |
| NH 358, reverse, SA 2: 200 µm × 200 µm | 81.3 | 5.3 | 13.1 | – | 0.3 | – | 93.9 | 6.1 |
| NH 358, reverse, SA 3: 300 µm × 300 µm | 84.6 | 4.5 | 10.4 | – | 0.5 | – | 94.9 | 5.1 |
| NH 358, obverse, SA 1: 100 µm × 100 µm | 83.9 | 5.6 | 10.1 | – | 0.4 | – | 93.7 | 6.3 |
| NH 358, obverse, SA 2: 100 µm × 100 µm | 80.7 | 9.6 | 8.2 | – | 1.5 | – | 89.4 | 10.6 |

| | | | | | | | | | |
|---|---|---|---|---|---|---|---|---|---|
| NH 358, obverse, SA 3: 100 μm × 100 μm | 83.4 | 5.3 | 10.7 | – | 0.3 | 0.3 S | | 94.0 | 6.0 |
| NH 358, reverse, SA 1: 200 μm × 200 μm | 86.1 | 4.1 | 9.5 | – | 0.3 | – | | 95.5 | 4.5 |

**Table S5.** SEM-EDS analysis results of the Cat. No. 119 group of coins (NH 363, NH 364, NH 379, NH 381, NH 383, NH 385, NH 386, NH 387, NH 389, NH 391), where SA represents the scanned area. Two measurements of areas covered with dark corrosion products (coin NH 364, reverse, areas 4 and 5) were not included in the average alloy composition calculations of this group.

| Sample | Composition (wt %) | | | | | | | |
|---|---|---|---|---|---|---|---|---|
| | Surface | | | | | | Silver alloy | |
| | Ag | Cu | O | Si | Cl | Others | Ag | Cu |
| NH 363, reverse, SA 1: 300 μm × 300 μm | 90.3 | 1.7 | 4.8 | 0.3 | 2.9 | – | 98.2 | 1.8 |
| NH 363, reverse, SA 2: 300 μm × 300 μm | 85.6 | 1.3 | 6.8 | 0.4 | 5.9 | – | 98.5 | 1.5 |
| NH 363, reverse, SA 3: 200 μm × 200 μm | 86.4 | 2.3 | 6.3 | – | 5.0 | – | 97.4 | 2.6 |
| NH 363, obverse, SA 1: 200 μm × 200 μm | 89.3 | 1.9 | 6.1 | – | 2.7 | – | 97.9 | 2.1 |
| NH 363, obverse, SA 2: 1000 μm × 1000 μm | 89.7 | 1.5 | 4.7 | – | 4.1 | – | 98.4 | 1.6 |
| NH 363, obverse, SA 3: 100 μm × 100 μm | 92.0 | 2.5 | 4.0 | – | 1.5 | – | 97.4 | 2.6 |
| NH 364, reverse, SA 1: 300 μm × 300 μm | 93.2 | 1.3 | 4.6 | – | 0.9 | – | 98.6 | 1.4 |
| NH 364, reverse, SA 2: 300 μm × 300 μm | 88.9 | 1.1 | 8.9 | – | 1.1 | – | 98.8 | 1.2 |
| NH 364, reverse, SA 3: 300 μm × 300 μm | 93.2 | 1.1 | 4.9 | 0.3 | 0.5 | – | 98.8 | 1.2 |
| NH 364, reverse, SA 4 (covered with dark corrosion): 100 μm × 100 μm | 42.8 | 29.0 | 23.3 | 0.4 | 4.5 | – | 59.6 | 40.4 |
| NH 364, reverse, SA 5 (covered with dark corrosion, examined in 2015) | 51.2 | 13.2 | 31.5 | – | 3.4 | 0.7 S | 78.0 | 22.0 |
| NH 364, reverse, SA 6 (examined in 2015) | 87.8 | 1.2 | 9.5 | – | 1.5 | – | 98.7 | 1.3 |
| NH 364, reverse, SA 7 (examined in 2015) | 84.5 | 2.5 | 4.9 | – | 8.1 | – | 97.1 | 2.9 |
| NH 364, reverse, SA 8 (examined in 2015) | 80.1 | 2.9 | 11.2 | – | 5.8 | – | 96.5 | 3.5 |
| NH 364, obverse, SA 1: 200 μm × 200 μm | 92.0 | 0.9 | 5.8 | – | 1.0 | 0.3 S | 99.0 | 1.0 |
| NH 364, obverse, SA 2: 200 μm × 200 μm | 89.2 | 2.2 | 7.9 | – | 0.7 | – | 97.6 | 2.4 |
| NH 364, obverse, SA 3: 200 μm × 200 μm | 94.2 | 0.9 | 4.5 | – | 0.4 | – | 99.1 | 0.9 |
| NH 364, obverse, SA 4 (examined in 2015) | 88.5 | 1.8 | 9.1 | – | 0.6 | – | 98.0 | 2.0 |
| NH 379, reverse, SA 1: 300 μm × 300 μm | 95.0 | 1.3 | 3.3 | – | 0.4 | – | 98.7 | 1.3 |
| NH 379, reverse, SA 2: 300 μm × 300 μm | 91.5 | 2.1 | 5.7 | – | 0.7 | – | 97.8 | 2.2 |

| | | | | | | | | |
|---|---|---|---|---|---|---|---|---|
| NH 379, reverse, SA 3: 300 µm × 300 µm | 93.0 | 2.0 | 4.2 | – | 0.8 | – | 97.9 | 2.1 |
| NH 379, reverse, SA 4: 300 µm × 300 µm | 91.0 | 1.8 | 6.3 | 0.4 | 0.5 | – | 98.1 | 1.9 |
| NH 379, obverse, SA 1: 300 µm × 300 µm | 93.0 | 2.1 | 4.4 | – | 0.5 | – | 97.8 | 2.2 |
| NH 379, obverse, SA 2: 300 µm × 300 µm | 86.0 | 1.8 | 10.6 | 0.4 | 0.6 | 0.6 S | 97.1 | 2.1 |
| NH 379, obverse, SA 3: 300 µm × 300 µm | 87.1 | 2.2 | 9.0 | 0.5 | 0.7 | 0.5 S | 97.5 | 2.5 |
| NH 381, reverse, SA 1: 300 µm × 300 µm | 84.9 | 2.5 | 9.6 | 0.7 | 1.9 | 0.4 S | 97.1 | 2.9 |
| NH 381, reverse, SA 2: 300 µm × 300 µm | 89.5 | 2.6 | 6.0 | – | 0.9 | 0.4 S, 0.6 Ca | 97.2 | 2.8 |
| NH 381, reverse, SA 3: 300 µm × 300 µm | 89.8 | 2.3 | 6.8 | – | 1.1 | – | 96.7 | 3.3 |
| NH 381, reverse, SA 4: 300 µm × 300 µm | 89.5 | 2.6 | 5.8 | – | 2.1 | – | 97.2 | 2.8 |
| NH 381, obverse, SA 1: 300 µm × 300 µm | 93.1 | 2.1 | 4.5 | – | 0.3 | – | 97.8 | 2.2 |
| NH 381, obverse, SA 2: 300 µm × 300 µm | 90.5 | 0.6 | 6.9 | 0.3 | 1.7 | – | 99.3 | 0.7 |
| NH 381, obverse, SA 3: 300 µm × 300 µm | 91.0 | 1.3 | 5.4 | 0.3 | 2.0 | – | 98.6 | 1.4 |
| NH 383, reverse, SA 1: 300 µm × 300 µm | 91.1 | 1.3 | 7.1 | – | 0.5 | – | 98.6 | 1.4 |
| NH 383, reverse, SA 2: 300 µm × 300 µm | 89.5 | 1.0 | 7.6 | 0.4 | 1.5 | – | 98.9 | 1.1 |
| NH 383, reverse, SA 3: 300 µm × 300 µm | 89.2 | 1.8 | 8.2 | – | 0.8 | – | 98.0 | 2.0 |
| NH 383, reverse, SA 4: 300 µm × 300 µm | 91.8 | 0.7 | 6.8 | – | 0.7 | – | 99.2 | 0.8 |
| NH 383, reverse, SA 5 (examined in 2015) | 98.6 | 1.4 | – | – | – | – | 98.6 | 1.4 |
| NH 383, obverse, SA 1: 300 µm × 300 µm | 90.9 | 1.9 | 6.9 | – | 0.2 | – | 98.0 | 2.0 |
| NH 383, obverse, SA 2: 300 µm × 300 µm | 90.8 | 2.8 | 6.2 | – | 0.3 | – | 97.0 | 3.0 |
| NH 383, obverse, SA 3: 300 µm × 300 µm | 90.6 | 1.0 | 7.3 | 0.4 | 0.7 | – | 98.9 | 1.1 |
| NH 383, reverse, SA 4 (examined in 2015) | 97.1 | 2.1 | – | – | 0.8 | – | 97.9 | 2.1 |
| NH 385, reverse, SA 1: 300 µm × 300 µm | 89.5 | 3.3 | 5.9 | – | 1.4 | – | 96.4 | 3.6 |
| NH 385, reverse, SA 2: 300 µm × 300 µm | 88.3 | 2.8 | 7.3 | – | 1.3 | 0.4 S | 96.9 | 3.1 |
| NH 385, reverse, SA 3: 300 µm × 300 µm | 85.7 | 2.4 | 9.1 | – | 2.2 | 0.6 S | 97.3 | 2.7 |
| NH 385, reverse, SA 4: 300 µm × 300 µm | 88.3 | 2.8 | 6.9 | – | 2.0 | – | 96.9 | 3.1 |

| | | | | | | | | |
|---|---|---|---|---|---|---|---|---|
| NH 385, reverse, SA 5 (examined in 2015) | 83.5 | 4.7 | 7.4 | – | 4.4 | – | 94.7 | 5.3 |
| NH 385, obverse, SA 1: 300 µm × 300 µm | 89.3 | 3.2 | 6.5 | – | 1.0 | – | 96.5 | 3.5 |
| NH 385, obverse, SA 2: 300 µm × 300 µm | 81.1 | 7.1 | 8.3 | – | 3.5 | – | 91.9 | 8.1 |
| NH 385, obverse, SA 3: 300 µm × 300 µm | 92.4 | 3.4 | 3.5 | – | 0.7 | – | 96.5 | 3.5 |
| NH 385, obverse, SA 4 (examined in 2015) | 84.7 | 4.7 | 8.4 | – | 2.2 | – | 94.7 | 5.3 |
| NH 386, reverse, SA 1: 300 µm × 300 µm | 88.3 | 1.8 | 8.0 | 0.4 | 0.6 | 0.9 Ca | 98.0 | 2.0 |
| NH 386, reverse, SA 2: 300 µm × 300 µm | 91.0 | 1.2 | 6.8 | 0.4 | 0.6 | – | 98.7 | 1.3 |
| NH 386, reverse, SA 3: 300 µm × 300 µm | 86.6 | 1.4 | 11.6 | – | 0.4 | – | 98.6 | 1.4 |
| NH 386, reverse, SA 4: 300 µm × 300 µm | 91.5 | 1.4 | 6.8 | – | 0.3 | – | 98.5 | 1.5 |
| NH 386, obverse, SA 1: 300 µm × 300 µm | 91.1 | 1.9 | 6.7 | – | 0.3 | – | 98.0 | 2.0 |
| NH 386, obverse, SA 2: 300 µm × 300 µm | 86.2 | 1.1 | 10.8 | 0.3 | 1.1 | 0.5 S | 98.7 | 1.3 |
| NH 386, obverse, SA 3: 300 µm × 300 µm | 84.4 | 1.6 | 12.7 | 0.4 | 0.9 | – | 98.1 | 1.9 |
| NH 387, reverse, SA 1: 300 µm × 300 µm | 89.3 | 2.4 | 7.3 | – | 1.0 | – | 97.4 | 2.6 |
| NH 387, reverse, SA 2: 300 µm × 300 µm | 89.0 | 3.1 | 7.5 | – | 0.4 | – | 96.6 | 3.4 |
| NH 387, reverse, SA 3: 300 µm × 300 µm | 86.2 | 1.9 | 10.4 | – | 1.5 | – | 97.8 | 2.2 |
| NH 387, reverse, SA 4: 300 µm × 300 µm | 91.7 | 0.9 | 6.6 | – | 0.8 | – | 97.2 | 2.8 |
| NH 387, obverse, SA 1: 300 µm × 300 µm | 86.0 | 2.8 | 10.2 | – | 1.0 | – | 96.8 | 3.2 |
| NH 387, obverse, SA 2: 300 µm × 300 µm | 84.8 | 3.8 | 10.5 | – | 0.9 | – | 95.7 | 4.3 |
| NH 387, obverse, SA 3: 300 µm × 300 µm | 93.4 | 1.5 | 4.9 | – | 0.2 | – | 98.4 | 1.6 |
| NH 389, reverse, SA 1: 300 µm × 300 µm | 92.6 | 1.5 | 5.7 | – | 0.2 | – | 98.4 | 1.6 |
| NH 389, reverse, SA 2: 300 µm × 300 µm | 91.6 | 1.5 | 6.5 | – | 0.4 | – | 98.4 | 1.6 |
| NH 389, reverse, SA 3: 300 µm × 300 µm | 89.7 | 2.2 | 7.9 | – | 0.2 | – | 97.7 | 2.3 |
| NH 389, reverse, SA 4: 300 µm × 300 µm | 83.8 | 2.6 | 12.6 | – | 1.0 | – | 97.0 | 3.0 |
| NH 389, obverse, SA 1: 300 µm × 300 µm | 89.3 | 2.3 | 7.2 | 0.4 | 0.2 | 0.6 S | 97.5 | 2.5 |
| NH 389, obverse, SA 2: 300 µm × 300 µm | 90.7 | 2.0 | 6.6 | 0.4 | 0.3 | – | 97.8 | 2.2 |

| | | | | | | | | |
|---|---|---|---|---|---|---|---|---|
| NH 389, obverse, SA 3: 300 µm × 300 µm | 87.5 | 2.4 | 7.5 | 0.6 | 0.3 | 1.7 S | 97.3 | 2.7 |
| NH 391, reverse, SA 1: 300 µm × 300 µm | 85.0 | 4.2 | 10.0 | – | 0.3 | 0.5 S | 95.2 | 4.7 |
| NH 391, reverse, SA 2: 300 µm × 300 µm | 90.7 | 2.8 | 6.1 | – | 0.4 | – | 97.0 | 3.0 |
| NH 391, reverse, SA 3, SA: 300 µm × 300 µm | 92.5 | 2.3 | 4.9 | – | 0.3 | – | 97.6 | 2.4 |
| NH 391, reverse, SA 4: 300 µm × 300 µm | 84.8 | 2.4 | 10.9 | – | 0.4 | 0.6 S, 0.9 Ca | 97.3 | 2.7 |
| NH 391, reverse, SA 5 (examined in 2015) | 89.8 | 1.3 | 6.5 | – | 2.4 | – | 98.6 | 1.4 |
| NH 391, obverse, SA 1: 300 µm × 300 µm | 90.1 | 3.2 | 6.4 | – | 0.3 | – | 96.6 | 3.4 |
| NH 391, obverse, SA 2: 300 µm × 300 µm | 91.0 | 4.6 | 4.3 | – | 0.1 | – | 95.2 | 4.8 |
| NH 391, obverse, SA 3: 300 µm × 300 µm | 85.8 | 1.8 | 11.0 | – | 1.0 | 0.4 Ca | 97.9 | 2.1 |

**Table S6.** SEM-EDS analysis results of Cat. No. 164 group of coins (IMJ 34778; IMJ 34836; IMJ 34877), where SA represents the scanned area.

| Sample | Composition (wt %) | | | | | | | |
|---|---|---|---|---|---|---|---|---|
| | Surface | | | | | | Silver alloy | |
| | Ag | Cu | O | Si | Cl | Others | Ag | Cu |
| IMJ 34778, obverse, ground bulk metal, SA 1: 100 µm × 100 µm | 92.0 | 3.1 | 5.0 | – | – | – | 96.7 | 3.3 |
| IMJ 34778, obverse, ground bulk metal, SA 2: 100 µm × 100 µm | 84.7 | 9.5 | 5.8 | – | – | – | 89.9 | 10.1 |
| IMJ 34778, obverse, ground bulk metal, SA 3: 200 µm × 200 µm | 88.5 | 1.9 | 8.9 | 0.3 | – | 0.3 S | 97.9 | 2.1 |
| IMJ 34778, reverse, ground bulk metal, SA 1: 100 µm × 100 µm | 89.2 | 4.7 | 6.1 | – | – | – | 95.0 | 5.0 |
| IMJ 34778, reverse, ground bulk metal, SA 2: 200 µm × 200 µm | 87.1 | 7.1 | 5.8 | – | – | – | 92.5 | 7.5 |
| IMJ 34778, reverse, ground bulk metal, SA 3: 200 µm × 200 µm | 90.0 | 4.6 | 5.4 | – | – | – | 95.1 | 4.9 |
| IMJ 34836, obverse, ground bulk metal, SA 1: 300 µm × 300 µm | 85.5 | 2.2 | 3.6 | – | 8.7 | – | 97.5 | 2.5 |
| IMJ 34836, obverse, ground bulk metal, SA 2: 200 µm × 200 µm | 77.9 | 8.5 | 6.4 | – | 7.1 | – | 90.2 | 9.8 |
| IMJ 34836, obverse, ground bulk metal, SA 3: 100 µm × 100 µm | 85.7 | 3.8 | 4.1 | – | 6.4 | – | 95.8 | 4.2 |
| IMJ 34836, reverse, ground bulk metal, SA 1: 200 µm × 200 µm | 85.8 | 2.1 | 2.8 | – | 9.3 | – | 97.6 | 2.4 |
| IMJ 34836, reverse, ground bulk metal, SA 2: 200 µm × 200 µm | 78.2 | 4.0 | 3.7 | – | 14.2 | – | 95.1 | 4.9 |
| IMJ 34836, reverse, ground bulk metal, SA 3: 100 µm × 100 µm | 82.5 | 4.1 | 5.6 | – | 7.8 | – | 95.3 | 4.7 |
| IMJ 34877, obverse, ground bulk metal, SA 1: 300 µm × 300 µm | 85.0 | 8.0 | 6.6 | 0.4 | – | – | 91.4 | 8.6 |
| IMJ 34877, obverse, ground bulk metal, SA 2: 300 µm × 300 µm | 83.9 | 4.8 | 9.8 | 1.5 | – | – | 94.6 | 5.4 |
| IMJ 34877, obverse, ground bulk metal, SA 3: 100 µm × 100 µm | 81.1 | 14.0 | 4.5 | 0.4 | – | – | 94.4 | 5.6 |
| IMJ 34877, reverse, ground bulk metal, SA 1: 200 µm × 200 µm | 83.2 | 6.8 | 8.7 | 1.3 | – | – | 92.4 | 7.6 |
| IMJ 34877, reverse, ground bulk metal, SA 2: 300 µm × 300 µm | 81.5 | 11.0 | 6.9 | – | 0.6 | – | 88.1 | 11.9 |
| IMJ 34877, reverse, ground bulk metal, SA 3: 100 µm × 100 µm | 87.2 | 6.3 | 5.9 | – | 0.6 | – | 93.3 | 6.7 |

**Table S7.** SEM-EDS analysis results of Cat. No. 186 specimens, where SA represents the scanned area. This group included the following coins: IMJ 34831, IMJ 34833, IMJ 34410, IMJ 34411, IMJ 34412, and IMJ 34413. Specimen IMJ 34412 was not included in the average composition calculations of this group because it was a plated coin.

| Sample | Composition (wt %) | | | | | | | |
|---|---|---|---|---|---|---|---|---|
| | Surface | | | | | | Silver alloy | |
| | Ag | Cu | O | Si | Cl | Others | Ag | Cu |
| IMJ 34831, reverse, area 1, SA: 100 µm × 100 µm | 87.5 | 3.5 | 8.4 | – | 0.6 | – | 96.2 | 3.8 |
| IMJ 34831, reverse, area 2, SA: 200 µm × 200 µm | 89.3 | 1.7 | 8.4 | 0.4 | 0.2 | – | 98.1 | 1.9 |
| IMJ 34831, reverse, area 3, SA: 100 µm × 100 µm | 92.9 | 2.5 | 4.0 | – | 0.6 | – | 97.4 | 2.6 |
| IMJ 34831, obverse, area 1, SA: 200 µm × 200 µm | 88.0 | 3.5 | 8.1 | – | 0.4 | – | 96.2 | 3.8 |
| IMJ 34831, obverse, area 2, SA: 100 µm × 100 µm | 89.4 | 3.2 | 6.3 | – | 0.7 | 0.4 S | 96.5 | 3.5 |
| IMJ 34831, obverse, area 3, SA: 200 µm × 200 µm | 89.7 | 2.8 | 6.5 | – | 0.5 | 0.5 S | 97.0 | 3.0 |
| IMJ 34833, reverse, area 1, SA: 200 µm × 200 µm | 89.8 | 4.4 | 4.2 | – | 0.4 | 1.2 S | 95.3 | 4.7 |
| IMJ 34833, reverse, area 2, SA: 100 µm × 100 µm | 87.1 | 5.2 | 5.1 | – | 1.0 | 1.6 S | 94.4 | 5.6 |
| IMJ 34833, reverse, area 3, SA: 100 µm × 100 µm | 87.8 | 3.0 | 7.2 | – | 1.5 | 0.5 S | 96.7 | 3.3 |
| IMJ 34833, obverse, area 1, SA: 200 µm × 200 µm | 87.6 | 5.4 | 4.2 | – | 0.5 | 2.3 S | 94.2 | 5.8 |
| IMJ 34833, obverse, area 2, SA: 200 µm × 200 µm | 88.6 | 4.7 | 6.0 | – | 0.2 | 0.5 S | 95.0 | 5.0 |
| IMJ 34833, obverse, area 3, SA: 100 µm × 100 µm | 90.6 | 3.7 | 4.7 | – | 0.6 | 0.4 S | 96.1 | 3.9 |
| IMJ 34410, reverse, area 1, SA: 100 µm × 100 µm | 68.7 | 2.0 | 8.6 | 0.3 | 18.4 | 2.0 Al | 97.2 | 2.8 |
| IMJ 34410, reverse, area 2, SA: 100 µm × 100 µm | 81.3 | 4.7 | 4.8 | – | 8.5 | 0.7 Al | 94.5 | 5.5 |
| IMJ 34410, reverse, area 3, SA: 100 µm × 100 µm | 65.6 | 2.6 | 10.6 | 0.4 | 17.9 | 2.9 Al | 96.2 | 3.8 |
| IMJ 34410, obverse, area 1, SA: 200 µm × 200 µm | 76.4 | 2.9 | 7.5 | 0.8 | 12.0 | 0.4 Al | 96.3 | 3.7 |
| IMJ 34410, obverse, area 2, SA: 200 µm × 200 µm | 64.8 | 3.2 | 11.1 | 0.4 | 18.9 | 1.6 Al | 95.3 | 4.7 |
| IMJ 34410, obverse, area 3, SA: 100 µm × 100 µm | 81.9 | 0.8 | 5.6 | 0.3 | 10.9 | 0.5 Al | 99.0 | 1.0 |
| IMJ 34411, reverse, area 1, SA: 200 µm × 200 µm | 85.2 | 2.0 | 9.1 | 1.1 | 1.7 | 0.7 Al, 0.2 S | 97.7 | 2.3 |
| IMJ 34411, reverse, area 2, SA: 200 µm × 200 µm | 87.9 | 2.2 | 6.3 | 0.8 | 2.4 | 0.4 Al | 97.6 | 2.4 |
| IMJ 34411, reverse, area 3, SA: 100 µm × 100 µm | 88.6 | 1.0 | 7.6 | 0.3 | 1.6 | 0.5 Al, 0.4 C | 98.8 | 1.2 |

| | | | | | | | | |
|---|---|---|---|---|---|---|---|---|
| IMJ 34411, obverse, area 1, SA: 100 μm × 100 μm | 85.9 | 3.7 | 7.0 | 1.4 | 1.7 | 0.3 Al | 95.9 | 4.1 |
| IMJ 34411, obverse, area 2, SA: 200 μm × 200 μm | 79.4 | 1.0 | 15.2 | 0.6 | 2.3 | 0.7 Al, 0.3 S, 0.5 Ca | 98.8 | 1.2 |
| IMJ 34411, obverse, area 3, SA: 100 μm × 100 μm | 82.4 | 0.9 | 9.9 | 0.8 | 4.7 | 0.7 Al, 0.6 S | 98.9 | 1.1 |
| IMJ 34412, reverse, area 1, SA: 300 μm × 300 μm | 44.7 | 22.3 | 27.7 | 2.2 | 2.7 | 0.4 Ca | 66.7 | 33.3 |
| IMJ 34412, reverse, area 2, SA: 100 μm × 100 μm | 45.0 | 22.5 | 26.6 | 1.5 | 1.3 | 0.3 Al, 0.8 S, 0.6 Ca, 1.4 Fe | 66.7 | 33.3 |
| IMJ 34412, reverse, area 3, SA: 100 μm × 100 μm | 38.1 | 28.2 | 26.9 | 2.6 | 2.3 | 0.5 Al, 1.4 Pb | 57.5 | 42.5 |
| IMJ 34412, obverse, area 1, SA: 300 μm × 300 μm | 45.1 | 18.2 | 27.6 | 3.7 | 2.3 | 0.5 Al, 0.7 Ca, 1.9 Pb | 71.2 | 28.8 |
| IMJ 34412, obverse, area 2, SA: 200 μm × 200 μm | 2.5 | 51.0 | 32.7 | 11.2 | 0.6 | 0.4 S, 0.5 K, 0.5 Ca, 0.6 Fe | 4.7 | 95.3 |
| IMJ 34412, obverse, area 3, SA: 200 μm × 200 μm | 40.2 | 18.9 | 30.5 | 5.1 | 2.9 | 0.9 Al, 0.4 S, 1.1 Ca | 44.1 | 55.9 |
| IMJ 34413, reverse, area 1, SA: 400 μm × 400 μm | 63.9 | 1.6 | 15.7 | 1.5 | 15.4 | 1.1 Al, 0.8 Ca | 97.6 | 2.4 |
| IMJ 34413, reverse, area 2, SA: 100 μm × 100 μm | 69.7 | 1.1 | 8.5 | 1.2 | 18.3 | 0.9 Al, 0.3 Ca | 98.4 | 1.6 |
| IMJ 34413, reverse, area 3, SA: 200 μm × 200 μm | 69.8 | 2.5 | 9.9 | 0.8 | 15.8 | 0.7 Al, 0.5 Ca | 96.5 | 3.5 |
| IMJ 34413, obverse, area 1, SA: 50 μm × 50 μm | 64.2 | 1.7 | 14.4 | 1.4 | 16.2 | 1.5 Al, 0.6 Ca | 97.4 | 2.6 |
| IMJ 34413, obverse, area 2, SA: 100 μm × 100 μm | 67.7 | 0.9 | 12.1 | 1.2 | 15.5 | 1.8 Al, 0.8 Ca | 98.7 | 1.3 |
| IMJ 34413, obverse, area 3, SA: 100 μm × 100 μm | 69.3 | 1.3 | 9.9 | 1.1 | 17.3 | 1.1 Al | 98.2 | 1.8 |

**Table S8.** SEM-EDS analysis results of Cat. No. 200’s group of coins (NH 468, NH 469, NH 471 and NH 472), where SA represents the scanned area.

| Sample | Composition (wt %) | | | | | | | |
|---|---|---|---|---|---|---|---|---|
| | Surface | | | | | | Silver alloy | |
| | Ag | Cu | O | Si | Cl | Others | Ag | Cu |
| NH 468, reverse, SA 1: 400 µm × 400 µm | 90.1 | 1.6 | 6.8 | 0.3 | 1.2 | – | 98.2 | 1.8 |
| NH 468, reverse, SA 2: 500 µm × 500 µm | 89.1 | 2.3 | 7.3 | – | 1.3 | – | 97.5 | 2.5 |
| NH 468, reverse, SA 3: 300 µm × 300 µm | 92.0 | 2.2 | 5.0 | – | 0.8 | – | 97.7 | 2.3 |
| NH 468, reverse, SA 4: 200 µm × 200 µm | 92.7 | 2.4 | 4.6 | – | 0.3 | – | 97.5 | 2.5 |
| NH 468, reverse, SA 5 (examined in 2015) | 91.8 | 1.9 | 4.3 | – | 2.0 | – | 98.0 | 2.0 |
| NH 468, obverse, SA 1: 200 µm × 200 µm | 88.0 | 2.5 | 8.9 | – | 0.6 | – | 97.2 | 2.8 |
| NH 468, obverse, SA 2: 300 µm × 300 µm | 87.7 | 2.7 | 8.4 | – | 0.8 | 0.4 S | 97.0 | 3.0 |
| NH 468, obverse, SA 3: 300 µm × 300 µm | 86.0 | 3.1 | 10.5 | – | 0.4 | – | 96.5 | 3.5 |
| NH 468, obverse, SA 4 (examined in 2015) | 97.3 | 2.0 | – | – | 0.7 | – | 98.0 | 2.0 |
| NH 469, reverse, SA 1: 400 µm × 400 µm | 88.2 | 2.6 | 7.4 | 0.3 | 1.5 | – | 97.1 | 2.9 |
| NH 469, reverse, SA 2: 200 µm × 200 µm | 90.8 | 2.8 | 5.1 | – | 1.3 | – | 97.0 | 3.0 |
| NH 469, reverse, SA 3: 400 µm × 400 µm | 81.1 | 4.1 | 13.8 | – | 0.4 | 0.6 S | 95.2 | 4.8 |
| NH 469, reverse, SA 4: 200 µm × 200 µm | 88.2 | 2.5 | 7.5 | – | 1.8 | – | 97.2 | 2.8 |
| NH 469, reverse, SA 5 (examined in 2015) | 92.6 | 2.2 | 3.8 | – | 1.3 | – | 97.7 | 2.3 |
| NH 469, obverse, SA 1: 200 µm × 200 µm | 86.9 | 2.3 | 9.0 | 0.4 | 1.4 | – | 97.4 | 2.6 |
| NH 469, obverse, SA 2: 300 µm × 300 µm | 86.3 | 2.4 | 10.2 | – | 1.1 | – | 97.3 | 2.7 |
| NH 469, obverse, SA 3: 100 µm × 100 µm | 91.9 | 2.1 | 4.7 | 0.3 | 1.0 | – | 97.9 | 2.1 |
| NH 469, obverse, SA 4 (examined in 2015) | 85.1 | 3.9 | 10.1 | – | 0.9 | – | 95.6 | 4.4 |
| NH 471, reverse, SA 1: 200 µm × 200 µm | 88.8 | 2.9 | 7.9 | – | 0.4 | – | 96.8 | 3.2 |
| NH 471, reverse, SA 2: 200 µm × 200 µm | 81.7 | 4.4 | 13.4 | – | 0.5 | – | 94.9 | 5.1 |
| NH 471, reverse, SA 3: 100 µm × 100 µm | 85.3 | 4.2 | 9.8 | – | 0.7 | – | 95.3 | 4.7 |
| NH 471, obverse, SA 1: 300 µm × 300 µm | 82.4 | 3.7 | 12.8 | – | 0.7 | 0.4 Ca | 95.7 | 4.3 |

| | | | | | | | | |
|---|---|---|---|---|---|---|---|---|
| NH 471, obverse, SA 2: 500 µm × 500 µm | 74.2 | 6.3 | 18.5 | 0.3 | 0.7 | – | 92.2 | 7.8 |
| NH 471, obverse, SA 3: SA: 200 µm × 200 µm | 80.4 | 5.1 | 13.8 | 0.3 | 0.4 | – | 94.0 | 6.0 |
| NH 472, reverse, SA 1: 200 µm × 200 µm | 92.4 | 1.5 | 5.5 | 0.3 | 0.3 | – | 98.4 | 1.6 |
| NH 472, reverse, SA 2: 100 µm × 100 µm | 93.0 | 1.1 | 5.4 | – | 0.5 | – | 98.8 | 1.2 |
| NH 472, reverse, SA 3: 300 µm × 300 µm | 89.1 | 1.6 | 8.1 | 0.3 | 0.9 | – | 98.2 | 1.8 |
| NH 472, reverse, SA 4: 200 µm × 200 µm | 90.8 | 1.0 | 7.1 | 0.5 | 0.6 | – | 98.9 | 1.1 |
| NH 472, reverse, SA 5 (examined in 2015) | 94.6 | 0.9 | 3.9 | 0.2 | 0.4 | – | 99.1 | 0.9 |
| NH 472, obverse, SA 1: 100 µm × 100 µm | 91.8 | 1.7 | 5.6 | 0.5 | 0.4 | – | 98.2 | 1.8 |
| NH 472, obverse, SA 2: 200 µm × 200 µm | 91.5 | 1.6 | 6.5 | – | 0.4 | – | 98.3 | 1.7 |
| NH 472, obverse, SA 3: 200 µm × 200 µm | 91.0 | 2.0 | 6.6 | – | 0.4 | – | 97.8 | 2.2 |
| NH 472, reverse, SA 1 (examined in 2015) | 92.2 | 2.9 | 4.7 | – | 0.2 | – | 97.0 | 3.0 |

**Table S9.** SEM-EDS analysis results of Cat. No. 231 group of coins (IMJ 34425, IMJ 34426), where SA represents the scanned area. Two measurements of areas covered with dark corrosion products (one area of coin IMJ 34425 and one area of coin IMJ 34426) were not included in the average alloy composition calculations of Cat. No. 231 due to the presence of dark corrosion products (according to BSE mode).

| **Sample** | **Composition (wt %)** | | | | | | | |
|---|---|---|---|---|---|---|---|---|
| | **Surface** | | | | | | **Silver alloy** | |
| | **Ag** | **Cu** | **O** | **Si** | **Cl** | **Others** | **Ag** | **Cu** |
| IMJ 34425, reverse, area 1, SA: 400 μm × 400 μm | 63.2 | 3.1 | 6.0 | – | 25.1 | 2.6 S | 95.3 | 4.7 |
| IMJ 34425, reverse, area 2, SA: 400 μm × 400 μm | 69.0 | 2.0 | 8.2 | 0.3 | 18.1 | 0.2 Al, 1.8 S, 0.4 Ca | 97.2 | 2.8 |
| IMJ 34425, reverse, area 3, SA: 300 μm × 300 μm | 70.5 | 1.2 | 6.6 | – | 21.4 | 0.3 Al | 98.3 | 1.7 |
| IMJ 34425, reverse, area 4 (dark corroded area), SA: 200 μm × 200 μm | 8.0 | 5.0 | 52.3 | 1.1 | 2.3 | 0.3 Al, 0.5 Mg, 9.7 S, 2.3 Cl, 18.5 Ca | 61.5 | 38.5 |
| IMJ 34425, obverse, area 1, SA: 200 μm × 200 μm | 87.9 | 1.6 | 4.9 | – | 1.4 | 1.3 S, 2.9 Pb | 97.8 | 2.2 |
| IMJ 34425, obverse, area 2, SA: 500 μm × 500 μm | 79.2 | 3.0 | 7.2 | 0.2 | 7.1 | 3.3 S | 96.4 | 3.6 |
| IMJ 34425, obverse, area 3, SA: 200 μm × 200 μm | 71.8 | 1.1 | 5.2 | 0.3 | 20.9 | 0.7 S | 98.5 | 1.5 |
| IMJ 34426, reverse, area 1, SA: 400 μm × 400 μm | 89.4 | 3.4 | 5.8 | 0.5 | 0.9 | – | 96.3 | 3.7 |
| IMJ 34426, reverse, area 2, SA: 300 μm × 300 μm | 90.0 | 2.8 | 5.4 | 0.4 | 1.1 | 0.3 Al | 97.0 | 3.0 |
| IMJ 34426, reverse, area 3, SA: 200 μm × 200 μm | 90.4 | 3.8 | 5.1 | – | 0.7 | – | 96.0 | 4.0 |
| IMJ 34426, reverse, area 4 (dark area), SA: 200 μm × 200 μm | 69.1 | 5.4 | 14.5 | 0.7 | 0.2 | 1.2 Al, 8.9 S | 92.8 | 7.2 |

**Table S10.** SEM-EDS analysis results of Cat. No. 286 group of coins (IMJ 34496, IMJ 34497, IMJ 34398), where SA represents the scanned area. Coin IMJ 34498 was not included in the Cat. No. 286 calculations of the average alloy composition because it was a plated issue (with presence of between 26.3 wt% Cu and 78.2 wt% Cu).

| Sample | Composition (wt %) | | | | | | | |
|---|---|---|---|---|---|---|---|---|
| | Surface | | | | | | Silver alloy | |
| | Ag | Cu | O | Si | Cl | Others | Ag | Cu |
| IMJ 34496, reverse, area 1, SA: 300 μm × 300 μm | 93.4 | 1.2 | 5.3 | – | 0.1 | – | 98.7 | 1.3 |
| IMJ 34496, reverse, area 2, SA: 300 μm × 300 μm | 90.9 | 1.3 | 7.4 | – | 0.4 | – | 98.6 | 1.4 |
| IMJ 34496, reverse, area 3, SA: 500 μm × 500 μm | 90.7 | 1.4 | 7.0 | 0.3 | 0.6 | – | 98.5 | 1.5 |
| IMJ 34496, obverse, area 1, SA: 100 μm × 100 μm | 92.0 | 2.2 | 5.2 | – | 0.6 | – | 97.7 | 2.3 |
| IMJ 34496, obverse, area 2, SA: 200 μm × 200 μm | 94.5 | 0.7 | 4.6 | – | 0.2 | – | 99.3 | 0.7 |
| IMJ 34496, obverse, area 3, SA: 100 μm × 100 μm | 92.4 | 1.5 | 6.0 | – | 0.1 | – | 98.4 | 1.6 |
| IMJ 34497, reverse, area 1, SA: 100 μm × 100 μm | 88.3 | 2.2 | 8.6 | 0.3 | 0.6 | – | 97.6 | 2.4 |
| IMJ 34497, reverse, area 2, SA: 100 μm × 100 μm | 81.8 | 1.2 | 13.9 | 0.8 | 0.7 | 0.6 Al, 1.0 Ca | 98.6 | 1.4 |
| IMJ 34497, reverse, area 3, SA: 100 μm × 100 μm | 87.9 | 2.3 | 8.9 | – | 0.5 | 0.4 Ca | 97.4 | 2.6 |
| IMJ 34497, obverse, area 1, SA: 50 μm × 50 μm | 94.0 | 2.9 | 3.0 | – | 0.1 | – | 97.0 | 3.0 |
| IMJ 34497, obverse, area 2, SA: 50 μm × 50 μm | 91.1 | 2.0 | 6.8 | – | 0.1 | – | 97.8 | 2.2 |
| IMJ 34497, obverse, area 3, SA: 100 μm × 100 μm | 92.1 | – | 6.5 | 0.6 | 0.8 | – | 100 | – |
| IMJ 34498 (plated coin), reverse, area 1, SA: 200 μm × 200 μm | 10.2 | 36.6 | 27.1 | 14.0 | 0.6 | 3.8 Al, 2.7 Ca, 0.8 Mg, 0.5 P, 1.0 K, 2.2 Fe | 21.8 | 78.2 |
| IMJ 34498, reverse, area 2, SA: 200 μm × 200 μm | 22.6 | 18.6 | 36.7 | 9.9 | 2.2 | 3.3 Al, 4.0 Ca, 0.9 S, 0.6 K, 1.2 Fe | 54.9 | 45.1 |
| IMJ 34498, reverse, area 3, SA: 200 μm × 200 μm | 10.5 | 30.0 | 36.6 | 13.4 | 1.0 | 2.7 Al, 2.9 Ca, 0.4 P, 0.6 S, 0.5 K, 1.4 Fe | 25.9 | 74.1 |
| IMJ 34498, obverse, area 1, SA: 50 μm × 50 μm | 17.8 | 25.5 | 39.1 | 12.1 | 0.5 | 1.7 Al, 2.0 Ca, 0.6 Si, 2.0 Ca, 0.7 Fe | 41.1 | 58.9 |
| IMJ 34498, obverse, area 2, SA: 100 μm × 100 μm | 38.9 | 13.9 | 31.8 | 7.5 | 0.8 | 1.8 Al, 2.9 Ca, 0.6 Mg, 0.9 S, 0.9 Fe | 73.7 | 26.3 |
| IMJ 34498, obverse, area 3, SA: 100 μm × 100 μm | 35.7 | 17.8 | 27.4 | 5.6 | 5.6 | 2.1 Al, 4.3 Ca, 0.4 S, 1.1 Fe | 66.8 | 33.2 |

**Table S11.** SEM-EDS analysis results of Cat. No. 295 group of coins (NH 499, NH 501, NH 502), where SA represents the scanned area. Coin NH 502 was not included in the calculations because of its unusual heterogeneous results (presence of 1.7–62.7 wt% Cu).

| Sample | Composition (wt %) | | | | | | | |
|---|---|---|---|---|---|---|---|---|
| | Surface | | | | | | Silver alloy | |
| | Ag | Cu | O | Si | Cl | Others | Ag | Cu |
| NH 499, reverse, SA 1: 1000 µm × 1000 µm | 91.5 | 1.1 | 5.9 | – | 1.5 | – | 98.8 | 1.2 |
| NH 499, reverse, SA 2: 300 µm × 300 µm | 87.4 | 1.1 | 8.7 | – | 1.8 | 0.5 S, 0.5 Ca | 98.8 | 1.2 |
| NH 499, reverse, SA 3: 200 µm × 200 µm | 90.9 | 2.3 | 5.1 | – | 1.7 | – | 97.5 | 2.5 |
| NH 499, reverse, SA 4: 300 µm × 300 µm | 88.9 | 2.2 | 6.5 | 0.3 | 1.6 | 0.5 S | 97.8 | 2.2 |
| NH 499, reverse, SA 5 (examined in 2015) | 98.8 | 1.2 | – | – | – | – | 98.8 | 1.2 |
| NH 499, reverse, SA 6 (examined in 2015) | 97.5 | 1.9 | – | – | 0.6 | – | 98.1 | 1.9 |
| NH 499, obverse, SA 1: 100 µm × 100 µm | 89.3 | 3.5 | 6.3 | – | 0.5 | 0.4 S | 96.2 | 3.8 |
| NH 499, obverse, SA 2: 100 µm × 100 µm | 86.8 | 3.0 | 8.7 | – | 1.5 | – | 96.7 | 3.3 |
| NH 499, obverse, SA 3: 200 µm × 200 µm | 84.9 | 1.9 | 10.6 | – | 2.1 | 0.5 S | 97.8 | 2.2 |
| NH 501, reverse, SA 1: 300 µm × 300 µm | 91.1 | 3.2 | 5.1 | – | 0.6 | – | 96.6 | 3.4 |
| NH 501, reverse, SA 2: 100 µm × 100 µm | 87.5 | 4.1 | 7.2 | – | 0.7 | 0.5 S | 95.5 | 4.5 |
| NH 501, reverse, SA 3: 200 µm × 200 µm | 83.1 | 4.1 | 10.2 | 0.3 | 1.0 | 1.3 S | 95.3 | 4.7 |
| NH 501, reverse, SA 4: 200 µm × 200 µm | 87.3 | 4.1 | 7.5 | 0.3 | 0.8 | – | 95.5 | 4.5 |
| NH 501, reverse, SA 5 (examined in 2015) | 79.0 | 5.5 | 11.1 | 1.0 | 0.9 | 1.8 S, 0.7 Ca | 93.5 | 6.4 |
| NH 501, reverse, SA 6 (examined in 2015) | 95.2 | 4.1 | – | – | 0.7 | – | 95.9 | 4.1 |
| NH 501, obverse, SA 1: 500 µm × 500 µm | 90.8 | 1.0 | 7.2 | 0.3 | 0.7 | – | 98.9 | 1.1 |
| NH 501, obverse, SA 2: 300 µm × 300 µm | 93.6 | 1.2 | 4.6 | 0.2 | 0.4 | – | 98.7 | 1.3 |
| NH 501, obverse, SA 3: 200 µm × 200 µm | 92.5 | 0.9 | 6.1 | – | 0.5 | – | 99.0 | 1.0 |
| NH 501, obverse, SA 4 (examined in 2015) | 77.1 | 4.6 | 15.2 | – | 3.1 | – | 94.4 | 5.6 |
| NH 501, obverse, area 5 examined in 2015) | 96.8 | 3.2 | – | – | – | – | 96.8 | 3.2 |
| NH 502, reverse, SA 1: 200 µm × 200 µm | 77.4 | 8.5 | 8.4 | 1.1 | 4.2 | 0.4 S | 90.0 | 10.0 |
| NH 502, reverse, SA 2: 200 µm × 200 µm | 87.8 | 3.3 | 6.7 | 0.4 | 1.8 | – | 96.4 | 3.6 |

| | | | | | | | | |
|---|---|---|---|---|---|---|---|---|
| NH 502, reverse, SA 3 (area of corrosion): 100 µm × 100 µm | 59.1 | 21.6 | 7.7 | – | 11.3 | 0.3 S | 73.2 | 26.8 |
| NH 502, reverse, SA 4: 200 µm × 200 µm | 86.2 | 2.2 | 8.4 | 0.7 | 2.1 | 0.4 Al | 97.5 | 2.5 |
| NH 502, reverse, SA 5 (area of corrosion, examined in 2015) | 41.5 | 28.4 | 15.0 | – | 15.1 | – | 59.4 | 40.6 |
| NH 502, reverse, SA 6 (area of corrosion, examined in 2015) | 19.4 | 33.3 | 15.1 | 0.6 | 7.2 | 1.1 S, 1.4 Ca, 21.9 Sn | 37.3 | 62.7 |
| NH 502, reverse, SA 7 (area of corrosion, examined in 2015) | 79.1 | 4.3 | 12.5 | 0.7 | 3.4 | – | 94.8 | 5.2 |
| NH 502, obverse, SA 1: 300 µm × 300 µm | 90.2 | 1.6 | 5.2 | 0.4 | 2.6 | – | 98.3 | 1.7 |
| NH 502, obverse, SA 2: 200 µm × 200 µm | 89.8 | 1.9 | 5.1 | 0.4 | 2.8 | – | 97.9 | 2.1 |
| NH 502, obverse, SA 3: 300 µm × 300 µm | 88.3 | 2.1 | 6.2 | – | 3.4 | – | 97.7 | 2.3 |
| NH 502, obverse, SA 4 (examined in 2015) | 94.4 | 1.9 | – | – | 3.7 | – | 98.0 | 2.0 |
| NH 502, obverse, SA 5 (area of corrosion, examined in 2015) | 50.2 | 14.0 | 17.7 | 0.6 | 16.1 | 0.6 S, 0.8 Ca | 78.2 | 21.8 |

**Table S12.** SEM-EDS analysis results of Cat. No. 310 group of coins (NH 560, NH 565), where SA represents the scanned area. One measurement of coin NH 560 obverse was not included in the average composition calculations of Cat. No. 310 since it contained corrosion products and therefore had an unusual composition.

| Sample | Composition (wt %) | | | | | | | |
|---|---|---|---|---|---|---|---|---|
| | Surface | | | | | | Silver alloy | |
| | Ag | Cu | O | Si | Cl | Others | Ag | Cu |
| NH 560, reverse, area 1, SA: 200 μm × 200 μm | 91.9 | 1.7 | 5.6 | – | 0.8 | – | 98.2 | 1.8 |
| NH 560, reverse, area 2, SA: 200 μm × 200 μm | 90.8 | 1.8 | 6.5 | – | 0.9 | – | 98.1 | 1.9 |
| NH 560, reverse, area 3, SA: 300 μm × 300 μm | 88.9 | 2.2 | 7.3 | – | 1.6 | – | 97.6 | 2.4 |
| NH 560, obverse, area 1, SA: 200 μm × 200 μm | 90.9 | 1.6 | 6.1 | – | 1.4 | | 98.3 | 1.7 |
| NH 560, obverse, area 2, SA: 200 μm × 200 μm | 86.3 | 1.7 | 10.4 | – | 1.6 | – | 98.1 | 1.9 |
| NH 560, obverse, area 3, SA: 200 μm × 200 μm | 89.5 | 1.4 | 7.4 | – | 1.7 | – | 98.5 | 1.5 |
| NH 560, obverse, area 4, SA: 300 μm × 300 μm (corrosion products) | 62.2 | 2.1 | 24.4 | 1.2 | 2.5 | 1.2 Na, 0.8 Al, 0.7 S, 0.6 K, 1.5 Ca, 2.8 Au | 96.7 | 3.3 |
| NH 565, reverse, area 1, SA: 200 μm × 200 μm | 71.0 | 4.7 | 16.8 | – | 4.1 | 3.4 S | 93.8 | 6.2 |
| NH 565, reverse, area 2, SA: 200 μm × 200 μm | 86.0 | 2.5 | 8.9 | – | 2.6 | – | 97.2 | 2.8 |
| NH 565, reverse, area 3, SA: 100 μm × 100 μm | 81.4 | 3.6 | 10.1 | 0.4 | 4.3 | 0.2 S | 95.8 | 4.2 |
| NH 565, obverse, area 1, SA: 200 μm × 200 μm | 83.8 | 2.3 | 11.0 | – | 2.3 | 0.6 Ca | 97.3 | 2.7 |
| NH 565, obverse, area 2, SA: 200 μm × 200 μm | 78.5 | 2.6 | 14.5 | – | 3.8 | 0.6 S | 96.8 | 3.2 |
| NH 565, obverse, area 3, SA: 200 μm × 200 μm | 83.3 | 2.3 | 12.5 | – | 1.9 | – | 97.3 | 2.7 |

**Table S13.** SEM-EDS analysis results of the isolated specimens (NH 429/Cat. No. 168, NH 453/Cat. No. 169, NH 522/Cat. No. 297, IMJ 34197/Cat. No. 178, IMJ 34341/Cat. No. 156, IMJ 34421/Cat. No. 220, IMJ 34487/Cat. No. SID.ST. 35, IMJ 34503 /UNC. 3/IMJ, where SA represents the scanned area [Note: coin IMJ 34424/Cat. No. 222 was not included in the average alloy composition since it was a copper coin plated with silver.

| Sample | Composition (wt %) | | | | | | | |
|---|---|---|---|---|---|---|---|---|
| | Surface | | | | | | Silver alloy | |
| | Ag | Cu | O | Si | Cl | Others | Ag | Cu |
| NH 429, obverse, SA 1: 500 µm × 500 µm | 81.8 | – | 16.4 | – | 1.8 | – | 100 | – |
| NH 429, obverse, SA 2: 500 µm × 500 µm | 87.7 | – | 10.4 | – | 1.9 | – | 100 | – |
| NH 429, obverse, SA 3: 300 µm × 300 µm | 94.1 | – | 4.5 | – | 1.4 | – | 100 | – |
| NH 429, reverse, SA 1: 200 µm × 200 µm | 93.3 | – | 4.9 | 0.6 | 1.2 | – | 100 | – |
| NH 429, reverse, SA 2: 200 µm × 200 µm | 92.4 | – | 6.7 | – | 0.9 | – | 100 | – |
| NH 429, reverse, SA 3: 500 µm × 500 µm | 86.3 | – | 13.7 | – | – | – | 100 | – |
| NH 429, reverse, SA 4: 500 µm × 500 µm | 86.2 | – | 13.8 | – | – | – | 100 | – |
| NH 429, reverse, SA 5 (examined in 2015) | 93.0 | 1.5 | 4.0 | – | 1.5 | – | 98.4 | 1.6 |
| NH 429, reverse, SA 6 (examined in 2015) | 92.7 | 1.7 | 4.5 | – | 1.1 | – | 98.2 | 1.8 |
| NH 453, obverse, SA 1: 500 µm × 500 µm | 87.5 | 2.0 | 9.1 | – | 1.4 | – | 97.8 | 2.2 |
| NH 453, obverse, SA 2: 300 µm × 300 µm | 90.9 | – | 8.4 | – | 0.7 | – | 100 | – |
| NH 453, obverse, SA 3: 100 µm × 100 µm | 93.0 | 2.6 | 3.8 | – | 0.6 | – | 97.3 | 2.7 |
| NH 453, reverse, SA 1: 400 µm × 400 µm | 83.3 | 3.4 | 11.1 | 0.8 | 1.4 | – | 96.1 | 3.9 |
| NH 453, reverse, SA 2: 300 µm × 300 µm | 82.1 | 3.4 | 12.1 | 0.8 | 1.6 | – | 96.0 | 4.0 |
| NH 453, reverse, SA 3: 300 µm × 300 µm | 92.1 | 2.0 | 4.9 | – | 1.0 | – | 97.9 | 2.1 |
| NH 453, reverse, SA 4: 300 µm × 300 µm | 87.2 | 4.5 | 6.6 | 0.6 | 1.1 | – | 95.1 | 4.9 |
| NH 453, obverse, SA 1: 500 µm × 500 µm | 87.5 | 2.0 | 9.1 | – | 1.4 | – | 97.8 | 2.2 |
| NH 453, obverse, SA 2: 300 µm × 300 µm | 90.9 | – | 8.4 | – | 0.7 | – | 100 | – |
| NH 453, obverse, SA 3: 100 µm × 100 µm | 93.0 | 2.6 | 3.8 | – | 0.6 | – | 97.3 | 2.7 |
| NH 522, reverse, SA 1: 200 µm × 200 µm | 88.8 | 3.5 | 6.4 | 0.4 | 0.9 | – | 96.2 | 3.8 |
| NH 522, reverse, SA 2: 100 µm × 100 µm | 79.3 | 6.5 | 13.2 | 0.4 | 0.6 | – | 92.4 | 7.6 |

| | | | | | | | | |
|---|---|---|---|---|---|---|---|---|
| NH 522, reverse, SA 3: 100 µm × 1100 µm | 84.0 | 2.9 | 9.1 | 1.6 | 2.4 | – | 96.7 | 3.3 |
| IMJ 34197, obverse, SA 1: 100 µm × 100 µm | 87.5 | 6.1 | 6.4 | – | – | – | 93.5 | 6.5 |
| IMJ 34197, reverse, SA 1: 300 µm × 300 µm | 72.7 | 11.0 | 14.1 | – | 0.9 | 1.3 Na | 86.9 | 13.1 |
| IMJ 34197, reverse, SA 2: 200 µm × 200 µm | 60.5 | 13.9 | 22.1 | – | 1.1 | 2.4 Na | 81.3 | 18.7 |
| IMJ 34197, reverse, SA 3: 100 µm × 100 µm | 88.4 | 4.3 | 6.9 | – | 0.4 | – | 95.4 | 4.6 |
| IMJ 34341, obverse, SA 1: 100 µm × 100 µm | 91.6 | – | 6.3 | 0.6 | 1.0 | 0.5 Al | 100 | – |
| IMJ 34341, obverse, SA 2: 300 µm × 300 µm | 89.0 | – | 7.4 | 0.7 | 2.2 | 0.7 Al | 100 | – |
| IMJ 34341, obverse, SA 3: 200 µm × 200 µm | 88.3 | – | 8.8 | 0.8 | 1.3 | 0.8 Al | 100 | – |
| IMJ 34341, reverse, SA 1: 100 µm × 100 µm | 88.8 | – | 8.2 | 0.8 | 1.4 | 0.8 Al | 100 | – |
| IMJ 34341, reverse, SA 2: 100 µm × 100 µm | 88.0 | – | 8.8 | 1.0 | 1.5 | 0.7 Al | 100 | – |
| IMJ 34341, reverse, SA 3: 100 µm × 100 µm | 88.2 | – | 9.0 | 0.7 | 1.2 | 0.9 Al | 100 | – |
| IMJ 34421, obverse, SA 1: 200 µm × 200 µm | 94.5 | – | 4.9 | – | 0.6 | – | 100 | – |
| IMJ 34421, obverse, SA 2: 400 µm × 400 µm | 95.5 | – | 4.0 | – | 0.5 | – | 100 | – |
| IMJ 34421, obverse, SA 3: 200 µm × 200 µm | 94.5 | – | 4.7 | – | 0.8 | – | 100 | – |
| IMJ 34421, reverse, SA 4: 100 µm × 100 µm | 95.1 | – | 4.0 | 0.3 | 0.6 | – | 100 | – |
| IMJ 34421, reverse, SA 5: 100 µm × 100 µm | 96.4 | – | 3.2 | – | 0.4 | – | 100 | – |
| IMJ 34421, reverse, SA 6: 100 µm × 100 µm | 95.6 | – | 4.2 | – | 0.3 | – | 100 | – |
| IMJ 34424, obverse, SA 1 (poor state of preservation): 400 µm × 400 µm | – | 36.0 | 36.9 | 19.4 | 0.7 | 2.1 Al, 1.3 K, 1.9 Ca, 1.7 Fe | – | 100 |
| IMJ 34424, obverse, SA 2 (poor state of preservation): 400 µm × 400 µm | 0.8 | 29.2 | 40.1 | 20.0 | 0.4 | 3.9 Al, 1.2 Mg, 0.6 P, 1.7 K, 1.8 Fe | 2.7 | 97.3 |
| IMJ 34424, obverse, SA 3 (poor state of preservation): 200 µm × 200 µm | 3.0 | 33.3 | 37.0 | 15.2 | 1.2 | 1.9 Al, 0.4 S, 1.0 K 2.0 Fe | 8.3 | 91.7 |
| IMJ 34424, reverse, SA 1 (poor state of preservation): 200 µm × 200 µm | 1.1 | 41.9 | 33.7 | 18.4 | 0.3 | 1.4 Al, 1.0 K, 1.2 Ca, 1.0 Fe | 2.6 | 97.4 |
| IMJ 34424, reverse, SA 2 (poor state of preservation): 100 µm × 100 µm | 2.2 | 37.2 | 37.4 | 11.1 | 2.9 | 1.3 Al, 1.4 P, 0.5 S, 0.9 K, 3.0 Ca, 2.1 Fe | 5.6 | 94.4 |
| IMJ 34424, reverse, SA 3: 1000 µm × 1000 µm | 1.2 | 32.4 | 36.2 | 17.8 | 0.6 | 3.1 Al, 1.6 K, 4.9 Ca, 2.2 Fe | 3.6 | 96.4 |
| IMJ 34487, obverse, SA 1: 100 µm × 100 µm | 71.4 | 4.0 | 5.5 | 0.3 | 16.6 | 2.2 S | 94.4 | 5.6 |

| | | | | | | | | |
|---|---|---|---|---|---|---|---|---|
| IMJ 34487, obverse, SA 2: 100 µm × 100 µm | 72.7 | 2.3 | 5.6 | – | 18.6 | 0.8 S | 96.8 | 3.1 |
| IMJ 34487, obverse, SA 3: 100 µm × 100 µm | 70.6 | 4.3 | 4.5 | – | 17.5 | 1.0 Al, 2.1 S | 94.3 | 5.7 |
| IMJ 34487, reverse, SA 1: 100 µm × 100 µm | 72.0 | 1.1 | 4.7 | 0.5 | 20.3 | 1.0 Al, 0.4 S | 98.5 | 1.5 |
| IMJ 34487, reverse, SA 2: 300 µm × 300 µm | 69.7 | 4.6 | 6.8 | 0.3 | 16.6 | 2.0 S | 93.5 | 6.5 |
| IMJ 34487, reverse, SA 3: 100 µm × 100 µm | 70.8 | – | 8.1 | 0.5 | 20.0 | 0.6 Ca | 100 | – |
| IMJ 34503, obverse, SA 1: 200 µm × 200 µm | 91.1 | 0.8 | 6.7 | – | 0.5 | 0.9 Au | 99.1 | 0.9 |
| IMJ 34503, obverse, SA 2: 100 µm × 100 µm | 91.0 | – | 7.5 | – | 0.5 | 1.0 Au | 100 | – |
| IMJ 34503, obverse, SA 3: 200 µm × 200 µm | 91.0 | – | 6.6 | 0.3 | 0.6 | 1.5 Au | 100 | – |
| IMJ 34503, reverse, SA 1: 400 µm × 400 µm | 83.5 | – | 13.9 | 0.6 | 0.7 | 0.5 Na, 0.4 Ca 0.4 Al | 100 | – |
| IMJ 34503, reverse, SA 2: 100 µm × 100 µm | 91.7 | – | 7.5 | – | 0.8 | – | 100 | – |
| IMJ 34503, reverse, SA 3: 100 µm × 100 µm | 87.6 | – | 10.3 | 0.6 | 1.5 | – | 100 | – |

**2. Characterization of the Silver Alloy Jewelry from the Samarian Hacksilber Hoard**

The description of the silver jewelry (samples 1–14, Fig. S1) from the hacksilber hoard according to VT inspection is presented here in the Table S14.

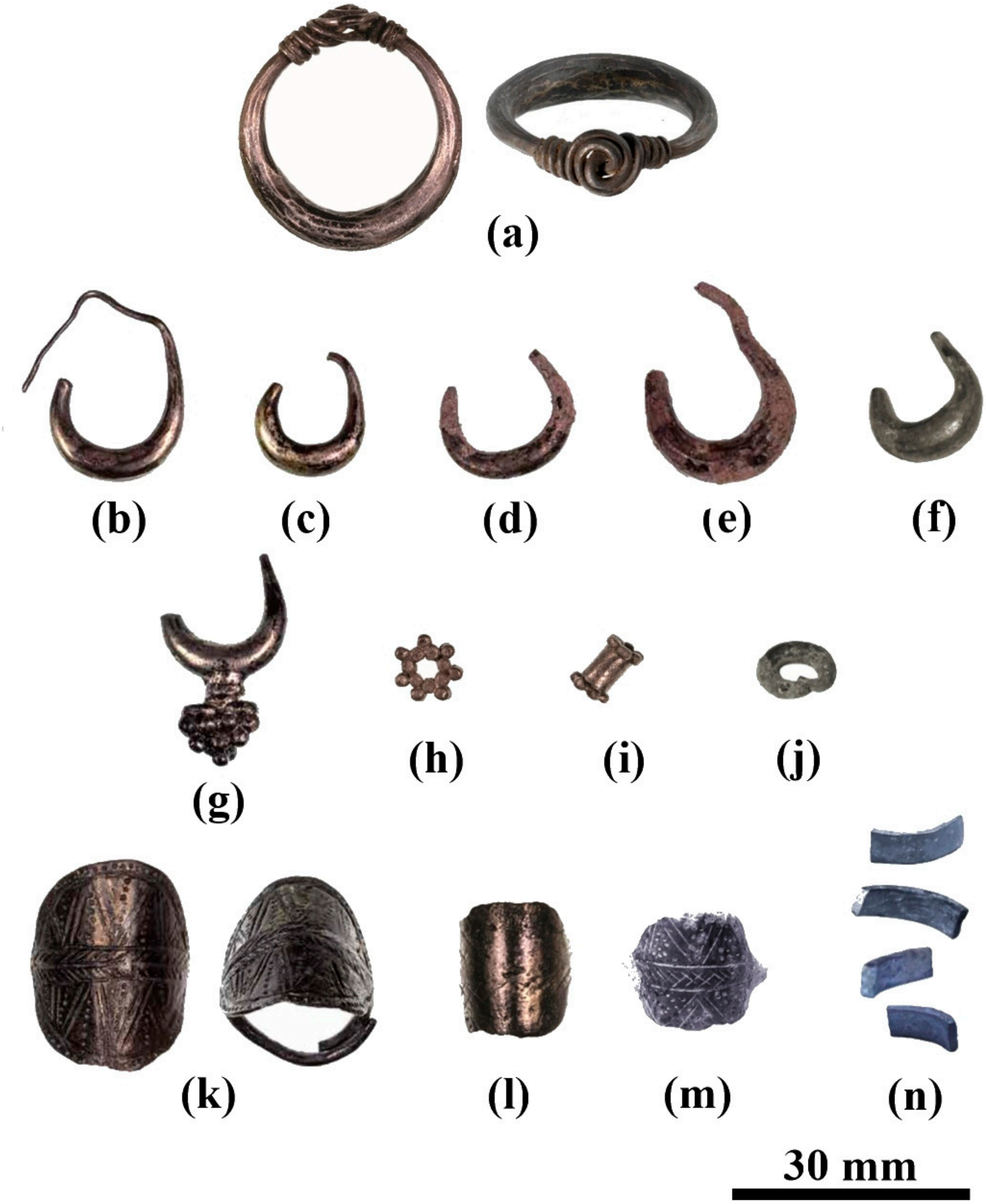


**Figure S1.** The silver jewelry, hacksilber and coins from the hacksilber hoard: (a) Earring/ring (Sample 1) decorated with a flower like rod; (b)–(f) lunate earrings (Samples 2–6); (g) lunate earring decorated with granules (Sample 7); (h) bead made of granules (Sample 8); (i) cylindrical bead decorated with granules (Sample 9); (j) spiral bead (Sample 10); (k)–(m) rings with decorated oval bezel (Samples 11–13), (n) four parts of a broken ring (Sample 14).

VT inspection of the ornamented ring/earring's external surface (Fig. S1a, Sample 1) revealed a well-preserved metallic bright item decorated with a flower like rod. The ornamented ring/earring was cast into a circular/ring shape and then the part was decorated by twisting and plastically deforming a bar with 1 mm cross-section into a flower, as shown by the SEM observation (Fig. S2).

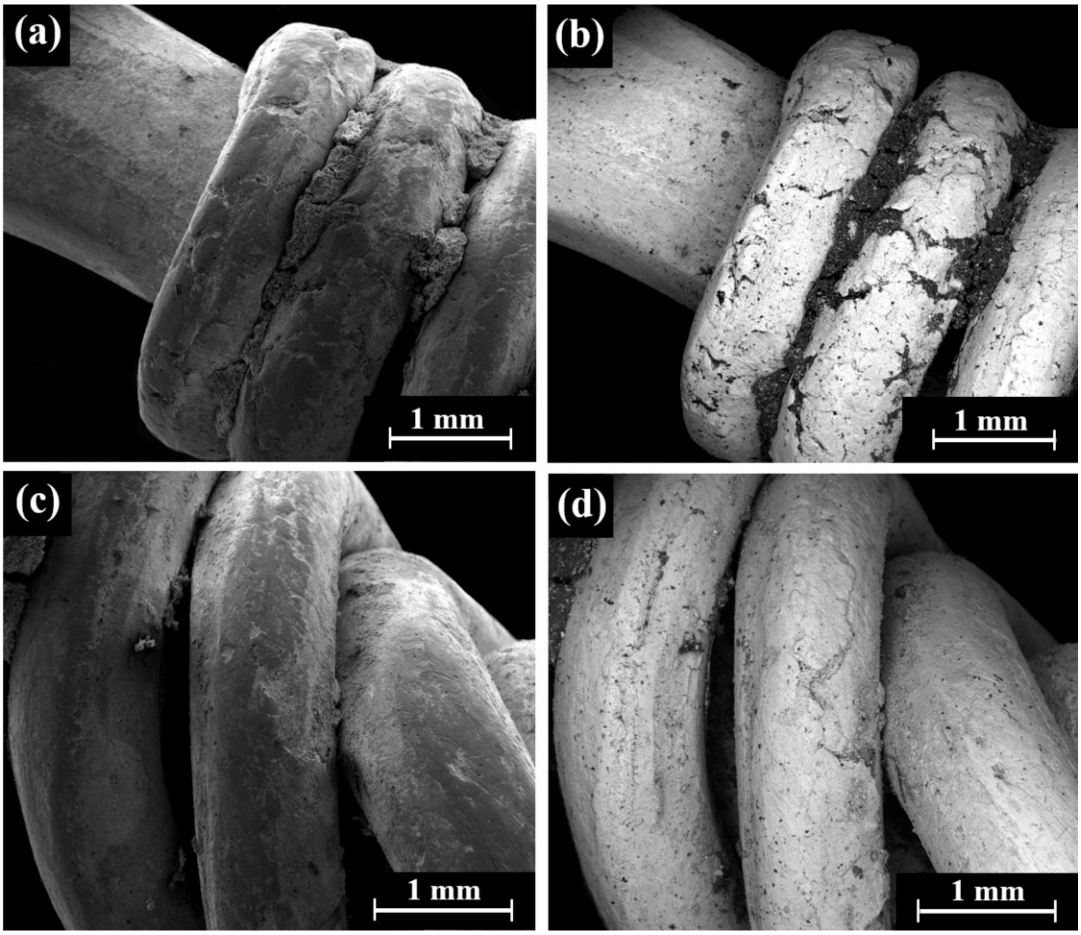


**Figure S2.** SEM images of the ornamented earring (or ring), Sample 1: (a)–(b) the item's cast bar surrounded by a rod shaped into a flower (SE mode and BSE mode, respectively); and (c)–(d) the rod that was wrapped around the item in order to decorate the ornamented earring or ring (SE mode and BSE mode, respectively). The well-preserved bright areas (according to BSE mode) were examined by SEM-EDS analysis.

SEM-EDS analysis of the item (earring or ring, Sample 1) revealed it was made of a ternary silver-copper-gold alloy; yet, presence of the other elements was also detected, including O, Si, Cl, Al, and Fe (Table S14). The alloy composition (after omitting the peaks of O, Si, Cl, Al, and Fe) was 93.9–95.9 wt% Ag, 1.2–2.7 wt% Cu, and 2.9–3.5 wt% Au (Table S14).

VT inspection of the lunate earrings (Fig. S1b–g, Samples 2–7), among them six broken items with tear edges cut (Samples 3–7), revealed a well-preserved metallic bright external surface. These earrings were cast into their general form and next were worked to their final shape, where the diameter of the edges of each earring was reduced by plastic deformation. One of the lunate earrings was decorated with granules (Sample 7, Fig. S1g). This decorated lunate earring included two silver loops which were formed from a bent rod, each with an external diameter of 5–6 mm and cross-section with diameter of 1.5 mm. These loops were then brazed to the lower part of the earring (Sample 7, Fig. S1g). Fifteen granules were created from a rod that was bent into a 15-layer spiral and then sawed into 15 loops of equal size. Each of the loops was then melted in order to transform it into a liquid sphere due to surface

tension considerations, where each small silver loop was shrunk to the minimum surface area according to cohesive forces between liquid atoms. When the fire was removed from the liquid silver spheres, these spheres were transformed into solid state granules, 3–4 mm diameter each.

SEM-EDS analysis of the lunate earrings revealed that they were made of several alloys, including a binary silver-gold alloy in the case of Sample 2 and Sample 4 (with an average alloy composition of 93.4±6.6 wt% Ag and 6.6±6.6 wt% Au), binary silver-copper alloy in the case of Sample 5 and Sample 6 (with an average alloy composition of 93.5±6.7 wt% Ag, 5.5±6.8 wt% Cu and only 1.0±2.3 wt% Au, where gold was detected in only two of the seven examined areas of these samples), and ternary silver-copper alloy in the case of Sample 3 (84.5 wt% Ag, 12.0 wt% Cu and 3.5 wt% Au). Existence of other elements was also detected during the analysis of the lunate earrings, including the presence of O, Si, Cl, Al, and Fe (Table S14). The decorated lunate earring (Sample 7) was made of a ternary silver-copper alloy with higher concentration of gold than the other lunate earrings, with a composition of 64.8–72.9 wt% Ag, 0.7–1.9 wt% Cu, and 9.2–33.4 wt% Au (after omitting the peaks of oxides, corrosion products, and soil elements, Table S14).

**Table S14.** SEM-EDS analysis results of silver jewelry, where SA represents the scanned area.

| Sample | Composition (wt %) | | | | | | | | | |
|---|---|---|---|---|---|---|---|---|---|---|
| | Surface | | | | | | | Silver alloy | | |
| | Ag | Cu | Au | O | Si | Cl | Others | Ag | Cu | Au |
| Sample 1 (large ring), SA 1: 400 µm × 400 µm | 82.2 | 1.1 | 2.5 | 7.3 | 0.6 | 5.1 | 0.5 Al, 0.6 Fe | 95.8 | 1.3 | 2.9 |
| Sample 1, SA 2: 200 µm × 200 µm | 82.7 | 1.0 | 2.5 | 9.1 | 0.6 | 3.8 | 0.3 Al | 95.9 | 1.2 | 2.9 |
| Sample 1, SA 3: 500 µm × 500 µm | 73.3 | 2.1 | 2.7 | 9.9 | 0.4 | 11.7 | – | 93.9 | 2.7 | 3.5 |
| Sample 1, SA 4: 200 µm × 200 µm | 78.0 | 1.7 | 2.6 | 9.9 | 0.6 | 6.9 | 0.4 Al | 94.8 | 2.1 | 3.2 |
| Sample 2 (lunate earring), SA 1: 500 µm × 500 µm | 64.0 | – | 15.7 | 5.2 | – | 14.1 | 1.0 Al | 80.3 | – | 19.7 |
| Sample 2, SA 2: 300 µm × 300 µm | 70.3 | – | 2.3 | 6.6 | 0.5 | 19.5 | 0.9 Al | 96.8 | – | 3.2 |
| Sample 3 (lunate earring), SA 1: 500 µm × 500 µm | 68.2 | 8.5 | 3.1 | 14.3 | – | 5.5 | 0.3 Al | 85.4 | 10.7 | 3.9 |
| Sample 3, SA 2: 300 µm × 300 µm | 65.5 | 10.3 | 2.5 | 14.1 | 0.4 | 7.3 | – | 83.6 | 13.2 | 3.2 |
| Sample 4 (lunate earring), SA 1: 200 µm × 200 µm | 69.2 | – | 2.3 | 0.6 | 2.2 | 15.2 | 0.9 Al, 0.7 S | 96.8 | – | 3.2 |
| Sample 4, SA 2: 300 µm × 300 µm | 69.5 | – | 1.3 | 9.7 | 1.3 | 16.0 | 1.6 Al, 0.6 S | 98.2 | – | 1.8 |
| Sample 4, SA 3: 300 µm × 300 µm | 66.1 | – | 3.8 | 10.8 | 3.3 | 14.3 | 1.0 Al, 0.7 S | 94.6 | – | 5.4 |
| Sample 5 (lunate earring), SA 1: 300 µm × 300 µm | 60.7 | 2.6 | 4.1 | 16.9 | 1.9 | 13.3 | 0.4 Al | 90.1 | 3.9 | 6.1 |
| Sample 5, SA 2: 400 µm × 400 µm | 47.9 | 12.8 | – | 23.3 | 1.5 | 14.0 | 0.5 Ca | 78.9 | 21.1 | – |
| Sample 5, SA 3: 200 µm × 200 µm | 67.2 | – | – | 11.7 | 1.7 | 18.1 | 1.3 Al | 100 | – | – |
| Sample 6 (lunate earring), SA 1: 100 µm × 100 µm | 62.2 | 3.0 | – | 14.4 | 1.0 | 18.4 | 1.0 Al | 95.4 | 4.6 | – |

| | | | | | | | | | | |
|---|---|---|---|---|---|---|---|---|---|---|
| Sample 6 (crystalline area), SA 2: 20 μm × 20 μm | 63.9 | 3.1 | – | 12.4 | 0.5 | 19.0 | 1.0 Al | 95.4 | 4.6 | – |
| Sample 6, SA 3: 20 μm × 20 μm | 70.8 | – | – | 7.0 | 0.5 | 20.8 | 1.0 Al | 100 | – | – |
| Sample 6, SA 4: 100 μm × 100 μm | 65.5 | 1.0 | 2.7 | 12.3 | 1.1 | 16.6 | 0.8 al | 94.7 | 1.4 | 3.9 |
| Sample 7 (decorated lunate earring), SA 1: 300 μm × 300 μm | 55.9 | 1.5 | 28.8 | 5.9 | – | 8.0 | – | 64.8 | 1.7 | 33.4 |
| Sample 7, SA 2: 100 μm × 100 μm | 68.6 | 0.5 | 7.0 | 6.6 | 0.5 | 14.8 | 1.0 Al, 1.1 S | 90.1 | 0.7 | 9.2 |
| Sample 7, SA 3: 200 μm × 200 μm | 57.8 | 1.5 | 20.0 | 7.7 | 0.5 | 12.2 | 0.6 Al | 72.9 | 1.9 | 25.2 |
| Sample 8 (bead made of granules), SA 1: 200 μm × 200 μm | 75.4 | – | – | 8.7 | 0.8 | 12.2 | 1.2 Al, 0.3 S, 0.7 Ca, 0.7 Fe | 100 | – | – |
| Sample 8, granule, SA 2: 100 μm × 100 μm | 72.3 | – | – | 9.1 | 0.8 | 14.1 | 1.0 Al, 0.9 Ca | 100 | – | – |
| Sample 8, granule, SA 3: 200 μm × 200 μm | 78.6 | – | – | 7.1 | 0.7 | 12.2 | 0.9 Al, 0.6 Ca | 100 | – | – |
| Sample 8, joint between granules, SA 4: 100 μm × 100 μm | 61.2 | – | – | 20.6 | 2.5 | 10.1 | 2.4 Al, 0.3 S, 2.3 Ca, 0.6 Fe | 100 | – | – |
| Sample 8, joint between granules, SA 5: 300 μm × 300 μm | 69.4 | – | – | 23.4 | 1.8 | 0.5 | 1.1 Al, 0.4 P, 0.3 S, 2.4 Ca, 0.7 Fe | 100 | – | – |
| Sample 8, joint between granules), SA 6: 300 μm × 300 μm | 56.4 | – | – | 5.0 | 8.7 | – | 0.8 Mg, 3.7 Al, 1.0 P, 0.6 K, 21.5 Ca, 2.3 Fe | 100 | – | – |
| Sample 8, joint between granules, SA 7: 100 μm × 100 μm | 68.4 | – | – | 24.6 | 2.0 | 1.1 | 1.3 Al, 0.4 S, 2.1 Ca | 100 | – | – |
| Sample 8, granule, SA 8: 100 μm × 100 μm | 67.4 | – | – | 20.6 | 1.6 | 6.6 | 1.1 Al, 0.4 S, 2.4 Ca | 100 | – | – |
| Sample 8, joint between granules, SA 9: 100 μm × 100 μm | 64.0 | – | – | 20.3 | 1.9 | 9.9 | 2.2 Al, 1.6 Ca | 100 | – | – |
| Sample 9 (cylindrical bead decorated with granules), SA 1: 200 μm × 200 μm | 72.7 | – | – | 11.3 | 0.8 | 10.8 | 2.8 Al, 0.8 S, 0.9 Ca | 100 | – | – |
| Sample 9, SA 2: 100 μm × 100 μm | 74.5 | – | – | 9.4 | 0.5 | 10.4 | 3.9 Al, 0.6 S, 0.6 Ca | 100 | – | – |
| Sample 9, folding, SA 3: 100 μm × 100 μm | 73.3 | – | – | 21.4 | 1.5 | 0.6 | 0.9 Al, 1.1 S, 1.2 Ca | 100 | – | – |
| Sample 9, joint between granule and surface, SA 4: 200 μm × 200 μm | 77.3 | 1.3 | – | 15.9 | 1.0 | 1.3 | 0.7 Al, 1.2 S, 1.2 Ca | 98.3 | 1.7 | – |

| | | | | | | | | | | |
|---|---|---|---|---|---|---|---|---|---|---|
| Sample 9, joint between granules, SA 5: 100 µm × 100 µm | 59.2 | – | – | 26.7 | 1.9 | 4.7 | 2.6 Al, 0.9 S, 3.2 Ca, 0.7 Fe | 100 | – | – |
| Sample 9, joint between granules, SA 6: 100 µm × 100 µm | 71.3 | – | – | 14.2 | 0.7 | 8.7 | 2.9 Al, 0.9 S, 1.3 Ca | 100 | – | – |
| Sample 9, granule with dendrites, SA 7: 500 µm × 500 µm | 60.2 | 1.1 | – | 26.3 | 2.0 | 2.0 | 1.4 Al, 0.5 S, 4.2 Ca, 0.6 Fe, 1.7 Pb | 98.2 | 1.8 | – |
| Sample 9, granule with dendrites, SA 8: 400 µm × 400 µm | 61.1 | 0.9 | – | 27.5 | 2.1 | 2.2 | 1.2 Al, 0.4 S, 4.0 Ca, 0.6 Fe | 98.5 | 1.5 | – |
| Sample 9, joint between granules, SA 9: 100 µm × 100 µm | 57.4 | – | – | 26.6 | 1.8 | 5.4 | 2.0 Al, 0.3 P, 0.7 S, 5.3 Ca, 0.5 Fe | 100 | – | – |
| Sample 9, joint between granules, SA 10: 100 µm × 100 µm | 75.0 | – | – | 18.8 | 1.5 | 0.4 | 0.6 Al, 2.4 S, 0.4 Cl | 100 | – | – |
| Sample 9, joint between granules, SA 11: 100 µm × 100 µm | 83.3 | 0.9 | – | 11.8 | 0.9 | 0.6 | 0.4 Al, 1.1 S, 0.9 Ca | 98.5 | 1.5 | – |
| Sample 9, joint between granules, SA 12: 100 µm × 100 µm | 79.5 | – | – | 15.4 | 1.0 | 0.7 | 0.6 Al, 0.7 S, 2.1 Ca | 100 | – | – |
| Sample 9, joint between granules, SA: 2 µm × 2 µm | 87.5 | 1.1 | – | 9.7 | 0.6 | 0.3 | 0.4 Al, 0.3 S | 98.8 | 1.2 | – |
| Sample 10 (circular/spiral bead), SA 1: 200 µm × 200 µm | 49.4 | 10.2 | 1.1 | 21.1 | 2.9 | 14.8 | 0.5 Al | 81.4 | 16.8 | 1.8 |
| Sample 10, SA 2: 200 µm × 200 µm | 64.2 | 1.4 | 2.0 | 12.1 | 3.1 | 17.2 | – | 95.0 | 2.1 | 3.0 |
| Sample 10, SA 3: 300 µm × 300 µm | 46.2 | 4.8 | – | 28.5 | 4.7 | 14.7 | 0.6 Al, 0.6 Ca | 90.6 | 9.4 | – |
| Sample 10, SA 4: 200 µm × 200 µm | 54.4 | 2.1 | – | 24.6 | 2.5 | 15.7 | 0.5 Al, 0.4 Ca | 96.3 | 3.7 | – |
| Sample 11 (decorated ring), SA 1 (surface with oxide): 100 µm × 100 µm | 62.0 | 1.5 | 5.2 | 15.4 | 4.0 | 10.7 | 0.5 Al, 0.6 Ca | 90.2 | 2.2 | 7.6 |
| Sample 11, SA 2: 200 µm × 200 µm | 65.2 | 1.4 | 5.0 | 11.6 | 3.6 | 12.8 | 0.5 Al | 91.1 | 2.0 | 7.0 |
| Sample 11, SA 3 (surface with oxide): 200 µm × 200 µm | 64.4 | 1.3 | 3.6 | 13.9 | 3.4 | 12.7 | 12.7 Cl, 0.7 Ca | 92.9 | 1.9 | 5.2 |
| Sample 11, SA 4 (fractured area of exposed metal): 100 µm × 100 µm | 92.8 | 4.9 | 2.3 | – | – | – | – | 92.8 | 4.9 | 2.3 |
| Sample 11, SA 5 (fractured area of exposed metal): 100 µm × 100 µm | 93.1 | 4.6 | 2.3 | – | – | – | – | 93.1 | 4.6 | 2.3 |

| | | | | | | | | | | |
|---|---|---|---|---|---|---|---|---|---|---|
| Sample 11, SA 6 (fractured area of exposed metal): 200 µm × 200 µm | 93.2 | 4.4 | 2.4 | – | – | – | – | 93.2 | 4.4 | 2.4 |
| Sample 12 (decorated ring), SA 1 (fractured surface with exposed metal): 100 µm × 100 µm | 89.8 | 4.4 | 2.2 | 2.9 | – | 0.7 | – | 93.2 | 4.6 | 2.3 |
| Sample 12, SA 2 (area with corrosion: 20 µm × 20 µm | 22.4 | 0.7 | 0.9 | 20.5 | 2.4 | 4.3 | 45.9 C, 0.3 Mg, 0.8 Al, 0.7 K, 1.0 Ca | 93.3 | 2.9 | 3.8 |
| Sample 12, SA 3 (rough surface): 20 µm × 20 µm | 76.0 | 2.2 | 3.6 | 7.3 | 0.6 | 10.0 | 0.4 Al | 92.9 | 2.7 | 4.4 |
| Sample 12, SA 4: 50 µm × 50 µm | 90.0 | 3.8 | 2.3 | 3.3 | – | 0.7 | – | 93.7 | 4.0 | 2.4 |
| Sample 12, SA 4 (fractured surface with exposed metal): 10 µm × 10 µm | 90.8 | 5.7 | 2.7 | – | – | 0.8 | – | 91.5 | 5.7 | 2.7 |
| Sample 12, SA 5 (surface covered with oxide): 300 µm × 300 µm | 66.4 | 2.9 | 8.4 | 9.8 | 4.1 | 8.4 | – | 85.5 | 3.7 | 10.8 |
| Sample 12, SA 6 (surface covered with oxide): 500 µm × 500 µm | 66.3 | 3.2 | 6.5 | 10.7 | 4.4 | 8.9 | – | 87.2 | 4.2 | 8.6 |
| Sample 12, SA 7: 300 µm × 300 µm | 68.3 | 3.1 | 6.0 | 10.3 | 3.5 | 8.8 | – | 88.2 | 4.0 | 7.8 |
| Sample 13 (decorated ring), SA 1 (fractured area of exposed metal): 100 µm × 100 µm | 90.0 | 4.0 | 1.7 | 3.3 | – | 1.0 | – | 94.0 | 4.2 | 1.8 |
| Sample 13, SA 2: 100 µm × 100 µm | 89.6 | 4.0 | 2.2 | 2.9 | – | 1.53 | – | 93.5 | 4.2 | 2.3 |
| Sample 13, SA 3 (fractured area of exposed metal): 100 µm × 100 µm | 88.9 | 4.7 | 2.4 | 2.6 | – | 1.5 | – | 92.6 | 4.9 | 2.5 |
| Sample 13, SA 4 (surface covered with oxide): 500 µm × 500 µm | 74.5 | 1.4 | 4.6 | 5.3 | 1.5 | 12.7 | – | 92.5 | 1.7 | 5.7 |
| Sample 13, SA 5 (bright phase): 50 µm × 50 µm | 85.4 | 2.4 | 2.9 | 3.4 | 0.6 | 5.3 | – | 94.2 | 2.6 | 3.2 |
| Sample 13, SA 6 (dark phase): 50 µm × 50 µm | 72.3 | 1.0 | 4.5 | 3.5 | 1.2 | 17.7 | – | 92.9 | 1.3 | 5.8 |
| Sample 14 (broken ring fragments), SA 1: 500 µm × 500 µm | 83.5 | – | 2.7 | 3.0 | 1.1 | 9.4 | 0.4 Al | 96.9 | – | 3.1 |
| Sample 14, SA 2: 400 µm × 400 µm | 85.6 | – | 2.1 | 3.4 | 0.7 | 7.8 | 0.5 Al | 97.6 | – | 2.4 |
| Sample 14, SA 3 (fractured area of exposed metal): 400 µm × 400 µm | 87.5 | 3.9 | 2.1 | 4.7 | – | 1.9 | – | 93.6 | 4.2 | 2.2 |

| | | | | | | | | | | |
|---|---|---|---|---|---|---|---|---|---|---|
| Sample 14, SA 4 (fractured area of exposed metal): 100 µm × 100 µm | 86.6 | 3.5 | 1.3 | 5.3 | – | 3.3 | – | 94.7 | 3.8 | 1.4 |
| Sample 14, SA 5 (fractured area of exposed metal): 300 µm × 300 µm | 83.2 | 1.6 | 3.6 | 3.9 | 0.8 | 6.4 | 0.5 Al | 94.1 | 1.8 | 4.1 |
| Sample 14, SA 6 (fractured area of exposed metal): 500 µm × 500 µm | 81.3 | 1.7 | 3.5 | 4.3 | 1.2 | 7.5 | 0.5 Al | 94.0 | 2.0 | 4.0 |
| Sample 14, SA 7: 200 µm × 200 µm | 66.2 | – | 1.1 | 12.9 | 2.1 | 15.5 | 2.2 Al | 98.4 | – | 1.6 |
| Sample 14, SA 8: 200 µm × 200 µm | 68.7 | – | 1.4 | 9.3 | 1.6 | 16.8 | 2.2 Al | 98.0 | – | 2.0 |
| Sample 14, SA 9: 200 µm × 200 µm | 71.6 | – | 6.1 | 13.4 | 1.7 | 6.6 | 0.6 Al | 92.1 | – | 7.9 |
| Sample 14, SA 10 (surface covered with oxide): 300 µm × 300 µm | 66.2 | – | – | 13.6 | 2.5 | 16.3 | 1.5 Al | 100 | – | – |
| Sample 14, SA 11: 200 µm × 200 µm | 65.8 | – | – | 14.0 | 1.7 | 16.4 | 2.1 Al | 100 | – | – |
| Sample 14, SA 12 (surface covered with oxide): 200 µm × 200 µm | 70.0 | – | – | 9.1 | 1.0 | 18.4 | 1.5 Al | 100 | – | – |
| Sample 14, SA 13: 300 µm × 300 µm | 66.2 | – | 1.7 | 12.1 | 2.3 | 15.8 | 2.0 Al | 97.5 | 1.7 | 2.5 |
| Sample 14, SA 14: 200 µm × 200 µm | 67.3 | – | – | 11.2 | 1.5 | 17.1 | 2.8 Al | 100 | – | – |
| Sample 14, SA 15: 200 µm × 200 µm | 70.3 | – | 3.5 | 12.5 | 2.2 | 7.8 | 1.0 Al | 95.3 | – | 4.7 |
| Sample 14, SA 16: 200 µm × 200 µm | 89.7 | 4.0 | 1.8 | 2.8 | – | 1.8 | – | 93.9 | 4.2 | 1.9 |
| Sample 14, SA 17 (fractured area with exposed metal): 100 µm × 100 µm | 91.3 | 4.5 | 2.7 | – | – | 1.8 | – | 92.7 | 4.6 | 2.7 |
| Sample 14, SA 18 (fractured area with exposed metal): 100 µm × 100 µm | 75.9 | 1.4 | 1.1 | 17.1 | 0.7 | 3.3 | 0.6 Ca | 94.5 | 3.1 | 2.4 |
| Sample 14, SA 19 (fractured area with exposed metal): 100 µm × 100 µm | 81.8 | – | 3.9 | 10.9 | 2.2 | 1.2 | – | 95.4 | – | 4.6 |
| Sample 14, SA 20: 100 µm × 100 µm | 78.0 | – | 5.5 | 9.9 | 2.5 | 4.2 | – | 93.4 | – | 6.6 |
| Sample 14, SA 21: 100 µm × 100 µm | 84.2 | – | 3.7 | 9.5 | 1.4 | 1.2 | – | 95.8 | – | 4.2 |

VT inspection of the granulated bead made of two layers of granules (Sample 8) revealed a well-preserved surface with a metallic shiny appearance (Fig. S1h). The original bead was made of 28

granules (14 in the upper layer and 14 in the lower layer); however, a few of the granules of the lower layer were missing. Each of the granules in this two-layered bead were probably created from a thin rod (~ 1 mm diameter) that was bent into a 28-layer spiral and then sliced into similar small loops. Each of the loops was melted and transformed into a liquid sphere. Next the similar spheres were cooled and solid-state granules were formed, each with a ~ 1 mm diameter. Then the granules were brazed together to create the final bead.

SEM observation of the two-layer granulated bead (Sample 8) revealed a rough surface; yet well-preserved (Fig. S3). The bead's granules and the joints between granules were made of silver; yet other elements were also detected during the analysis of this bead surface, including O, Si, Cl, Al, S, Ca, and Fe (Table S14). SEM-EDS analysis results of this bead after omitting the peaks of oxides, corrosion products, and soil elements revealed it was made of pure silver (100 wt% Ag, Table S14).

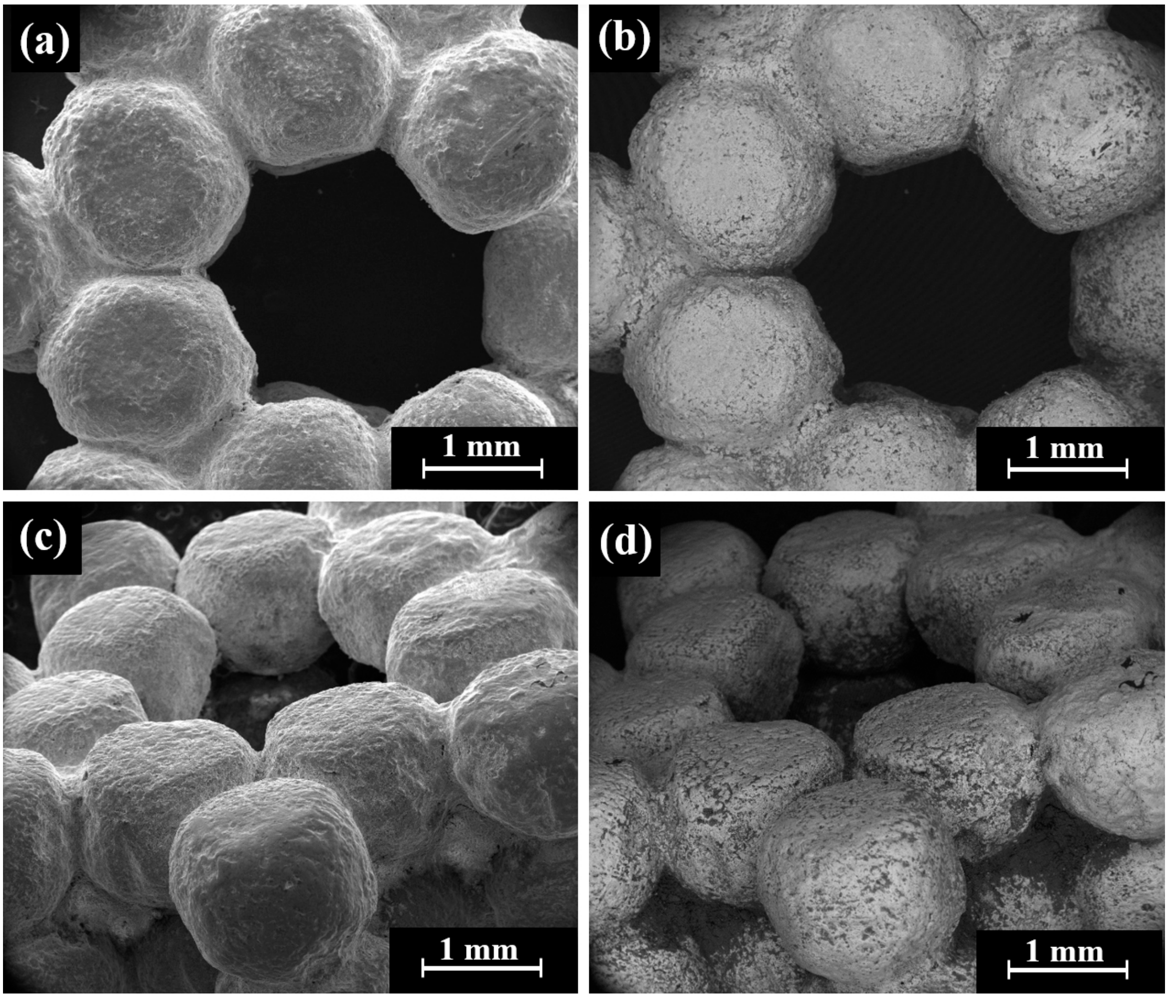


**Figure S3.** SEM images of the two-layer granulated bead (Sample 8): (a)–(b) top view of the upper layer of granules (SE mode and BSE mode, respectively); and (c)–(d) isometric view of the two layers of granules (SE mode and BSE mode, respectively).

VT inspection of the cylindrical bead with edges decorated with six granules in each side (Sample 9) revealed a well-preserved surface (Fig. S1i). SEM observation revealed that the granule surface was rough and included a dendritic texture with dark areas according to BSE mode of corrosion products and soil remains; yet bright areas according to BSE mode were also observed, composed of almost pure silver metal (Fig. S4).

In order to produce the decorated cylindrical bead, a 5 mm × 10 mm sheet was heated and then bent into a cylindrical shape. Next, each of the granules were created, probably from a thin rod (~ 0.8 mm diameter) that was bent into a 12-layer spiral and then sliced into similar small loops. Each of the slices was then melted and transformed into a liquid sphere that was solidified into a tiny granule of ~ 0.8 mm diameter. Then each of the six granules were brazed together to create a single bead and the two granulated beads were brazed to the sides of the cylindrical bead to create the final decorated cylindrical bead.

Based on SEM-EDS analysis, the cylindrical bead granules and joints were made of high purity sliver alloy; yet other elements were also detected, including O, Si, Cl, Al, S, P, Ca, Pb and Fe (Table S14). The average alloy composition of the cylindrical bead granules was 99.7±0.6 wt% Ag and 0.3±0.6 wt% Cu and the average composition of the joints between the granules revealed a similar average composition of 99.6±0.7 wt% Ag and 0.4±0.7 wt% Cu (after omitting the peaks of oxides, corrosion products and soil elements).

VT inspection of the 10 mm diameter circular/spiral bead (Sample 10, Fig. S1j) revealed that its surface was covered with grey-green oxide, corrosion products and soil elements. SEM-EDS analysis of this bead revealed it was created of ternary silver-copper-gold alloy; yet, other elements were also detected, including O, Si, Cl, Al, and Ca (Table S14). The average alloy composition of the circular/spiral bead was 90.8±5.8 wt% Ag, 8.0±5.8 wt% Cu, and 1.2±1.3 wt% Au (after omitting the peaks of oxides, corrosion products and soil elements).

VT inspection of the three oval-shaped decorated bezel rings (Samples 11–13, Fig. S1k–m) revealed that their surface was covered with grey-green oxide and corrosion products. The front part of each ring was created by sawing the desired oval shape from a silver plate. Then, the front part of each ring was decorated by engraving parallel lines and creating dots by plastic deformation. In the final step, a thin silver bar (~ 1.5 mm in diameter) was bent and brazed to the front oval part of each ring to create the final item.

SEM (BSE mode) observation of these three decorated rings (Samples 11–13, Figs. S5, S6) revealed high magnification level of the decorations at the front of the ring (Sample 11, Fig. S5a–b), as well as the microstructure of the rings at the fracture surface (Figs. 12c–d, 13). SEM-EDS analysis result revealed that these rings were composed of silver, copper, and gold; yet, other elements were also detected on the ring surfaces, including O, C, Si, Cl, Al, Ca, Mg, and K (Table S14). Therefore, these rings were produced from ternary silver-copper-gold alloy with average alloy composition of 92.0±2.3 wt% Ag, 3.5±1.2 wt% Cu, and 4.5±2.6 wt% Au (after omitting the peaks of oxides, corrosion products and soil elements).

SEM fractography observation of the ring's fracture surface at the back of Sample 11 (Fig. S5c–d) exposed an intergranular fracture, where the cracks propagated along the grain boundaries. SEM fractography observation of the ring's fracture surface on the back of Sample 12 (Fig. S6c–d) exposed a mixed fracture of a brittle transgranular embrittlement and intergranular embrittlement.

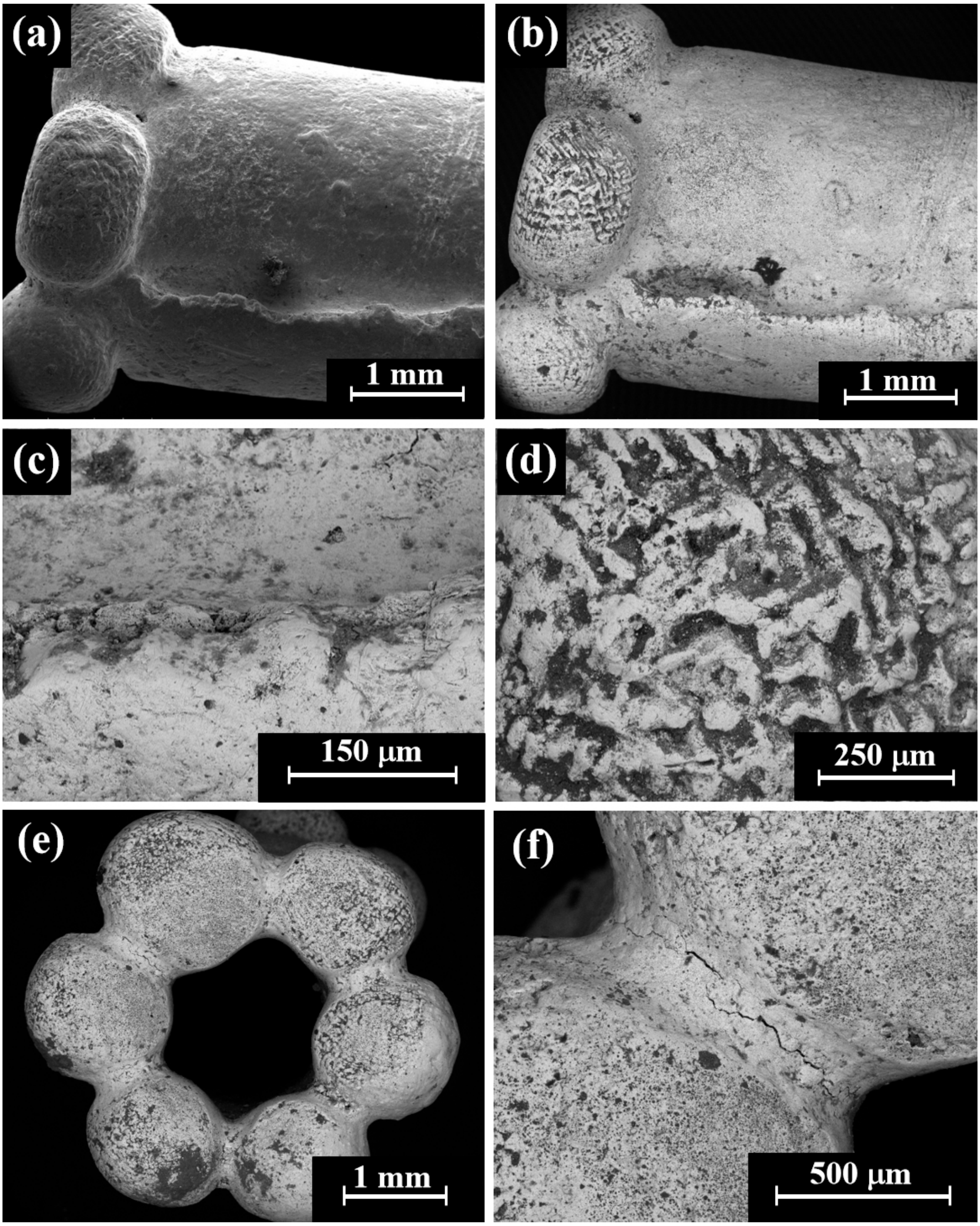


**Figure S4.** SEM images of the cylindrical bead decorated with two granulated beads (Sample 9): (a)–(b) general view left and center parts (SE mode and BSE mode, respectively) of the items; (c) higher magnification of the cylindrical bead joint line; (d) higher magnification of the left granulated bead surface; (e) top view of the left granulated bead; and (f) higher magnification of the joint area between two granules (top view).

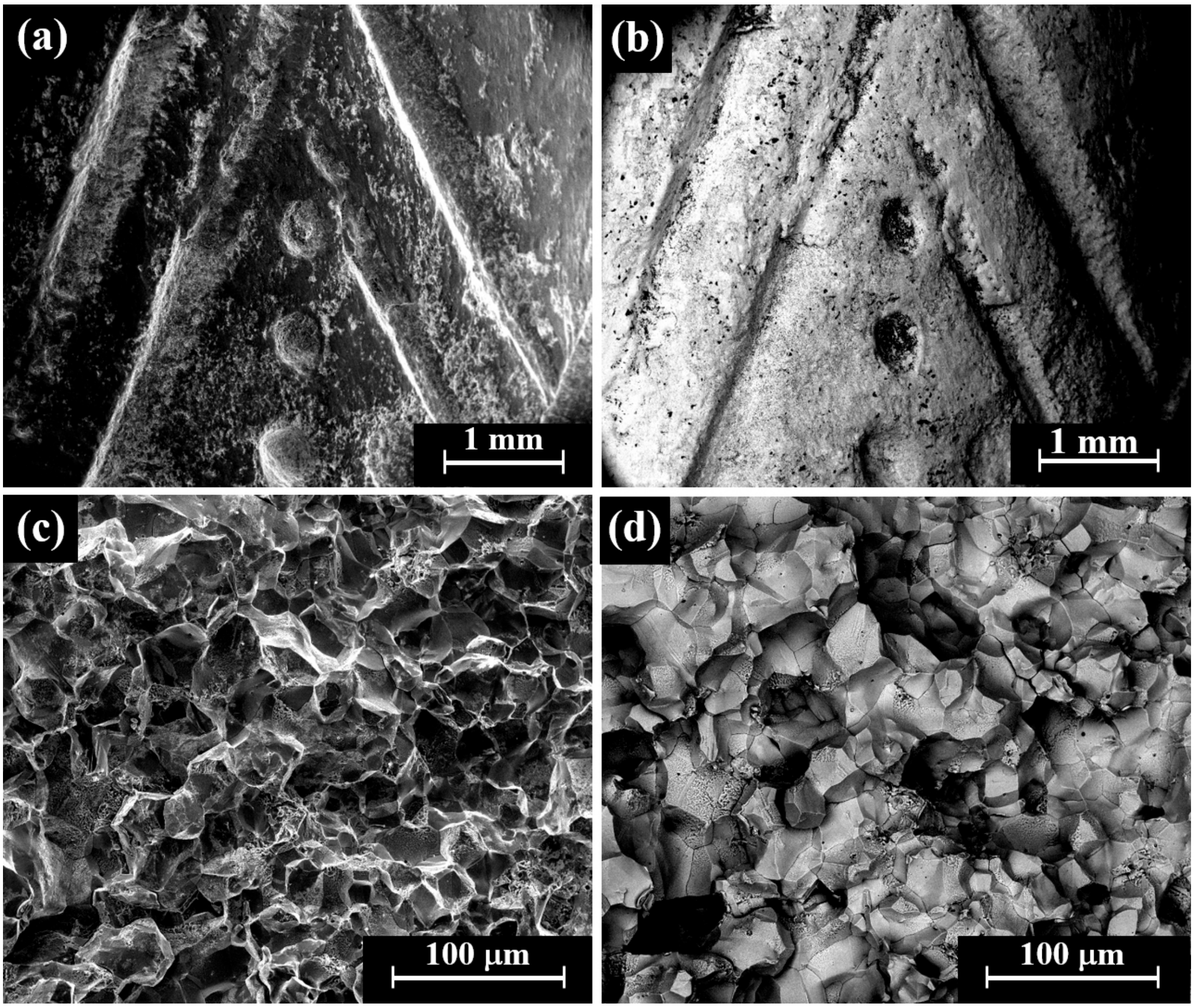


**Figure S5.** SEM images of a ring with a decorated oval front (Sample 11), showing: (a)–(b) the zigzag line and dot decorations at the front of the ring (SE mode and BSE mode, respectively); and (c)–(d) the fracture surface at the broken back of the ring (SE mode and BSE mode, respectively).

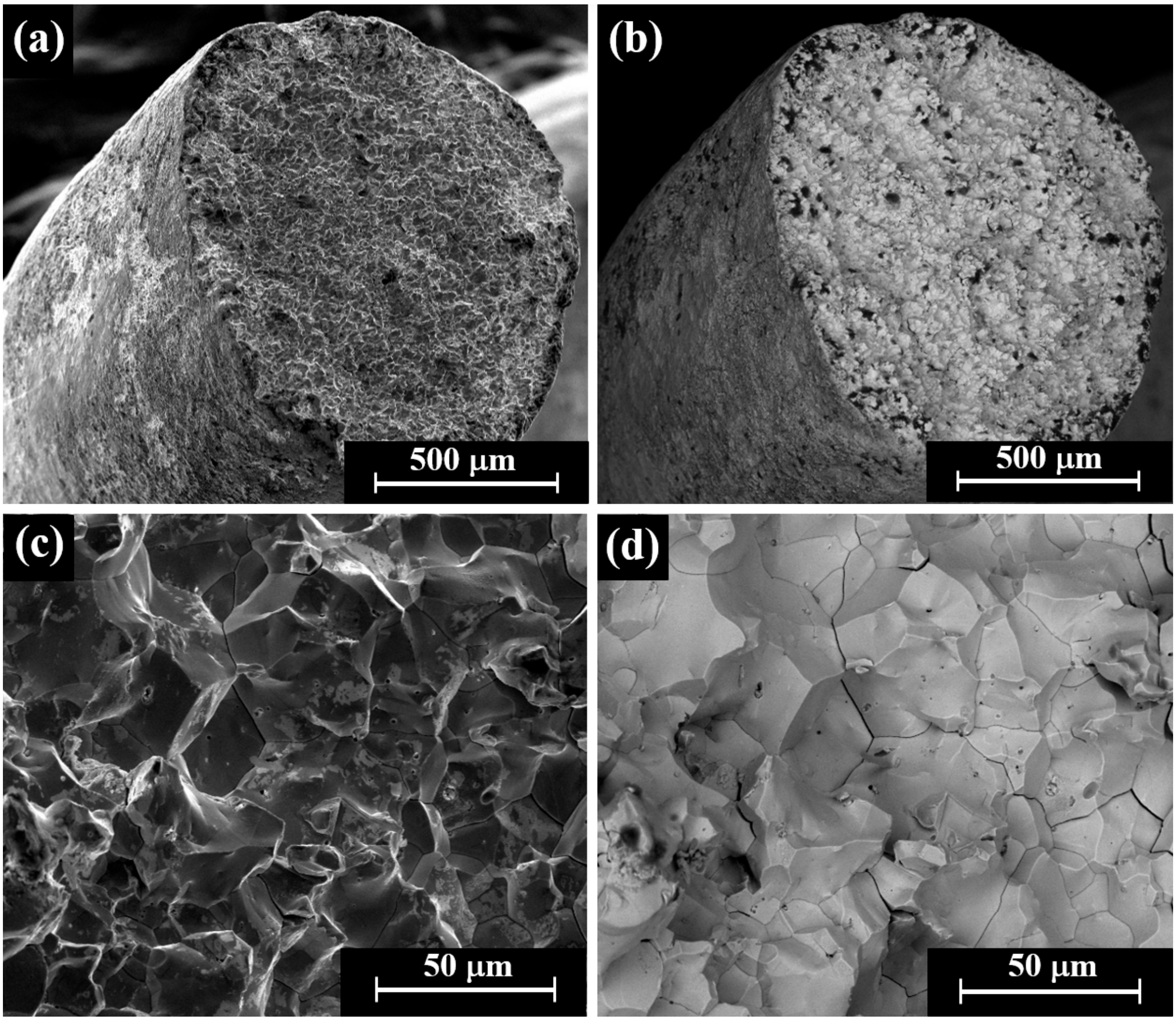


**Figure S6.** SEM images of a ring with oval-shaped decorated bezel (Sample 12), showing: (a)–(b) the fracture surface of the broken back of the ring (SE mode and BSE mode, respectively); and (c)–(d) higher magnification of the fracture surface (SE mode and BSE mode, respectively).

SEM (SE and BSE modes) observations of the broken plain ring fragments (Sample 14; Fig. S1n) revealed that the general surface was well-preserved. In order to create this item, a rectangular strip with a length equal to the desired circumference of the ring was sawed from a thin (around 1 mm thickness) silver plate. Next the rectangular strip was heated to remove residual stresses and then it was bent by plastic deformation to create the ring's shape. The two edges of the ring were next brazed and then the object was shaped into its final form. SEM observation of these ring's fragments (Sample 14) revealed an intergranular fracture surface.

SEM-EDS analysis of the ring fragments (Sample 14) revealed that the total item was produced of a ternary silver-copper-gold alloy; yet, other elements were also detected, including O, C, Si, Cl, Al, and Ca (Table S14). These ring fragments average alloy composition was 95.9±2.6 wt% Ag, 1.2±1.7 wt% Cu, and 2.9±2.1 wt% Au (after omitting the peaks of oxides, corrosion products and soil elements).

EDS analysis of Samples 15–22 (eight fragments, Samples 15–22 and one perhaps striated(?) ingot, Sample 23) from the hacksilber hoard is presented in Table S15, and EDS analysis of the two coins (Sidonian and Philistian, Samples 24–25) is presented in Table S16.

**Table S15.** SEM-EDS analysis results of silver sheets, where SA represents the scanned area.

| Sample | Composition (wt %) | | | | | | | | | |
|---|---|---|---|---|---|---|---|---|---|---|
| | Surface | | | | | | | Silver alloy | | |
| | Ag | Cu | Au | O | Si | Cl | Others | Ag | Cu | Au |
| Sample 15, SA 1: 100 μm × 100 μm | 78.7 | 1.9 | 3.9 | 5.1 | 0.6 | 9.4 | 0.4 Al | 93.2 | 2.2 | 4.6 |
| Sample 15, SA 2: 100 μm × 100 μm | 48.0 | 1.2 | – | 27.4 | 6.8 | 15.9 | 0.8 Al | 97.6 | 2.4 | – |
| Sample 15, SA 3: 200 μm × 200 μm | 73.8 | 1.4 | 2.8 | 15.1 | 2.2 | 4.4 | 0.4 Ca | 94.6 | 1.8 | 3.6 |
| Sample 16, SA 1: 300 μm × 300 μm | 71.4 | 1.2 | 2.0 | 14.5 | 2.1 | 7.9 | 0.4 Al, 0.4 S | 95.7 | 1.6 | 2.7 |
| Sample 16, SA 2: 200 μm × 200 μm | 67.7 | 1.1 | 1.4 | 11.5 | 2.5 | 14.8 | 0.5 Al, 0.5 S | 96.4 | 1.6 | 2.0 |
| Sample 16, SA 3 (corroded area): 400 μm × 400 μm | 46.9 | 3.1 | 0.9 | 30.6 | 6.7 | 10.4 | 0.6 Al, 0.9 Ca | 92.1 | 6.1 | 1.8 |
| Sample 16, SA 4 (corroded area): 100 μm × 100 μm | 24.5 | 1.9 | – | 45.2 | 19.7 | 6.9 | 1.2 Al, 0.7 Ca | 92.8 | 7.2 | – |
| Sample 17, SA 1: 200 μm × 200 μm | 70.0 | 0.9 | 2.1 | 14.6 | 1.6 | 10.1 | 0.9 Al | 95.9 | 1.2 | 2.9 |
| Sample 17, SA 2: 300 μm × 300 μm | 68.3 | 1.0 | 2.5 | 16.7 | 1.9 | 9.1 | 0.7 Al | 95.1 | 1.4 | 3.5 |
| Sample 17, SA 3: 300 μm × 300 μm | 66.6 | – | – | 11.0 | 1.3 | 21.2 | – | 100 | – | – |
| Sample 18, SA 1: 200 μm × 200 μm | 69.4 | – | – | 8.6 | 1.1 | 20.1 | 0.9 Al | 100 | – | – |
| Sample 18, SA 2: 100 μm × 100 μm | 63.5 | 1.7 | 4.6 | 19.4 | 4.5 | 6.4 | – | 91.0 | 2.4 | 6.6 |
| Sample 18, SA 3: 100 μm × 100 μm | 65.8 | – | 1.1 | 11.0 | 2.2 | 18.5 | 1.0 Al, 0.4 S | 98.4 | – | 1.6 |
| Sample 19, SA 1: 200 μm × 200 μm | 66.5 | – | – | 12.0 | 1.8 | 17.9 | 1.5 Al, 0.4 S | 100 | – | – |
| Sample 19, SA 2: 200 μm × 200 μm | 69.9 | – | – | 7.9 | 1.2 | 19.7 | 1.4 Al | 100 | – | – |
| Sample 19, SA 3: 300 μm × 300 μm | 58.3 | – | – | 22.2 | 3.8 | 14.8 | 0.9 Al | 100 | – | – |
| Sample 20, SA 1: 200 μm × 200 μm | 65.1 | 3.2 | – | 10.8 | 0.8 | 20.2 | – | 95.3 | 4.7 | – |
| Sample 20, SA 2: 300 μm × 300 μm | 53.7 | 11.4 | – | 14.8 | 0.7 | 19.0 | 0.4 Al | 82.5 | 17.5 | – |
| Sample 20, SA 3: 200 μm × 200 μm | 61.5 | 5.1 | – | 13.0 | 0.9 | 18.7 | 0.6 Al | 92.3 | 7.7 | – |
| Sample 20, SA 4: 200 μm × 200 μm | 33.5 | 12.3 | – | 32.9 | 5.7 | 13.2 | 0.5 Al, 0.9 Ca | 73.1 | 26.9 | – |
| Sample 21, SA 1: 200 μm × 200 μm | 55.2 | 4.4 | – | 21.9 | 2.1 | 15.1 | 1.3 Al | 92.6 | 7.4 | – |
| Sample 21, SA 2: 100 μm × 100 μm | 57.2 | 3.1 | 1.0 | 10.4 | 2.1 | 15.1 | 1.2 Al | 93.3 | 5.1 | 1.6 |
| Sample 21, SA 3: 100 μm × 100 μm | 62.7 | – | – | 16.2 | 2.0 | 18.3 | 0.3 Al | 100 | – | – |

| | | | | | | | | | | |
|---|---|---|---|---|---|---|---|---|---|---|
| Sample 22, SA 1: 50 µm × 50 µm | 75.1 | – | 1.2 | 4.5 | 0.6 | 17.8 | 0.9 Al | 98.4 | – | 1.6 |
| Sample 22, ground bulk metal, SA 2: 200 µm × 200 µm | 86.8 | – | 2.2 | 3.1 | 0.4 | 7.4 | – | 97.5 | – | 2.5 |
| Sample 22, SA 3: 200 µm × 200 µm | 86.1 | – | 2.5 | 2.5 | 0.3 | 8.5 | – | 97.2 | – | 2.8 |
| Sample 22, SA 4: 200 µm × 200 µm | 88.3 | – | 2.0 | 2.7 | 0.4 | 6.6 | – | 97.8 | – | 2.2 |
| Sample 22, SA 5: 100 µm × 100 µm | 79.1 | – | 2.1 | 4.4 | 0.9 | 13.5 | | 97.4 | – | 2.6 |
| Sample 22, SA 6: 100 µm × 100 µm | 74.2 | – | 2.5 | 6.6 | 1.3 | 14.5 | 1.0 Al | 96.7 | – | 3.3 |
| Sample 22, ground bulk, SA 7 100 µm × 100 µm | 80.1 | 1.6 | 1.0 | 8.5 | 1.1 | 7.4 | 0.2 Al | 96.9 | 1.9 | 1.2 |
| Sample 23, SA 1: 500 µm × 500 µm | 75.3 | – | 1.8 | 4.6 | 1.5 | 16.9 | – | 97.7 | – | 2.3 |
| Sample 23, SA 2: 300 µm × 300 µm | 76.4 | 1.0 | 2.0 | 5.2 | 2.2 | 13.2 | – | 96.2 | 1.3 | 2.5 |
| Sample 23, SA 3: 100 µm × 100 µm | 77.4 | – | 1.2 | 1.7 | 0.4 | 19.3 | – | 98.5 | – | 1.5 |

**Table S16.** SEM-EDS analysis results of the two coins (Sidonian and Philistian), where SA represents the scanned area.

| **Sample** | **Composition (wt %)** | | | | | | | |
|---|---|---|---|---|---|---|---|---|
| | **Surface** | | | | | | **Silver alloy** | |
| | **Ag** | **Cu** | **O** | **Si** | **Cl** | **Others** | **Ag** | **Cu** |
| Sample 24 (Sidonian coin), reverse, SA 1: 100 µm × 100 µm | 76.8 | – | 13.9 | 1.2 | 5.4 | 1.4 Na, 0.4 Al, 0.5 Ca, 0.4 S | 100 | – |
| Sample 24, reverse, SA 2: 100 µm × 100 µm | 74.6 | – | 16.0 | 1.9 | 5.5 | 1.6 Na | 100 | – |
| Sample 24, reverse, SA 3 (fractured surface): 50 µm × 50 µm | 90.7 | 0.9 | 8.4 | – | – | – | 99.0 | 1.0 |
| Sample 24, reverse, SA 4 (fractured surface): 100 µm × 100 µm | 92.1 | – | 7.9 | – | – | – | 100 | – |
| Sample 24, obverse, SA 1 (fractured surface): 100 µm × 100 µm | 73.5 | – | 13.3 | 1.0 | 10.1 | 1.8 Na, 0.4 Al | 100 | – |
| Sample 24, obverse, SA 2 (fractured surface): 200 µm × 200 µm | 75.5 | – | 11.6 | 0.8 | 10.0 | 1.5 Na, 0.3 Al, 0.5 Ca | 100 | – |
| Sample 24, obverse, SA 3 (fractured surface): 100 µm × 100 µm | 84.4 | 0.6 | 13.0 | 0.9 | 0.7 | 0.4 Al | 99.3 | 0.7 |
| Sample 24, obverse, SA 4 (fractured surface): 20 µm × 20 µm | 85.0 | 0.9 | 11.9 | 0.6 | – | 0.7 Na, 0.6 Mg, 0.3 Al | 99.0 | 1.0 |
| Sample 25 (Philistian coin), reverse, SA 1: 200 µm × 200 µm | 82.3 | – | 9.2 | 1.5 | 6.1 | 0.3 Na, 0.5 Al | 100 | – |
| Sample 25, reverse, SA 2: 100 µm × 100 µm | 82.6 | – | 8.5 | 1.2 | 7.2 | 0.4 Al | 100 | – |
| Sample 25, obverse, SA 1: 100 µm × 100 µm | 84.4 | 1.0 | 6.4 | 0.9 | 7.1 | 0.2 Al | 98.8 | 1.2 |
| Sample 25, obverse, SA 2: 200 µm × 200 µm | 83.5 | – | 8.0 | 1.1 | 7.2 | 0.3 Al | 100 | – |
| Sample 25, obverse, SA 1: 100 µm × 100 µm | 90.4 | – | 4.7 | 0.5 | 3.9 | 0.4 Al | 100 | – |
